# ﻿Exploring the relationship between bats (Mammalia, Chiroptera) and ectoparasitic flies (Diptera, Hippoboscoidea) of the Orinoquia Region in South America

**DOI:** 10.3897/zookeys.1179.103479

**Published:** 2023-09-08

**Authors:** Erika M. Ospina-Pérez, Fredy A. Rivera-Páez, Héctor E. Ramírez-Chaves

**Affiliations:** 1 Doctorado en Ciencias – Biología, Facultad de Ciencias Exactas y Naturales, Universidad de Caldas, Calle 65 No. 26-10, 170004, Manizales, Caldas, Colombia Universidad de Caldas Manizales Colombia; 2 Grupo de Investigación en Genética, Biodiversidad y Manejo de Ecosistemas (GEBIOME), Departamento de Ciencias Biológicas, Facultad de Ciencias Exactas y Naturales, Universidad de Caldas, Calle 65 No. 26-10, 170004, Manizales, Caldas, Colombia Universidad de Caldas Manizales Colombia; 3 Centro de Museos, Museo de Historia Natural, Universidad de Caldas, Calle 58 No. 21-50, 170004, Manizales, Caldas, Colombia Universidad de Caldas Manizales Colombia

**Keywords:** Bat flies, ecological networks, ecomorphology, Neotropics, Nycteribiidae, parasitism, Streblidae

## Abstract

Bat flies (Nycteribiidae and Streblidae) have been used to study co-evolutionary patterns between ectoparasites and bats. In the world, Nycteribiidae and Streblidae are represented by approximately 276 and 237 species, respectively. In regions such as the Orinoquia located in the north of South America (Colombia and Venezuela), the richness of bats is high (more than 100 documented species), but studies on Nycteribiidae and Streblidae are scarce and discontinuous. To contribute to the knowledge of ectoparasitic flies in the Orinoquia, records of flies and their interactions with bats were reviewed, including new records and associations using interaction networks. We documented 124 species of Streblidae and only 12 of Nycteribiidae for the Orinoquia in approximately 102 bat species reported in Colombia and Venezuela. New records for six species of bat flies in Colombia were found (*Mastopteraguimaraesi*, *Noctiliostreblamaai*, *Paradyschiriaparvuloides*, *Trichobiusjubatus*, *Trichobiusparasiticus*, and *Basiliaferrisi*) associated with six species of bats (*Cynomopsplanirostris*, *Desmodusrotundus*, *Myotishandleyi*, *Molossusrufus*, *Noctilioalbiventris*, and *Phyllostomushastatus*). The bat-ectoparasite interaction networks in the Orinoquia revealed a pattern of antagonistic relationships, with high specialization, modularity, and low connectivity and nesting. The identified networks are between bat fly species belonging to different ecomorphological groups with unique host species. This supports the idea of ecological niche partitioning among ectoparasitic bat flies and hosts. Our study expanded the knowledge of the distribution of some fly species and the associations with bat hosts in Colombia, by presenting morphological descriptions and new observations, which are key to understanding the ecology, diversity, and distribution of these species.

## ﻿Introduction

Bat flies (Diptera: Hippoboscoidea) are obligate blood-feeding ectoparasites of bats (Chiroptera) and an interesting evolutionary system for studying co-evolutionary patterns between hosts and ectoparasites (Porter et al. 2021). Taxonomically bat flies are divided into two cosmopolitan families, Nycteribiidae and Streblidae (Wenzel et al. 1966; Dick and Patterson 2006; Patterson et al. 2007), but the latter is considered paraphyletic with New World and Old-World lineages (Dittmar et al. 2006; Petersen et al. 2007). Both families of bat flies show greater diversity in tropical latitudes and are less diverse in subtropical and temperate regions (Dick and Dittmar 2014). However, Nycteribiids (three subfamilies, 11 genera and ~ 276 species for 2018) are more numerous in the Eastern Hemisphere, while, for the Streblids (five subfamilies, 33 genera, and ~ 237 species for 2018) the richness is greater in the Western Hemisphere (Dick and Patterson 2006; Dick and Miller 2010; Soares et al. 2013; Graciolli and Dick 2018). Based on dispersion-vicariance analysis, Dittmar et al. (2006) suggested that the Neotropical region is the ancestral area for all New World Streblidae, while the Oriental region is considered the ancestral area of Nycteribidae and Old-World Streblidae.

Both Nycteribiidae and Streblidae present a wide variety of morphological and physiological adaptations for their ectoparasitic lifestyle, among which adenotrophic viviparity stands out (Hagan 1951; Lehane 2005; Dick and Miller 2010; Dick and Dittmar 2014). In this process, the larvae develop individually in the female oviduct until the third instar (called prepupa) (Hagan 1951). Later, when the development of the prepupa is complete, female bat flies of both families deposit a single prepupa on substrates in the roost (Petersen et al. 2007; Dick and Dittmar 2014). Once the prepupa is deposited, it immediately forms a puparium, which, after a pupal stage, emerges as an unfed adult fly (teneral) and must locate and colonize a host (Ching and Marshall 1968).

In general, the life strategy of bat flies reflects their association with bats, and it has been found that some Streblidae flies can form specific parasite assemblages for each bat species due to their high host specificity (Wenzel 1976; Hiller et al. 2018). Each community can consist of two to five fly species, and each of them shows a preference for a specific part of the bat’s body (Wenzel 1976; Ter Hofstede et al. 2004; Dick and Gettinger 2005; Tello et al. 2008; Patterson et al. 2009). Dick (2005) defined three ecomorphological groups of bat flies based on behavioral observations and morphological traits: 1) “wing crawler” primarily live on wing membranes, with non-flattened bodies and legs of the same size, 2) “fur runners” that live on the hairy membranes of the body and move by running on the fur, with well-developed wings and long legs (especially the hind legs), and 3) “fur swimmers” that inhabit areas of long fur such as the neck and have compressed bodies and heads, and possessing Ctenidia. Hiller et al. (2018) supported this classification, focusing on differences in the morphology and size of the hind legs, and found evidence of density-dependent competition among species within the same ecomorphological group.

In Neotropical countries with a high number of bat species such as Colombia (~ 217 species) and Venezuela (172 species) (Delgado-Jaramillo et al. 2016; Ramírez-Chaves et al. 2021), studies on Streblidae and Nycteribiidae are few and discontinuous (e.g., Tamsitt and Fox 1970; Marinkelle and Grose 1981; Guerrero 1994). In recent years, new research has expanded the knowledge about these ectoparasites in both countries (Herrera-Sepúlveda 2013; Tarquino-Carbonell 2014; Tarquino-Carbonell et al. 2015; Dick et al. 2016; Durán et al. 2018; Calonge-Camargo and Pérez-Torres 2018; Guerrero 2019; Liévano-Romero et al. 2019; Raigosa Álvarez et al. 2020; Cañizales and Guerrero 2022). Nonetheless, there are very few studies in the Orinoquia Region, a hydrographic basin that converges into the Orinoco River, covering an area of almost 989,000 km^2^ (Domínguez 1998), and shared by both countries (Guimarães 1972; Wenzel 1976; Dick et al. 2016; Liévano-Romero et al. 2019; López Rivera et al. 2022). Approximately 65% of this area is located in Venezuela, while the remaining 35% is in Colombia (León 2005). The Venezuelan portion extends from the Andes and the Cordillera de la Costa to the north-western bank of the Orinoco River, forming most of the Venezuelan plains and the delta Orinoco (Domínguez 1998; León 2005). The Orinoquia is one of the most diverse areas in terms of mammal diversity with 318 species, including 150 species of Chiroptera (Ferrer Pérez et al. 2009; Pardo and Rangel-Ch. 2014).

Of the 125 Streblidae species reported in Colombia and Venezuela (81 and 107 respectively; Wenzel 1976; Tarquino-Carbonell et al. 2015; Dick et al. 2016; Duran et al. 2017; Calonge-Camargo and Pérez-Torres 2018; Guerrero 2019; Liévano-Romero et al. 2019; Ascuntar-Osnas et al. 2020; Raigosa Álvarez et al. 2020; López Rivera et al. 2022), 124 have been documented for the Orinoquia in ~ 96 bat species of the families Emballonuridae, Molossidae, Mormoopidae, Natalidae, Noctilionidae, Phyllostomidae, and Vespertilionidae (Wenzel 1976; Dick et al. 2016; Guerrero 2019; Liévano-Romero et al. 2019; López Rivera et al. 2022). Similarly, of the 16 Nycteribiidae reported in Colombia and Venezuela (11 and 13, respectively; Wenzel and Tipton 1966; Graciolli et al. 2016; Pastrana-Montiel et al. 2019; Raigosa Álvarez et al. 2020; López Rivera et al. 2022), 12 have been found in the Orinoquia (two in Colombia, 12 in Venezuela; Wenzel and Tipton 1966; Graciolli et al. 2016; López Rivera et al. 2022). Besides the high number of bats and bat flies documented in the Orinoquia, the interaction structure that might be evaluated using ecological or interaction networks (Dormann et al. 2009), has not been addressed. Interaction networks are formed by interactions (links) between species (nodes) that make up a community (network); they are considered as a synthesis tool in the study of ecological interactions that allow understanding the functioning of megadiverse systems (Blüthgen et al. 2006). The study of the host-parasite specificity interactions is essential to understand the mechanisms behind parasitism and its relationship with biodiversity functioning (Frainer et al. 2018) since parasites play an important role in the regulation of populations of host species (Poulin et al. 2006). For those reasons, and to contribute to the knowledge of ectoparasitic flies of the Orinoquia, we present novel records (including morphological description of ectoparasitic flies) and new associations with bats from this region in Colombia, and a review of records of ectoparasitic flies and the interactions with hosts bats in the whole Orinoquia (Colombia-Venezuela).

## ﻿Materials and methods

### ﻿Study Area

**Figure 1.**
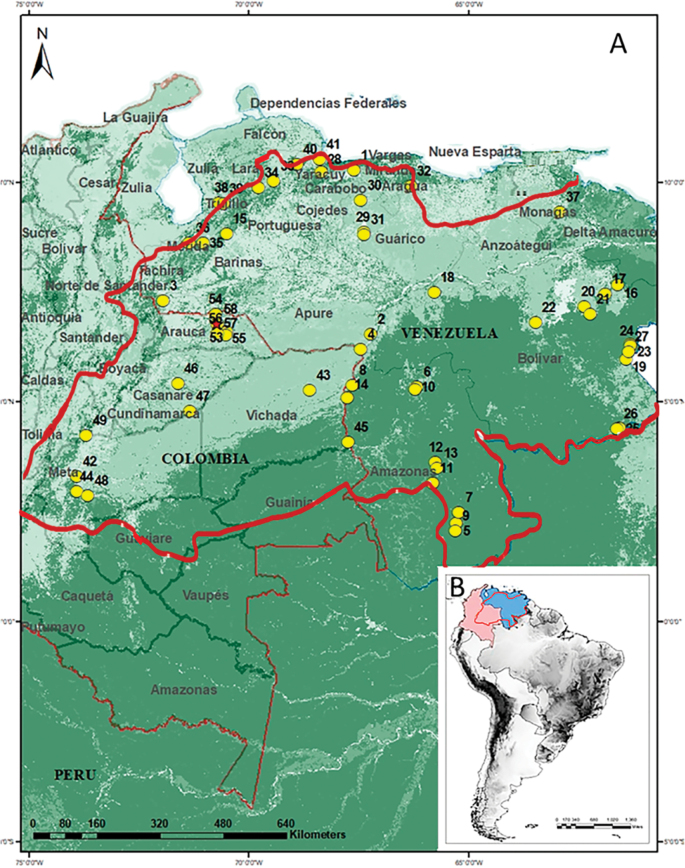
Locality records of Nycteribiidae and Streblidae in the Orinoquia Region in South America **A** Orinoquia **B** South America highlighting Colombia (pink) and Venezuela (blue). The red line delimits the Orinoco basin according to Domínguez (1998); the yellow circles indicate 58 localities reported in the literature (Table 1) and the red star indicate the new records for the Department of Arauca, Colombia (Table 2). Detailed information is found in the Suppl. material 1: table S1.

The Orinoquia Region shared by Colombia and Venezuela is a hydrographic basin, with waters that converge in the Orinoco River (Domínguez 1998). The Orinoco basin covers an area of almost 989,000 km^2^ of which 643,480 km^2^ (65%) are located in Venezuela and 35% in Colombia (Fig. 1). In Colombia, the Orinoquia extends from the eastern slopes of the Cordillera Oriental of Colombia. In Venezuela, the Orinoquia extends from the Venezuelan Andes and the Cordillera de la Costa to the north-western bank of the Orinoco River, forming most of the Venezuelan plains and the delta Orinoco (León 2005). The Orinoco basin features isothermal climates with minimal temperature fluctuations throughout the year. This is typical of the inter-tropical zone and is characterized by a mere 3 °C difference between the average temperatures of the warmer and cooler months. In the lowlands, which reach up to 800 m above sea level, there are five primary climate types, including jungle, savanna, semi-desert, and desert proper (León 2005).

### ﻿Richness of bats and ectoparasite flies in the Orinoco Region and new records from Colombia

To gather the information on ectoparasitic flies associated with bats from Orinoquia Region (Fig. 1), we reviewed the information available in the literature retrieved from Science Direct, Web of Science, SciELO Scopus, and Google Scholar search engines using the keywords ((Fly*) OR (Flies*) AND ((Streblidae*) OR (Nycteribiidae*) AND (bat*) AND (Colombia*) OR (Venezuela*)), without temporal restrictions. We also analyzed the sources cited and referenced in the publications to obtain more data and information for the compilation of the interaction networks. To update the bats taxonomy, we used recent check list and online resources (e.g., Ramírez-Chaves et al. 2021; Mammal Diversity Database 2022). To include an article in our study, we used the following criteria: bat species found in contact with ectoparasitic dipterans from a locality (including latitude and longitude) within the Orinoquia Region. It is relevant to highlight that when reviewing the associations, we consider previously reported bat fly species complexes (sensu Wenzel 1976), which are believed to have a generalist behavior. In publications lacking geographic coordinates we extrapolated this information using Google Earth (https://www.google.com/intl/es/earth/), when possible. To assess the quality of the published bat flies-host associations, we collected information on the abundances of flies found on the hosts, as well as the specific location and date of each report. To identify possible non-primary associations due to contamination transfers, two important criteria were applied (Dick 2005): 1) non-primary associations that represented ≤ 5% of the total records, and 2) ectoparasitic flies had to be collected in the presence of the primary host at the same location and date (Table 1 and Suppl. material 1: table S2). Additionally, we reviewed the non-primary associations reported for the Department of Arauca in previous works (e.g., López Rivera et al. 2022), based on specimens deposited in the Collection of Ectoparasites (**Ec**) at the Museo de Historia Natural of the Universidad de Caldas (**MHN-UCa**).

**Table 1.** Records of flies in bats in Orinoquia from 1911 to June 2023. Localities respresents the number of localities in which the species has been documented. Letters in parentheses following the fly species indicate host-specificity (HS) behavior: M: monoxene (found in only one host species); O: oligoxene (found in different species of bat hosts, but of the same genus); Pl: pleioxene (found in different species and genera of bat hosts, but from the same family); Po: polyxene (found in different host bat species and genera and in different families). The hosts were arranged in descending order from primary hosts, transitional to incidental, the hosts marked with an asterisk are not primary (≤5% of the total records) and those followed by the letter (C) are possible associations resulting from contamination by manipulation when they are collected in the presence of the primary host in the same place and date (Dick 2005), however, as they are congeners of the primary host, this definition should be considered. The other associations are difficult to define if they are ecological or contamination due to the low number of records.

Bat-flies taxa	Bats	Country (Department/State)	Localities	References
**Family Streblidae**				
**Subfamily Nycterophiliinae**				
*Nycterophiliacoxata* (Pl)	*Pteronotusparnelli* (probably *P.fuscus*), *Phyllostomuselongatus, *Artibeusplanirostris, *Eumopsglaucinus, *Pteronotusdavyi (C) and *Pteronotusgymnonotus (C)	Venezuela (Amazonas, Bolívar, Guárico, Monagas, Trujillo and Yaracuy)	8	Wenzel 1976
*Nycterophiliafairchildi* (O)	*Pteronotusdavyi*, *Pteronotusgymnonotus*, *Pteronotusparnelli (probably *P.fuscus*) (C) and *Platyrrhinushelleri (C)	Venezuela (Yaracuy)	1	Wenzel 1976
*Nycterophiliamormoopsis*	*Mormoopsmegalophylla*	Venzuela (Yaracuy)	1	Wenzel 1976
*Nycterophiliaparnelli* (M)	*Pteronotusparnelli* (probably *P.fuscus*), *Lonchorhinaorinocensis (C), *Lonchorhinaaurita, *Pteronotusrubiginosus and *Sturniratildae (C)	Colombia (Vichada) and Venezuela (Amazonas, Apure, Bolívar Carabobo and Yaracuy)	14	Wenzel 1976; Dick et al. 2016
*Phalcophilapuliciformes*	*Lonchophyllarobusta* and *Artibeusplanirostris*	Venezuela (Barinas)	1	Wenzel 1976
**Subfamily Trichobiinae**				
*Anatrichobiusscorzai* (O)	*Myotisoxyotus*, *Myotiskeaysi* and *Lonchophyllarobusta	Venezuela (Barinas, Bolívar and Carabobo)	4	Wenzel 1976
*Aspidopterafalcata* (O)	*Sturniralilium* (probably *S.giannae*), *Sturniraludovici*, *Sturniratildae*, *Dermanuracinerea (C), *Artibeusobscurus (C), *Artibeusplanirostris (C), *Carolliaperspicillata (C), *Phyllostomushastatus (C), and *Urodermabilobatum (C)	Venezuela (Amazonas, Apure, Barinas, Carabobo, Guárico, Monagas, Trujillo and Yaracuy)	22	Wenzel 1976
*Aspidopteraphyllostomatis* (O)	, *Artibeusplanirostris*, *Artibeuslituratus, *Phyllostomusdiscolor (C) and *Sturniragiannae (C)	Colombia (Arauca, Meta, Vichada and Casanare) and Venezuela (Amazonas, Apure, Barinas, Bolivar, Carabobo, Guárico, Monagas, Trujillo, Yaracuy)	24	Wenzel 1976; Dick et al. 2016; Liévano-Romero et al. 2019; López Rivera et al. 2022
*Aspidopteradelatorrei* (Pl)	*Sturniragiannae* and Sturniracf.parvidens and *Artibeusplanirostris,	Colombia (Arauca and Meta)	2	Dick et al. 2016; López Rivera et al. 2022
*Exastinionclovisi* (O)	*Anouracaudifer*, *Anourageoffroyi*, *Anouralatidens*, *Artibeusplanirostris (C) and *Peropteryxmacrotis (C)	Venezuela (Amazonas, Barinas, Bolivar, Carabobo, Guarico, Monagas) and Colombia (Arauca)	14	Wenzel 1976; López Rivera et al. 2022
*Exastinionoculatum* (M)	*Anouracultrata*	Venezuela (Aragua)	1	Wenzel 1976
*Exastiniondeceptivum* (M)	*Anourageoffroyi*	Venezuela (Merida and Monagas)	1	Wenzel 1976
***Mastopteraguimaraesi*** (M)	***Phyllostomushastatus***	Colombia(**Arauca**) and Venezuela (Apure, Barinas, Trujillo and Yaracuy)	6	**This study**; Wenzel 1976
*Mastopteraminuta* (O)	*Lophostomasilvicola* and *Tonatiasaurophila*	Colombia (Meta, Casanare and Vichada) and Venezuela (Amazonas and Trujillo)	7	Wenzel 1976; Dick et al. 2016; Liévano-Romero et al. 2019
*Mastopteraminuta* s.l. (Pl)	*Lophostomabrasiliense*, *Lophostomacarrikeri*, *Lophostomasilvicola*, *Phyllostomushastatus*, *Phyllostomuselongatus*, *Anoura sp. (C), *Artibeusobscurus (C), *Artibeusplanirostris* (C) and *Artibeuslituratus (C)	Venezuela (Amazonas, Trujillo and Yaracuy)	10	Wenzel et al. 1976
*Megistopodaproxima* (Po)	*Sturniragiannae*, *Sturniraparvidens*, *Sturnira* sp., *Carolliaperspicillata (C) and *Noctilioalbiventris*	Colombia (Arauca)	2	López Rivera et al. 2022
*Megistopodaaranea* (O)	*Artibeuslituratus* and *Artibeusplanirostris*	Colombia (Arauca and Casanare) and Venezuela (Amazonas, Apure, Barinas, Bolívar, Carabobo, Guarico, Monagas, Trujillo and Yaracuy).	28	Wenzel 1976; Liévano-Romero et al. 2019; López López-Rivera et al. 2022
*Neotrichobiusbisetosus* (O)	*Artibeusobscurus* and *Artibeusplanirostris (C)	Colombia (Meta) and Venezuela (Amazonas and Bolívar)	9	Wenzel 1976; Dick et al. 2016
*Neotrichobiusdelicatus* (M)	*Vampyressathyone*	Colombia (Meta) and Venezuela (Barinas, Carabobo and Yaracuy)	16	Wenzel 1976; Dick et al. 2016
*Neotrichobiusdelicatus* s.l. (Pl)	*Dermanuracinerea*, *Rhinophyllapumilio*, *Artibeus sp., *Artibeusplanirostris, *Platyrrhinushelleri (C) and *Urodermamagnirostrum	Venezuela (Amazonas, Apure, Barinas, Bolívar, Carabobo and Guárico)	16	Wenzel 1976
*Neotrichobiusectophyllae* (M)	*Mesophyllamacconnelli*	Venezuela (Amazonas)	1	Wenzel 1976
*Neotrichobiusstenopterus* (Po)	*Dermanuracinerea*	Venezuela (Trujillo)	1	Wenzel 1976
*Noctiliostreblaaitkeni* (M)	*Noctilioleporinus* and *Saccopteryxbilineata	Colombia (Meta) and Venezuela (Amazonas, Bolivar, Monagas, Yaracuy)	6	Wenzel 1976; Dick et al. 2016
***Noctiliostreblamaai*** (Po)	***Noctilioalbiventris***, *Molossusrufus (C), and *Sturniragiannae*	Colombia (**Arauca**) and Venezuela (Amazonas, Apure, Bolivar, Monagas, Yaracuy)	9	**This study**; Wenzel 1976; López Rivera et al. 2022
*Noctiliostrebladubia* (M)	Unidentified host	Colombia (Meta)	1	Dick et al. 2016
*Noctiliostreblatraubi* (M)	*Noctilioleporinus*	Venezuela (Guárico and Yaracuy)	2	Wenzel 1976
*Paradyschiriacurvata* (M)	*Noctilioalbiventris*, *Desmodusrotundus (C), *Molossusrufus (C) and *Trachopscirrhosus (C)	Venezuela (Apure)	2	Wenzel 1976
*Paradyschiriafusca* (M)	*Noctilioleporinus*	Colombia (Meta and Casanare) and Venezuela (Amazonas, Bolivar, Monagas)	7	Wenzel 1976; Dick et al. 2016
*Paradyschirialineata* (M)	*Noctilioleporinus*, *Noctilioalbiventris (C) and *Pteronotusparnelli (probably *P.fuscus*) (C)	Venezuela (Guarico and Yaracuy)	2	Wenzel 1976
*Paradyschiriaparvula* (M)	*Noctilioalbiventris*, *Molossusaztecus (C) and *Molossusrufus (C)	Colombia (Vichada) and Venezuela (Amazonas, Apure, Monagas and Yaracuy)	5	Wenzel 1976; Dick et al. 2016
***Paradyschiriaparvuloides*** (M)	***Noctilioalbiventris*** and *Cynomopsplanirostris (C)	Colombia (**Arauca**) and Venezuela (Apure and Trujillo)	3	**This study**; Wenzel 1976; López Rivera et al. 2022
*Parastreblahandleyi* (M)	*Trinycterisnicefori*	Venezuela (Bolivar)	1	Wenzel 1976
*Paratrichobiusdunni* (O)	*Urodermabilobatum*, *Urodermamagnirostrum* and *Desmodusrotundus (C),	Venezuela (Amazonas, Apure, Barinas, Bolívar, Trujillo and Yaracuy)	12	Wenzel 1976
*Paratrichobiuslongicrus* (M)	*Artibeuslituratus*	Colombia (Meta) and Venezuela (Amazonas, Apure, Aragua, Barinas,Bolívar, Carabobo, Trujillo and Yaracuy)	16	Wenzel 1976; Dick et al. 2016
*Paratrichobiuslongicrus* s.l.	*Platyrrhinusaurarius*, *Platyrrhinusumbratus, *Platyrrhinusvittatus, *Desmodusrotundus and *Carolliaperspicillata	Venezuela (Amazonas, Apure, Barinas, Bolívar, Monagas)	5	Wenzel 1976
*Paratrichobiuslowei* (O)	*Dermanuracinerea*	Venezuela (Bolívar)	1	Wenzel 1976
*Paratrichobiussanchezi* (M)	*Enchistheneshartii*	Venezuela (Carabobo, Guárico and Monagas)	3	Wenzel 1976
*Paratrichobiussalivini* (M)	*Chirodermasalvini*	Venezuela (Carabobo and Monagas)	1	Wenzel 1976
*Paratrichobiussalivini* s.l. (Pl)	*Chirodermatrinitatum*, *Chirodermavillosum*, *Platyrrhinushelleri*, *Vampyriscusbidens* and *Vampyrodescaraccioli	Venezuela (Amazonas, Apure, Barinas, Carabobo and Yaracuy)	9	Wenzel 1976
*Pseudostreblagreenwelli*	*Lophostomabrasiliense* and *Tonatiamaresi*	Colombia (Casanare) and Venezuela (Amazonas)	2	Wenzel 1976; Liévano-Romero et al. 2019
*Pseudostreblaribeiroi*	*Lophostomasilvicola*	Venezuela (Amazonas)	2	Wenzel 1976
*Pseudostreblasparsisetis* (M)	*Lophostomacarrikeri*	Colombia (Meta) and Venezuela (Amazonas)	3	Wenzel 1976; Dick et al. 2016
*Speiseriaambigua* (O)	*Carolliaperspicillata* and *Carolliabrevicauda (C)	Colombia (Arauca, Meta and Casanare) and Venezuela (Amazonas Apure, Barinas Bolivar, Carabobo, Guárico, Monagas, Trujillo and Yaracuy)	33	Wenzel 1976; Liévano-Romero et al. 2019; López Rivera et al. 2022
*Speiseriamagnioculus* (M)	*Trachopscirrhosus*	Venezuela (Amazonas and Bolivar)	5	Wenzel 1976
*Speiseriapeytoni* (M)	*Carolliabrevicauda*	Colombia (Meta) and Venezuela (Apure, Barinas, Bolivar and Carabobo)	7	Wenzel 1976; Dick et al. 2016
*Stizostreblalongirostris*	*Lophostomacarrikeri*	Colombia (Meta) and Venezuela (Amazonas)	2	Wenzel 1976; Dick et al. 2016
*Trichobiusaffinis* (M)	*Lophostomabrasiliense*	Venezuela (Amazonas and Apure)	2	Wenzel 1976
*Trichobiusanducei* (M)	*Carolliaperspicillata*	Colombia (Arauca)	1	López Rivera et al. 2022
*Trichobiusangulatus* (M)	*Platyrrhinusauraritus*	Colombia (Meta) and Venezuela (Amazonas and Bolívar)	3	Wenzel 1976; Dick et al. 2016
*Trichobiusassimilis* (M)	*Artibeusplanirostris* and *Platyrrhinusauraritus (C)	Venezuela (Amazonas and Bolívar)	4	Wenzel 1976
*Trichobiusbilobus* (M)	*Pteronotusgymnonotus*	Venezuela (Trujillo and Yaracuy)	2	Wenzel 1976
*Trichobiuscaecus* (M)	*Pteronotusparnellii* (probably *P.fuscus*), *Pteronotusrubiginosus*, *Anourageoffroyi (C), *Artibeusobscurus (C), *Artibeuslituratus (C), *Desmodusrotundus (C), *Macrophyllummacrophyllum (C), *Myotiskeaysi*, *Pteronotusdavyi (C), *Rhynchonycterisnaso (C), *Trachopscirrhosus (C) and *Urodermabilobatum (C)	Colombia (Vichada) and Venezuela (Amazonas, Apure, Aragua, Bolívar, Carabobo, Guárico, Monagas and Yaracuy)	26	Wenzel 1976; Dick et al. 2016
*Trichobiuscostalimai* (M)	*Phyllostomusdiscolor*, *Phyllostomuselongatus and *Eptesicusorinocensis (C)	Colombia (Arauca and Meta) and Venezuela (Amazonas, Aragua, Barinas, Bolívar, Guárico, Carabobo, Monagas and Trujillo)	19	Wenzel 1976; Dick et al. 2019; López Rivera et al. 2022
*Trichobiusdiaemi* (M)	*Diaemusyoungii*	Colombia (Guainía) and Venezuela (Amazonas)	4	Wenzel 1976; Dick et al. 2016
*Trichobiusdybasi*	Unidentified host	Colombia (Meta)	1	Dick et al. 2016
*Trichobiusdiphyllae*	*Diphyllaecaudata*	Colombia (Vaupes) and Venezuela (Aragua)	1	Wenzel 1976; Dick et al. 2016
*Trichobiusdugesii* (O)	*Glossophagalongirostris*, *Glossophagasoricina*, *Carolliabrevicauda, *Trinycterisnicefori and *Platyrrhinushelleri (C)	Colombia (Meta and Vichada) and Venezuela (Amazonas, Apure, Barinas, Bolívar, Guárico, Monagas, Trujillo and Yaracuy)	25	Wenzel 1976; Dick et al. 2016
*Trichobiusdugesioidesdugesioides* (Pl)	*Trachopscirrhosus*, *Chrotopterusauritus (C), *Desmodusrotundus (C), *Macrophyllummacrophyllum (C), *Phyllostomusdiscolor* (C), *Sphaeronycteristoxophyllum (C) and *Lophostomasilvicola (C)	Colombia (Meta) and Venezuela (Amazonas, Apure, Barinas, Bolívar, Guárico, Trujillo and Yaracuy)	29	Wenzel 1976; Dick et al. 2016
*Trichobiusdugesioidesphyllostomus*	*Phyllostomuselongatus* and *Phyllostomushastatus	Venezuela (Amazonas and Bolívar)	6	Wenzel 1976; Guerrero 1998
*T.dugesioides* (probably *Trichobiusanducei*) (O)	*Carolliaperspicillata* and *Carolliabrevicauda (C)	Venezuela (Amazonas, Apure, Barinas, Bolívar and Yaracuy	10	Wenzel 1976; Guerrero 1998
*Trichobiusethophallus* (M)	*Lonchorhinaorinocensis*	Venezuela (Amazonas and Apure)	5	Wenzel 1976
*Trichobiusflagellatus* (O)	*Lonchorhinaaurita* and *Lonchorhinaorinocensis	Venezuela (Amazonas, Barinas, Bolívar and Trujillo)	6	Wenzel 1976
*Trichobiusfurmani*	Unidentified host	Colombia (Meta)	1	Dick et al. 2016
*Trichobiusgalei* (M)	*Natalustumidirostris*	Venezuela (Aragua and Bolívar)	2	Wenzel 1976
*Trichobiushandleyi* (M)	Micronycterisminuta* and *Phyllostomuselongatus (C)	Venezuela (Amazonas, Apure, Bolívar, Guárico, Monagas and Trujillo)	7	Wenzel 1976
*Trichobiushispidus* (M)	*Sturnirabidens*	Venezuela (Mérida)	1	Wenzel 1976
*Trichobiusimitator*	*Anoura* sp.	Venezuela (Bolívar)	1	Wenzel 1976
*Trichobiusjoblingi* (Pl)	*Carolliaperspicillata*, *Phyllostomuselongatus*, *Carolliabrevicauda*, *Desmodusrotundus (C), *Phyllostomushastatus (C) and *Platyrrhinusfusciventris	Colombia (Arauca, Casanare, Meta and Vaupes) and Venezuela (Amazonas, Apure, Barinas, Bolívar, Guárico, Monagas, Trujillo and Yaracuy)	53	Wenzel 1976; Dick et al. 2016; Liévano-Romero et al. 2019
*Trichobiusjohnsonae* (O)	*Pteronotusgymnonotus*, *Pteronotusdavyi*, *Pteronotuspersonatus* and *Noctilioalbiventris (C)	Venezuela (Aragua, Bolívar and Yaracuy)	4	Wenzel 1976
***Trichobiusjubatus*** (M)	***Molossusrufus***, *Molossuspretiosus* and *Molossusmolossus	Colombia (**Arauca**) and Venezuela (Amazonas, Apure and Monagas)	8	**This study**; Wenzel 1976; López Rivera et al. 2022
*Trichobiuskeemani*	*Micronycterismegalotis*, *Micronycterismicrotis* and *Carolliaperspicillata*	Venezuela (Amazonas, Apure and Barinas)	4	Wenzel 1976
*Trichobiusleiomotus* (M)	*Mormoopsmegalophylla*	Venezuela (Bolívar and Yaracuy)	2	Wenzel 1976
*Trichobiuslionycteris* (M)	*Lionycterisspurrelli*, *Carolliaperspicillata (C), *Molossusaztecus (C), *Sturniralilium (probably *S.giannae*) (C) and *Platyrrhinushelleri (C)	Colombia (Meta) and Venezuela (Amazonas, Apure and Bolívar)	11	Wenzel 1976; Dick et al. 2019
*Trichobiuslonchophyllae* (O)	*Lonchophyllarobusta*, *Lonchophyllaorienticollina*, *Myotishandleyi, *Anouralatidens (C) and *Sturniralilium (probably *S.giannae*) (C)	Colombia (Arauca) and Venezuela (Barinas)	5	Wenzel 1976; López Rivera et al. 2022
*Trichobiuslongipes* (O)	*Phyllostomushastatus*, *Phyllostomuselongatus*, *Artibeusplanirostris (C), *Desmodusrotundus (C), *Molossusrufus, *Rhynchonycterisnaso (C) and *Urodermabilobatum (C)	Colombia (Arauca) and Venezuela (Amazonas, Apure, Aragua, Barinas, Bolívar, Carabobo, Guárico, Monagas, Trujillo and Yaracuy)	23	Wenzel 1976; López Rivera et al. 2022
*Trichobiuslongipilis* (M)	*Pteropteryxmacrotis*	Venezuela (Bolívar)	2	Wenzel 1976
*Trichobiusmacrophylli* (M)	*Macrophyllummacrophyllum*	Venezuela (Amazonas, Apure, Bolívar and Guárico)	6	Wenzel 1976
*Trichobiuspallidus*	*Furipterushorrens*	Venezuela (Amazonas)	1	Wenzel 1976
*Trichobiusparasparsus* (M)	*Pteronotusparnelli* (probably *P.fuscus*), *Carolliaperspicillata (C), *Sturniralilium (probably *S.giannae*) (C), *Sturniratildae (C) and *Urodermaconvexum (C)	Venezuela (Amazonas, Apure, Bolívar and Yaracuy) and Colombia (Vichada)	17	Wenzel 1976; Dick et al. 2016
***Trichobiusparasiticus*** (Pl)	***Desmodusrotundus***, *Carolliaperspicillata*, *Chirodermavillosum* and *Platyrrhinusumbratus*	Colombia (Meta and **Arauca**) and Venezuela (Amazonas, Apure, Barinas, Bolívar, Carabobo, Guárico, Monagas, Trujillo and Yaracuy)	39	**This study**; Wenzel 1976; Dick et al. 2016
*Trichobiuspermilis* (O)	*Carolliabrevicauda*, *Carolliaperspicillata* and *Phyllostomuselongatus	Venezuela (Apure, Barinas, Bolívar, Carabobo and Monagas)	10	Wenzel 1976
*Trichobiuspetersoni* (O)	*Sturniraerythromos* and *Sturnirabogotensis	Venezuela (Barinas, Mérida and Monagas)	4	Wenzel 1976
*Trichobiuspropinquus* (O)	*Anourageoffroyi*	Venezuela (Bolívar)	1	Wenzel 1976
*Trichobiussilvicolae* (M)	*Lophostomasilvicola* and *Phyllostomushastatus	Venezuela (Amazonas and Bolívar)	2	Wenzel 1976
*Trichobiussparsus* (M)	*Pteronotusparnellii* and *Natalustumidirostris (C)	Venezuela (Amazonas, Bolívar and Guárico)	10	Wenzel 1976
*Trichobiusstrictisternus*	Lophostomacarrikeri*	Venezuela (Amazonas)	1	Wenzel 1976
*Trichobiustiptoni* (M)	*Anouracaudifer*, *Carolliaperspicillata (C), *Desmodusrotundus (C), *Sturniraludovici (C) and *Platyrrhinushelleri (C)	Venezuela (Barinas, Bolívar, Carabobo and Yaracuy)	4	Wenzel 1976
*Trichobiustuttlei*	*Lampronycterisbrachyotis*	Venezuela (Amazonas)	1	Wenzel 1976
*Trichobiusuniformis* (O)	*Glossophagalongirotris*, *Glossophagasoricina*, *Artibeuslituratus (C), *Carolliaperspicillata (C) and *Platyrrhinushelleri (C)	Venezuela (Amazonas, Apure, Barinas, Bolívar, Guárico, Monagas, Trujillo and Yaracuy)	21	Wenzel 1976
*Trichobiusurodermae* (M)	*Urodermabilobatum*	Venezuela (Amazonas and Trujillo)	5	Wenzel 1976
*Trichobiusvampyrops* (O)	*Platyrrhinusvittatus*	Venezuela (Barinas)	1	Wenzel 1976
*Trichobioidesperspicillatus* (M)	*Phyllostomusdiscolor*	Colombia (Arauca, Meta) and Venezuela (Amazonas, Aragua, Barinas, Bolívar, Carabobo, Guárico, Monagas and Trujillo)	13	Wenzel 1976; Dick et al. 2016; López Rivera et al. 2022
*Xenotrichobiusnoctilionis*	*Noctilioalbiventris* and *Noctilioleporinus*	Venezuela (Amazonas and Apure)	2	Wenzel 1976
**Subfamily Streblinae**				
*Anastreblacaudiferae* (M)	*Anouracaudifer*	Venezuela (Amazonas, Barinas and Bolívar)	3	Wenzel 1976
*Anastreblamodestini* (M)	*Anourageoffroyi* and *Anoura* sp.	Venezuela (Amazonas, Barinas, Bolívar, Carabobo, Guárico, Mérida and Monagas)	12	Wenzel 1976
*Anastreblanycteridis* (O)	*Lonchophyllarobusta* and *Lonchophyllaorienticollina*	Colombia (Arauca) and Venezuela (Barinas)	2	Wenzel 1976; López Rivera et al. 2022
*Anastreblaspurrelli* (M)	*Lionycterisspurrelli* and *Ametridacenturio (C)	Venezuela (Amazonas and Bolivar)	7	Wenzel 1976
*Metalasmuspseudopterus* (Pl)	*Artibeusplanirostris*, *Artibeuslituratus, *Chirodermavillosum, *Myotisnigricans, *Peropteryxmacrotis, *Phyllostomushastatus and *Urodermamagnirostrum	Colombia (Meta) and Venezuela (Amazonas, Apure, Barinas, Bolivar, Guarico, Lara, Monagas, Trujillo and Yaracuy)	18	Wenzel 1976; Dick et al. 2016
*Metalasmus* sp.	*Sturniraludovici*	Venezuela (Barinas)	1	Wenzel 1976
*Paraeuctenodeslongipes* (M)	*Glossophagasoricina*, *Artibeuslituratus and *Nyctinomopslaticaudatus (C)	Venezuela (Amazonas, Bolivar and Yaracuy)	7	Wenzel 1976
*Paraeuctenodessimilis* (M)	*Carolliaperspicillata*	Venezuela (Bolivar)	2	Wenzel 1976
*Streblaaltmani* (O)	*Lonchorhinaaurita*, *Lonchorhinaorinocensis* and *Macrophyllummacrophyllum	Colombia (Meta) and Venezuela (Amazonas, Aragua, Apure, Barinas, Bolivar, Trujillo and Yaracuy)	15	Wenzel 1976; Dick et al. 2016
*Streblaalvarezi* (O)	*Micronycterismicrotis*, *Micronycterismegalotis*, *Carolliabrevicauda*, and *Lonchophyllathomasi*	Colombia (Meta) and Venezuela (Amazonas, Bolivar and Yaracuy)	5	Wenzel 1976; Dick et al. 2016
*Streblaasternalis* (M)	*Saccopteryxbilineata* and *Saccopteryx* sp.	Venezuela (Amazonas)	2	Wenzel 1976
*Streblachristinae* (M)	*Phyllodermastenops*, *Eumopsglaucinus (C) and *Urodermabilobatum (C)	Venezuela (Amazonas, Apure and Bolivar)	8	Wenzel 1976
*Streblachoropteri* (M)	*Chrotopterusauritus*	Venezuela (Amazonas and Bolivar)	4	Wenzel 1976
*Streblaconsocia* (O)	*Phyllostomuselongatus*, *Phyllostomushastatus*, *Desmodusrotundus (C), *Trachopscirrhosus (C) and *Platyrrhinushelleri (C)	Colombia (Meta) and Venezuela (Amazonas, Apure, Barinas, Bolivar, Carabobo, Monagas, Trujillo, Yaracuy)	21	Wenzel 1976; Dick et al. 2016
*Streblacormurae*	*Cormurabrevirostris*	Venezuela (Amazonas)	1	Wenzel 1976
*Streblacurvata* (O)	*Glossophagalongirostris*, *Glossophagasoricina*, *Carolliabrevicauda, *Carolliaperspicillata and *Noctilioalbiventris (C)	Venezuela (Amazonas, Apure, Barinas, Bolivar and Monagas)	12	Wenzel 1976
*Strebladiaemi* (M)	*Diaemusyoungii*	Colombia (Guainia) and Venezuela (Amazonas)	4	Wenzel 1976; Dick et al. 2016
*Strebladiphyllae*	*Diphyllaecaudata*	Colombia (Vaupes) and Venezuela (Aragua)	1	Wenzel 1976; Dick et al. 2016
*Streblagalindoi* (M)	*Tonatiabidens*	Venezuela (Amazonas, Apure and Bolivar)	4	Wenzel 1976
*Streblaguajiro* (O)	*Carolliabrevicauda* and *Carolliaperspicillata*	Colombia (Casanare, Meta and Vichada) and Venezuela (Amazonas, Apure, Aragua, Barinas, Bolívar, Guárico, Monagas, Trujillo and Yaracuy)	50	Wenzel 1976; Dick et al. 2016; Liévano et al. 2019
*Streblaharderi* (M)	*Anourageoffroyi* and *Anoura* sp.	Venezuela (Amazonas and Bolivar)	4	Wenzel 1976
*Streblahertigi* (O)	*Phyllostomusdiscolor* and *Phyllostomuselongatus (C)	Colombia (Arauca) and Venezuela (Amazonas, Barinas, Bolivar, Guarico, Monagas and Trujillo)	14	Wenzel 1976; López Rivera et al. 2022
*Streblakohlsi*	*Lophostomasilvicola*	Venezuela (Amazonas)	2	Wenzel 1976
*Streblamachadoi* (M)	*Micronycterisminuta*	Venezuela (Amazonas, Apure, Bolivar and Monagas)	4	Wenzel 1976
*Streblamatsoni* (M)	*Macrophyllummacrophyllum* and *Rhynchonycterisnaso (C)	Venezuela (Amazonas, Apure and Bolivar)	5	Wenzel 1976
*Streblamirabilis* (M)	*Trachopscirrhosus*, *Phyllostomushastatus C), *Phyllostomuselongatus (C), *Artibeusplanirostris, *Chrotopterusauritus (C) and *Diphyllaecaudata	Colombia (Meta) and Venezuela (Amazonas, Apure, Aragua, Bolivar, Guarico and Yaracuy)	19	Wenzel 1976; Dick et al. 2016
*Streblaobtusa* (M)	*Trinycterisnicefori* and *Phyllostomuselongatus (C)	Venezuela (Amazonas and Bolivar)	5	Wenzel 1976
*Streblaparamirabilis* (Pl)	*Artibeusplanirostris*, *Platyrrhinusaurarius* and *Anourageoffroyi (C)	Colombia (Meta) and Venezuela (Amazonas and Bolivar)	5	Wenzel 1976; Dick et al. 2016
*Streblaproxima* (O)	*Peropteryxmacrotis* and *Peropteryxtrinitatis*	Venezuela (Amazonas and Yaracuy)	2	Wenzel 1976
*Streblatonatiae* (O)	*Lophostomabrasiliense*, *Tonatiasaurophila, *Lophostomacarrikeri (C) and *Sturniralilium “(probably *S.giannae*)” (C)	Colombia (Casanare) and Venezuela (Amazonas, Apure, Bolivar, Monagas, Trujillo and Yaracuy)	9	Wenzel 1976; Liévano-Romero et al. 2019
*Streblawiedemanni* (M)	*Desmodusrotundus*	Colombia (Meta) and Venezuela (Amazonas, Apure, Barinas, Bolivar, Guárico, Lara, Monagas, Trujillo and Yaracuy)	28	Wenzel 1976; Dick et al. 2016
**Family Nycteribiidae**				
*Basiliaanceps*	Unidentified host	Venezuela (Bolivar and Amazonas)	2	Graciolli et al. 2007
*Basiliabequaerti*	Unidentified host	Venezuela (Amazonas	1	Guimarães 1972
*Basiliaconstricta*	Unidentified host	Venezuela (Mérida)	1	Guimarães 1972
*Basiliadunni*	Unidentified host	Venezuela (Amazonas)	1	Graciolli et al. 2007
*Basiliadubia* (M)	*Myotisalbescens* and *Saccopteryxbilineata (C)	Venezuela (Amazonas and Apure)	3	Guimarães 1972
*Basiliaferrisi* (O)	*Myotisalbescens*, *Myotishandleyi*, *Myotisnigricans*, *Myotisriparius*, *Molossuspretiosus (C), *Desmodusrotundus, *Noctilioalbiventris (C), *Phyllostomuselongatus (C) and *Platyrrhinushelleri* (C)	Colombia (Arauca and Meta) and Venezuela (Amazonas, Apure, Aragua, Bolivar and Monagas)	8	Guimarães 1972; Graciolli et al. 2007; López Rivera et al. 2022
*Basiliajuquiensis* (O)	*Myotisriparius*	Venezuela (Apure)	1	Guimarães 1972
*Basiliaortizi* (O)	*Eptesicusbrasiliensis*, *Eptesicusfurinalis*, *Eptesicusorinocensis* and *Myotisriparius	Colombia (Arauca), Venezuela (Amazonas, Bolivar and Monagas)	8	Guimarães 1972 López Rivera et al. 2022
*Basiliatiptoni* (M)	*Gardnerycteriscrenulatum*	Venezuela (Apure and Trujillo)	2	Guimarães 1972
*Basiliatyphlops*	*Myotisoxyotus*	Venezuela (Bolivar)	1	Guimarães 1972
*Basiliatuttlei*	*Myotisnigricans*	Venezuela (Amazonas)	1	Guimarães 1972
*Basiliawenzeli*	*Eptesicusfuscus* and *Lonchorhinaaurita*	Venezuela (Aragua)	1	Guimarães 1972

We also included additional and noteworthy records of ectoparasitic bat flies collected from bats captured using four mist nets (12 × 2.5 m; with 36 mm mesh size) during November 2021 (8 days) in two localities of the Orinoquia Region. For this we performed field work in two localities of the municipality of Arauca in the Department of Arauca (Fig. 1), Colombia (1: Vereda El Socorro, Finca Los Trompillos, and 2: Vereda El Socorro, Finca Marsella; Table 2). The Department of Arauca is located in the Orinoquia Region bordering to the north and east with Venezuela, and occupies an area of 23,818 km^2^ dominated by herbaceous plains and chaparral (Mosquera Guerra et al. 2019). The region shows a typical savanna climate with a well-defined wet season between June and July and a very dry season between December and April (López Rivera et al. 2022). The mist nets were placed randomly in both localities and operated between 18:00 and 22:30 hours. We examined each captured bat to search for ectoparasites by placing a clean white blanket on them and in some cases moistening the fur with 70% alcohol, to facilitate handling. We collected the flies and other ectoparasites using entomological tweezers and stored in Eppendorf tubes with 96% ethanol. The flies were collected and handled under the permission granted by the Autoridad Nacional de Licencias Ambientales (ANLA) to the Universidad de Caldas (Resolution 02497 of 31 December 2018) and by approval of the bioethics committee of the Facultad de Ciencias Exactas y Naturales of the Universidad de Caldas (2 June 2017). Bats and ectoparasites were deposited at the Mammals (M) and Ectoparasites (Ec) collections of the MHN-UCa. Of these records, we calculated the general prevalence for host species defined as the number of individuals of a host species infected with a particular parasite species divided by the number of hosts examined (Margolis et al. 1982).

**Table 2.** Ectoparasitic flies collected on bats during 2021 in the Department of Arauca, Colombia. * New records of bat fly species for Colombia. ** New association between bat flies and bats.

Taxon	No individuals	Host	Prevalence	Locality	Coordinates	Voucher
**Streblidae**						
*Mastopteraguimaraesi*	7♀ and 5♂	*Phyllostomushastatus* (1♂)	1.00	1	06°46'47"N, 70°42'59.3"W	MHN-UCa-Ec 555
*Noctiliostreblamaai*	12♀ and 9♂	*Noctilioalbiventris* (1♀ and 2♂)	0.26	1	06°46'46.4"N, 70°43'00"W; 06°46'46.7"N, 70°43'02.1"W	MHN-UCa-Ec 555, 561 and 562
	37♀ and 27♂	*Noctilioalbiventris* (♀ and ♂)	0.26	2	06°46'43.2"N, 70°43'36.1"W	MHN-UCa-Ec 565, 568, 570–573, 580, 583, 586 and 588
*Paradyschiriaparvuloides*	1♀	*Cynomopsplanirostris* (1♀)**	0.1	1	06°46'43.2"N, 70°43'36.1"W	MHN-UCa-Ec 509
	27♀ and 15♂	*Noctilioalbiventris* (1♀ and 3♂)	0.32	1	06°46'46.4"N, 70°43'00"W; 06°46'46.7"N, 70°43'02.1"W	MHN-UCa-Ec 557, 559, 560 and 563
	39♀ and 32♂	*Noctilioalbiventris* (♀ and ♂)	0.32	2	06°46'43.2"N, 70°43'36.1"W	MHN-UCa-Ec 566, 567, 569, 572, 574–579, 581, 582, 585 and 589
Trichobiusjubatus*	1♂	*Molossusrufus* (♂)	0.05	2	06°46'43.2"N, 70°43'36.1"W	MHN-UCa-Ec 564
*Trichobiusparasiticus*	1♂	*Desmodusrotundus* (1♂)	1.00	1	06°46'46.3"N, 70°42'59.2"W	
**Nycteribiidae**						
*Basiliaferrisi*	6♀ and 5♂	*Myotishandleyi* (1♀ and 1♂)	0.25	1	06°46'47"N, 70°42'59.3"W	MHN-UCa-Ec 552

For the taxonomic identification of the captured bats, we took morphometric measurements including the total length, tail length, forearm length, ear length and foot length (Nagorsen and Peterson 1980) and used taxonomic keys (e.g., Gardner 2008). For the identification of the specimens of Streblidae and Nycteribiidae, we used the dichotomous keys of Wenzel et al. (1966), Wenzel (1976), Guerrero (1994, 1995, 1998), Autino et al. (1999), and Alcantara et al. (2019) using morphological features to distinguish between different species (Wenzel 1976; Autino et al. 1999). These traits include the shape and size of the body, wing, mesonotum, head, eyes and female and male reproductive structures. Identifications were done with the help of a stereomicroscope and compared with additional specimens deposited in the MHN-UCa-Ec collection.

### ﻿Bat-ectoparasite network structure and complex network metrics

We used the new records reported in the present study and the records collected in the literature to build bipartite interaction networks for the Streblidae and Nycteribiidae of the Orinoquia. In the networks, bat and ectoparasite species are represented by nodes and interacting species are linked by lines, with the width of the line proportional to the frequency of each interaction. We created a net that includes all raw records, as well as other nets that exclude records that could be the product of incidental transfer or contamination. In addition, we carried out an analysis of the Streblidae species present in the interaction networks, classifying them into the three ecomorphological groups proposed by Dick (2005): wing crawler, fur runners, and fur swimmers. This allowed us to infer possible niche partitioning between host species across the networks.

To evaluate the properties of the network we used the index of specialization by communities (*H_2_*’), the quantitative modularity QuanBiMo (*Q*), the connectance (*C*), and the nestedness (*wNODF*) (Dormann et al. 2009; Fortuna et al. 2010; Mello et al. 2016). The standardized two-dimensional entropy index (*H_2_*’) measures both the degree of niche complementarity among species and the specialization at species level (Blüthgen et al. 2006). This index varies from 0 (unspecialized network) to 1 (perfectly specialized network). We calculated Quantitative Modularity (QuanBiMo) that allows determining the existence of sets or groupings within the complete network, that is, when there are species that interact more closely, forming modules (Fortuna et al. 2010). The modularity ranges from 0 (non-modular) to 100 (fully modular) and were estimated using the algorithm QuanBiMo (Dormann and Strauss 2014). The C index represents the number of interactions or links observed in the network, between bats and their ectoparasitic flies considering the total number of potential interactions. It takes values from 0 to 1 where 0 indicates that there are no connections and 1 which denotes that most of the nodes in the network interact with each other (Blüthgen et al. 2006). Then we calculated the Weighted Nestedness (*wNODF*) ranging from 0 (non-nested) to 100 (fully nested), to measure how strongly species interactions of seldom connected species were nested within those of highly connected species (Almeida-Neto and Ulrich 2011).

Also, we assessed the role of bat and fly species using one centrality metric. The degree centrality (*DC*) measured the number of interactions of a given species, reflecting its degree of generalization versus specialization and the (González et al. 2010). All calculations were carried out using the R software (R Core Team 2022) and the vegan (diversity analysis), iNEXT (interaction accumulation curve), network and bipartite (interaction networks and metrics) packages (Butts 2008; Dormann et al. 2008; Hsieh et al. 2016; Oksanen et al. 2020).

## ﻿Results

### ﻿Richness of bats and ectoparasite flies in the Orinoco Region

Our review showed 1046 records of 129 species of ectoparasitic flies associated with 102 species of bats (Table 1) in the Orinoquia Region (Suppl. material 1: table S1), and seven species (*Basiliaanceps*, *Basiliabequaerti*, *Basiliaconstricta*, *Basiliadunni*, *Noctiliostrebladubia*, *Trichobiusdybasi* and *Trichobiusfurmani*) in which the associated host were not recorded (Table 1, Suppl. material 1: table S2). The flies were mainly associated with bats of the families Phyllostomidae (69 species, ~ 79% of the records), followed by Vespertilionidae (10 species), Molossidae (seven species), Emballonuridae (six species), Mormoopidae (six species), Noctilionidae (two species), Furipteridae (one species), and Natalidae (one species) (Table 1). Species of Nycteribiidae were found associated mainly with species of Vespertilionidae (27 records of seven species of flies associated with nine species of bats), followed by Phyllostomidae (six records of three species of flies associated with five species of bats) (Table 1). 119 species of Streblidae were reported associated mainly with species of Phyllostomidae (821 records of 97 species of bat flies associated with 68 species of bats) followed by Mormoopidae (89 records of 11 species of bat flies associated with six species of bats) (Table 1).

The evaluation of the quality of the associations between flies and hosts showed that 9.8% of them could be the result of incidental transfers or contamination (Table 1). For example, reported associations of *Trichobiuscaecus* were found with 11 bat species, of which nine are considered non-primary associations and might represent incidental transfers or contamination. Similarly, reported associations of *Aspidopterafalcata* with nine bat species were recorded, of which six were identified as possible contaminations. We also detected unresolved non-primary associations where a significant number of individuals (> 5% of records) were found in the absence of the primary hosts. For example: *Mastopteraminuta* is primarily associated with *Lophostomasilvicola* but Liévano-Romero et al. (2019) reported 16 individuals of this species parasitizing *Tonatiamaresi* in Casanare, Colombia. Furthermore, *Megistopodaproxima* is primarily associated with *Sturnira* species, but López Rivera et al. (2022) found three individuals parasitizing *Noctilioalbiventris* and seven individuals of *Noctiliostreblamaai* on a *Sturniragiannae* in the absence of the primary host (*N.albiventris*) in Arauca, Colombia (Table 1). Distinguishing non-primary associations due to contamination among ecological associations were also challenging for species complexes, given their taxonomic complexity. For instance, *Mastopteraminuta* s.l. were recorded associated with eight bat species belonging to four genera (*Anoura*, *Artibeus*, *Lophostoma*, *Phyllostomus*). *Paratrichobiussalvini* s.l. were reported associated with five bat species from three genera (*Chiroderma*, *Platyrrhinus*, and *Vampyriscus*). *Neotrichobiusdelicatus* s.l. were found associated with two bat species from different genera (*Dermanuracinerea* and *Rhynophyllapumilio*). Furthermore, the specificity of other ectoparasitic fly species could not be determined due to limited or unique records, such as *Neotrichobiusectophyllae*, *Neotrichobiusstenopterus*, *Streblacormurae*, *Trichobiuspallidus*, and *Trichobiustuttlei* (Table 1). Regarding the morphological confirmation of some non-primary associations previously reported for the Department of Arauca, Colombia, we reidentified samples of *Trichobiusmendezi* found in association with *Molossusmolossus* and *Molossuspretiosus* as *Trichobiusjubatus*. Similarly, *T.mendezi* found in non-primary association with *Phyllostomushastatus* were morphologically reidentified as *Trichobiuslongipes*.

### ﻿New records for Colombia

**Figure 2.**
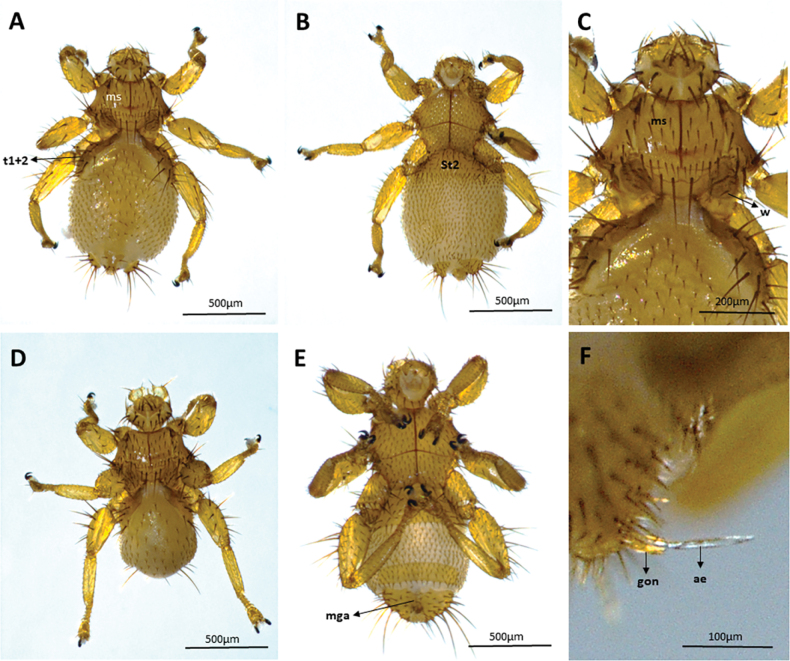
Micrographs of *Mastopteraguimaraesi*: female **A** dorsal and **B** ventral views (minute specie, body 0.73–1.4 mm long, with short legs) **C** lateral lobes of tergum 1+2, these longer and heavier, dorsal view; male **D** dorsal and **E** ventral views **F** male genital apparatus, lateral view. Abbreviations: ae: aedeagus; gon: gonopods; mga: male genital apparatus; ms: mesonoto; St2: Sternum 2; t1+2: tergum l + 2, w: wings.

**Figure 3.**
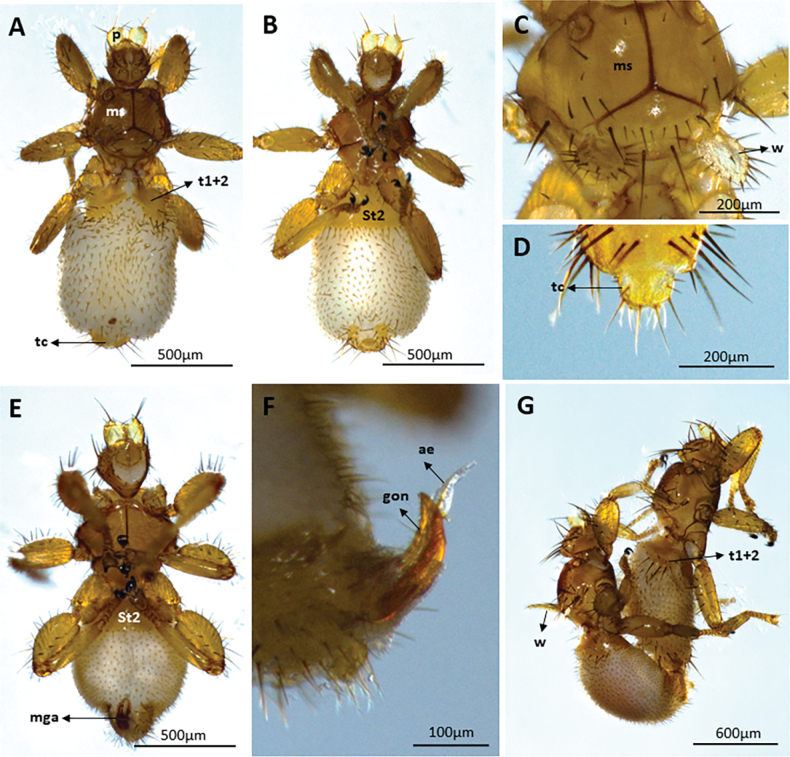
Micrographs of *Noctiliostreblamaai*: female **A** dorsal and **B** ventral views (body 3.0–3.2 mm long) **C** median and transverse mesonotal sutures united to form an inverted Y and median wing vein usually with 1 or 2 setae (arrowed), dorsal view **D** terminal cone, female abdomen, ventral view **E** male, ventral views **F** male genital apparatus, lateral view and **G** male and female copulating. Abbreviations: ae: aedeagus; gon: gonopods; mga: male genital apparatus; ms: mesonoto; St2: Sternum 2; sst: surstylus; t1+2: tergum l + 2; tc: termina cone.

**Figure 4.**
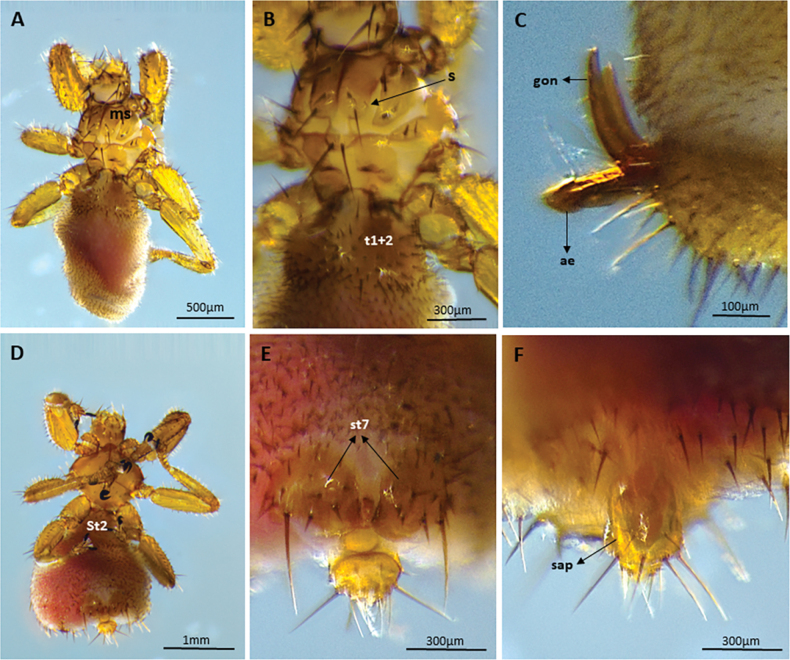
Micrographs of *Paradyschiriaparvuloides*: male **A** dorsal view (body 2.5–3.0 mm long) **B** mesonotum usually with a short seta on each side anterior and lateral to the long posterior macroseta (arrow), dorsal view **C** male genital apparatus, lateral view; female **D** ventral view **E** seventh sternites each with 3 or -4, rarely 2, spine-like setae on distal margin (arrows), ventral view **F** supra-anal plate as wide as long, the basal (anterior) margin roundly angulate, dorsal view. Abbreviations: ae: aedeagus; gon: gonopods; ms: mesonoto; sap: supra-anal plate; St2: Sternum 2; St7: Sternum 7; sst: surstylus; t1+2: tergum l + 2.

**Figure 5.**
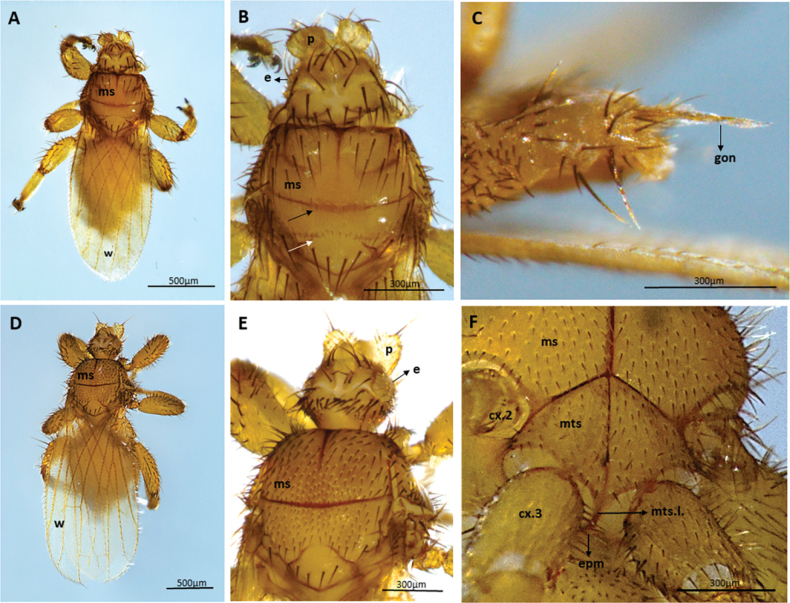
**A–C** Micrographs of *Trichobiusparasiticus* male: **A** dorsal view (body 5.4 mm long) **B** head (latero vertices and occipital lobes well defined) and mesonotum (prescutum with very short discal setae immediately in front of the transverse suture (black arrow) and scutum posteriorly with an irregular W-shaped row of short setae (white arrow)), dorsal view **C** gonopods, lateral view **D–F** micrographs of *Trichobiusjubatus* male: **D** dorsal views (body 3.8 mm long) **E** head (occipital lobes of head densely setose, eyes multifaceted) and mesonotum (mesonotum essentially setose throughout, and median and transverse sutures not united), dorsal view **F** metasternal lobe united with metepimeron (arrows), ventral view. Abbreviations: cx.2: mesocoxa; cx.3: metacoxa; e: eyes; epm: meteprmeron; gon: gonopods; mga: male genital apparatus; ms: mesonoto; mts: metasternum; mts.l.: metasternal lobe; St2: Sternum 2; t1+2: tergum l + 2; w: wings.

**Figure 6.**
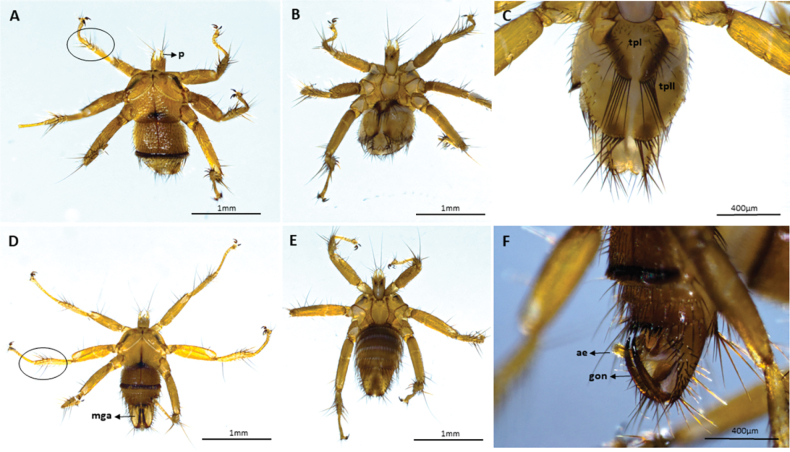
Micrographs of *Basiliaferrisi*: female **A** ventral and **B** dorsal views: body 1.8–2.0 mm long, tibiae with three rows of setae (circle) **C** female with: tergal plate II transformed into two elongate lobes with short and long setae or with posterior margin arcuate, dorsal view; male **D** ventral and **E** dorsal views **F** male genital apparatus, lateral view. Abbreviations: ae: aedeagus; gon: gonopods; mga: male genital apparatus; tpI: tergal plate I and tpII: tergal plate II. Circles **A, D** indicate tibiae with three rows of setae.

We captured 141 bats of 16 species (*Carolliabrevicauda*, *Cynomopsmilleri*, *Cynomopsplanirostris*, *Desmodusrotundus*, *Eptesicusorinocensis*, *Eumopsglaucinus*, *Eumopsnanus*, *Molossopstemminckii*, *Molossuscoibensis*, *M.molossus*, *M.pretiosus*, *Molossusrufus*, *Myotishandleyi*, *N.albiventris*, *P.hastatus* and *Urodermamagnirostrum*) of which 24 individuals of six bat species had ectoparasitic flies (*C.planirostris*, *D.rotundus*, *M.handleyi*, *M.rufus*, *N.albiventris*, and *P.hastatus*). In total, we obtained 223 flies belonging to five species of Streblidae: *Mastopteraguimaraesi* (Fig. 2), *N.maai* (Fig. 3), *Paradyschiriaparvuloides* (Fig. 4), *Trichobiusjubatus* (Fig. 5A–C), and *T.parasiticus* (Fig. 5D, F), and one species of Nycteribiidae: *Basiliaferrisi* (Fig. 6). Our study reports for the first time the presence *T.jubatus* in Colombia previously reported in Venezuela (Table 2). The association between *Paraduschiriaparvuloides* and *C.planirostris*, although novel, could be related to possible contamination, since only one individual was collected in the presence of the primary host *N.albiventris* (see Table 2).

### ﻿Bat-ectoparasite network structure and complex network metrics

The Streblidae-bat interaction network for the Orinoquia was made up of 121 species of ectoparasitic flies and 91 species of bats (Suppl. material 2). The quantitative modularity QuanBiMo and specialization calculated for the interaction network was high (*H_2_*’ = 0.94 and *Q* = 0.85, respectively), which indicates a high niche (bats) differentiation in the network. We observed low connectance (*C* = 0.02) and nestedness (*wNODF* = 2.39). The degree of centrality (*DC*) shows us 66 highly specialized bat flies interacting with a single bat species (Suppl. material 1: table S3), and ten relatively generalist species interacting with more than four species of bats: *Aspidopteradelatorrei*, *Exastinionclovisi*, *M.proxima*, *Nycterophiliacoxata*, *Nycterophiliaparnelli*, *Streblaalvarezi*, *Streblacurvata*, *Trichobiusdugesii*, *Trichobiusjohnsonae* and *Trichobiusparasiticus* (*DC* = 4) and one highly generalist species parasitizing eight bat species: *Metalasmuspseudopterus* (*DC* = 8). In addition, as expected, the species complexes also proved to be the most general, with associations encompassing up to five host species: *M.minuta* s.l., *N.delicatus* s.l., *P.longicrus* s.l., and *Paratrichobiussalvini* s.l. (Suppl. material 1: table S3). We also observed that 39 of the 91 bat species were parasitized by a single fly species (Suppl. material 1: table S4) and 11 bat species were parasitized by more than five bat fly species: *Anourageoffroyi*, *Artibeuslituratus*, *Lophostomasilvicola*, *Noctilioleporinus* and *Pteronotusparnellii* (probably *P.fuscus*) (*DC* = 5), *C.brevicauda*, *N.albiventris*, and *P.hastatus* (*DC* = 7), *Phyllostomuselongatus* (*DC* = 9), *Artibeusplanirostris* (*DC* = 10), and *Carolliaperspicillata* (*DC* = 11) (Suppl. material 1: table S4). These bat species are key hosts, as they act as connectors between the different bat fly species that make up the web. This means that these species play a fundamental role in the interaction between different species of ectoparasites, therefore, their presence and characteristics can be determinant for the survival and dispersal of ectoparasites in the network.

**Figure 7.**
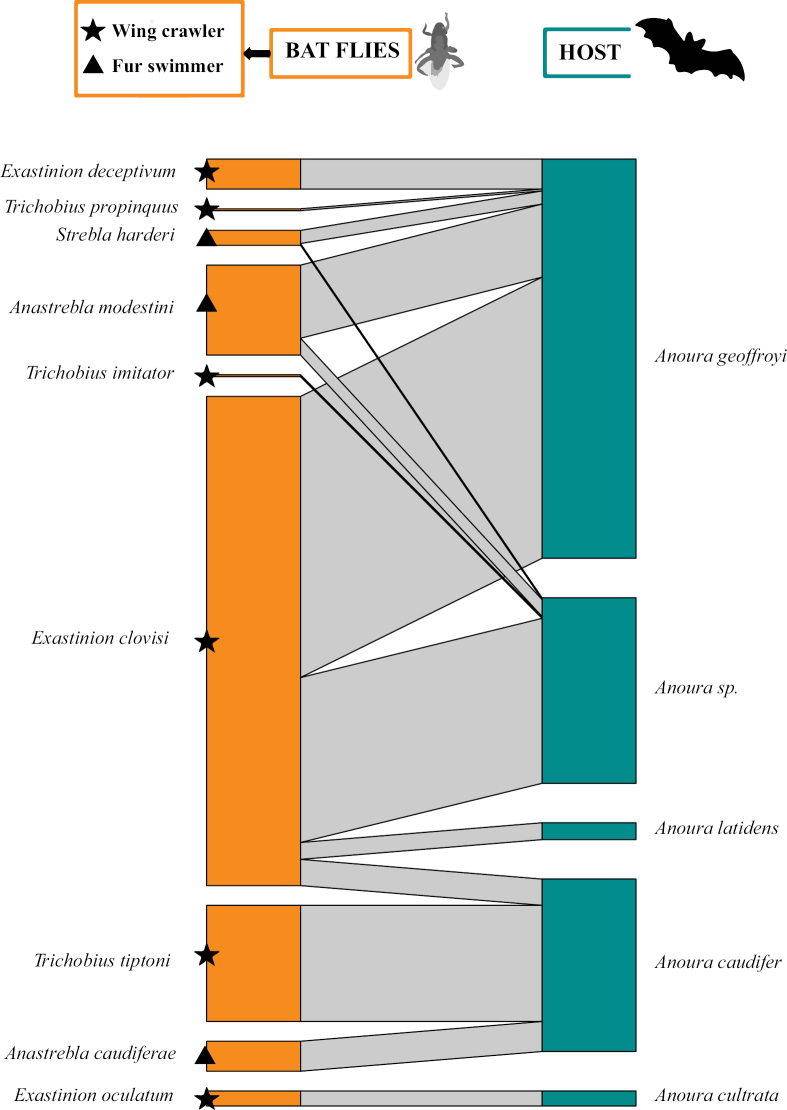
Bipartite bat-fly quantitative network (Module 1 - Streblidae). The size of the bar on the left (orange) represents the abundance (number of individuals) of bat flies per species observed and the size of the bar on the right (green) represents the abundance of bats for which the bat fly sample was obtained. The width of the black lines/bars indicates the frequency of interactions.

The analysis of the interaction network between Streblidae and bats in the Orinoquia revealed a high modularity and specialization and three different modules. The first module or group was composed of nine species of flies belonging to four genera (*Anastrebla*, *Exastinion*, *Trichobius*, and *Strebla*) exclusive to bats of the genus *Anoura* (Fig. 7). In this group, *E.clovisi* were observed to be the most generalist species, associated with three *Anoura* species, but showing a stronger association with *Anourageoffroyi*. *Anourageoffroyi* was the bat species with the highest number of associated fly species (*DC* = 5). In module 1, two ecomorphological groups were mainly found: the “wing crawler” (represented by *Exastinion* and *Trichobius*) and the “fur swimmer” (represented by *Anastrebla* and *Strebla*). In general, the species of both groups were associated with the same hosts, indicating the possibility of a niche partition within the host bat species (Fig. 7).

**Figure 8.**
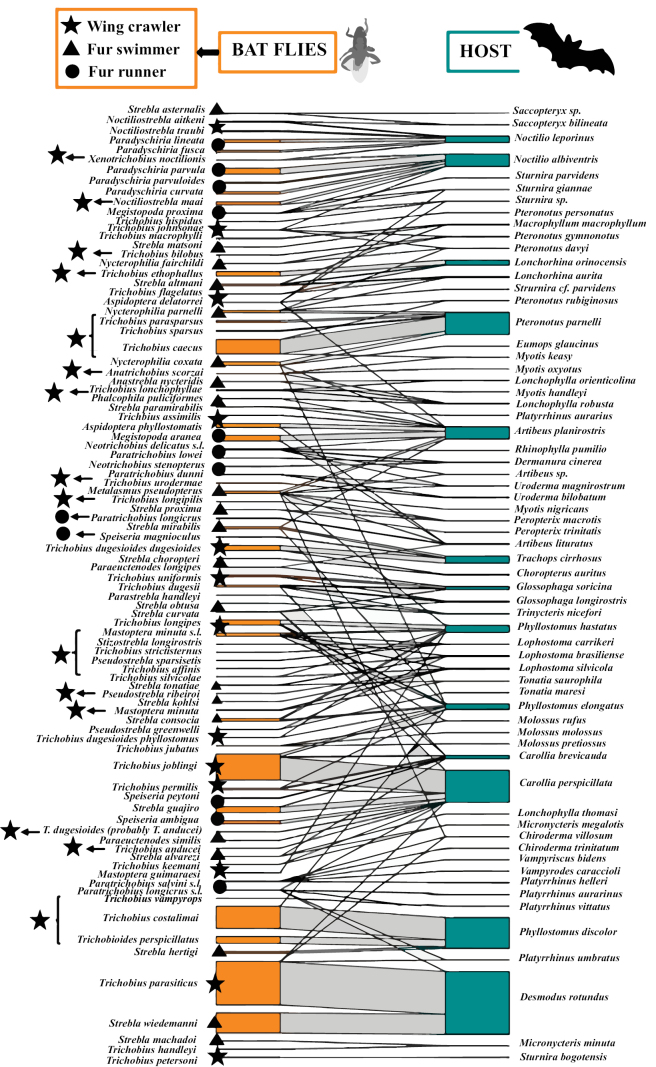
Quantitative bipartite bat-fly network (Module 2 - Streblidae). The size of the left bar (orange) represents the abundance (number of individuals) of bat flies per observed species and the size of the right bar (green) represents the abundance of bats for which the sample was obtained. Bat fly. The width of the black lines/bars indicates the frequency of interactions.

The second module (the largest group) was composed of 91 species of flies of the three subfamilies (Nycterophiliinae, Strebliinae and Trichobiinae) associated with 64 species of bats of the families Emballonuridae, Mormoopidae, Molossidae, Noctilionidae, Phyllostomidae, and Vespertilionidae, (Fig. 8). In this group, highly interconnected hosts are highlighted, hosting several species of ectoparasitic flies. Among them there are two species of Noctilionidae (*N.leporinus* and *N.albiventris*), one Mormoopidae (*Pteronotusparnellii*; probably *P.fuscus*), and seven of Phyllostomidae (*A.planirostris*, *Trachopscirrhosus*, *Phyllostomuselongatus*, *P.hastatus*, *Phyllostomusdiscolor*, *C.brevicauda*, and *C.perspicillata*). Additionally, we confirmed primary associations supported by their abundances, such as *Paradyschiriaparvula* with *N.albiventris*, *T.caecus* with *P.parnellii* (probably *P.fuscus*), *Trichobiusjoblingi* with *C.perspicillata*, *Trichobiuscostalimai* with *Phyllostomusdiscolor*, and *T.parasiticus* and *Streblawiedemanni* with *D.rotundus*. For the second module the three ecomorphological groups were identified. Wing crawlers were the most representative, comprising ~ 57% of the fly species, followed by fur swimmers, representing ~ 27.5% of the fly species, and finally, in smaller proportion, fur runners (Fig. 8). In general, in this module, species from different ecomorphological groups were associated with the same host species. In some cases, only two ecomorphological groups were present, while for more generalist host species such as *A.planirostris* and *C.perspicillata*, all three ecomorphological groups were found together. Although the majority of the studies analyzed in this work do not specify which fly specimens were collected on the same hosts, these associations may indicate niche partitioning within the same host bat species (Fig. 8).

**Figure 9.**
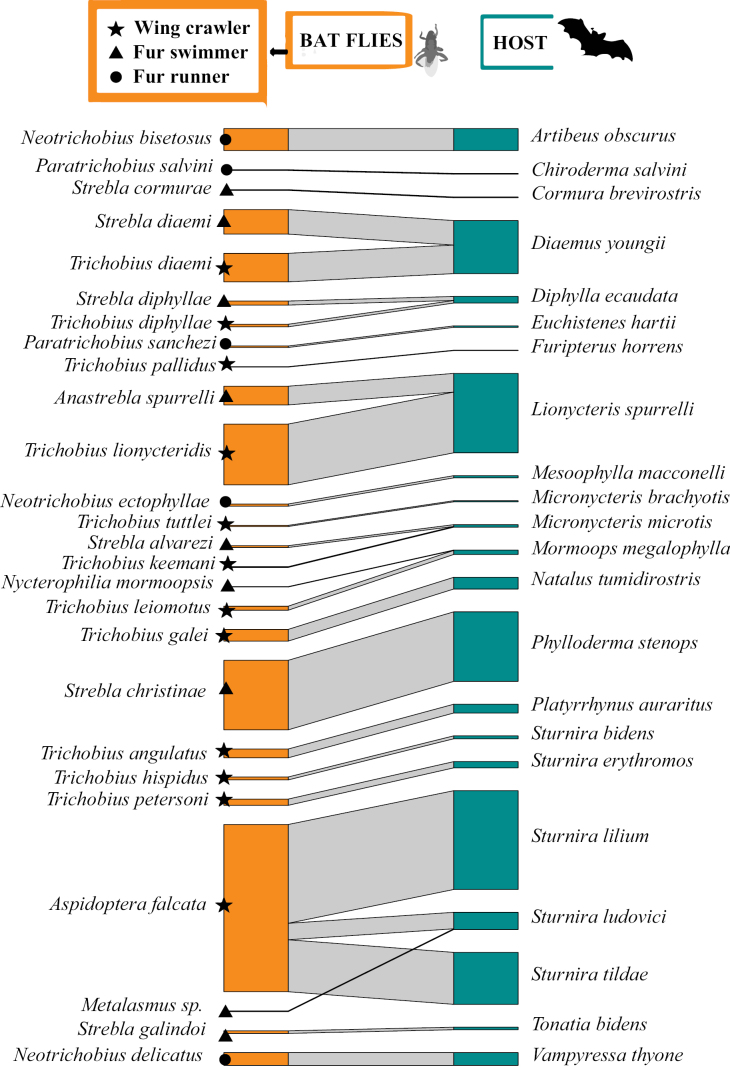
Quantitative bipartite bat-fly network (Module 3 - Streblidae). The size of the left bar (orange) represents the abundance (number of individuals) of bat flies per observed species and the size of the right bar (green) represents the abundance of bats for which the sample was obtained. The width of the black lines/bars indicates the frequency of interactions.

The third module of Streblidae interaction network is represented by the most specific species or unique associations. It consists of 25 species of bat flies and 21 species of bats of the families Emballonuridae, Furipteridae, Mormoopidae, Natalidae, and Phyllostomidae (Fig. 9). We confirmed primary associations based on the abundance of records between *Neotrichobiusbisetosus* and *Artibeusobscurus*; *Strebladiaemi* and *Trichobiusdiaemi* with *Diaemusyoungii*; *Anastreblaspurrelli* and *Trichobiuslionycteridis* with *Lionycterisspurrelli*; *Streblachristinae* with *Phyllodermastenops*, and finally, *A.falcata* with three species of *Sturnira* (S.cf.lilum, *S.ludovici*, and *S.tildae*). Like modules 1 and 2 of this network, the incidence of different ecomorphological types within the same host bat species suggests niche partitioning pattern across the entire network (Fig. 9).

**Figure 10.**
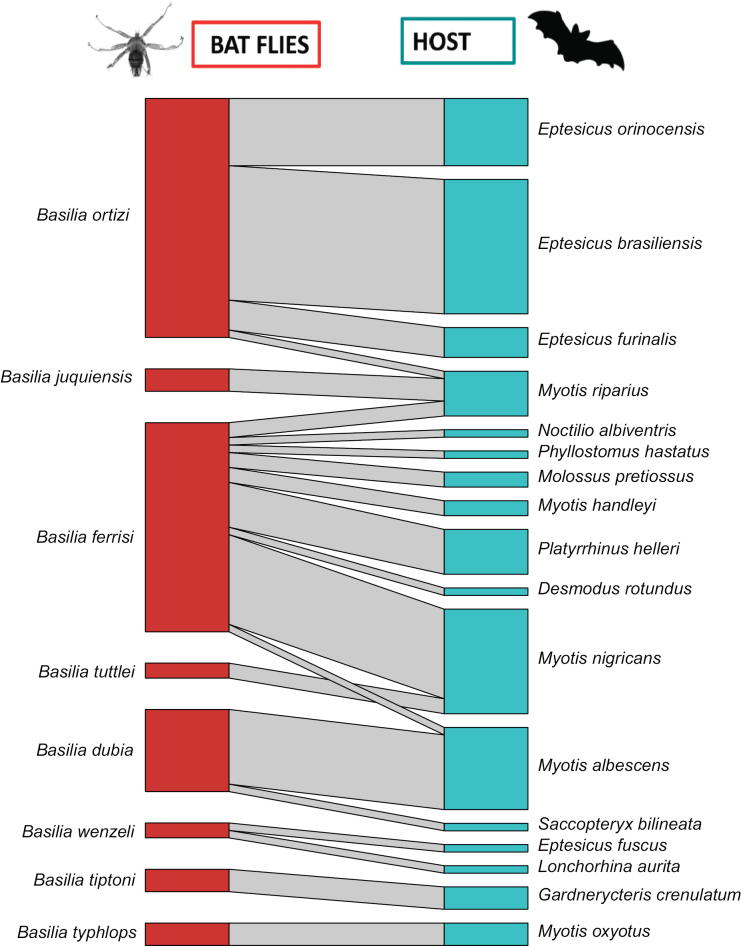
Quantitative bipartite bat-fly network (Nycteribiidae). The size of the left bar (red) represents the abundance (number of individuals) of bat flies per observed species and the size of the right bar (blue) represents the abundance of bats for which the sample was obtained. The width of the black lines/bars indicates the frequency of interactions.

For Nycteribiidae, the interaction network was composed of eight species of flies of the genus *Basilia* and 17 species of bats mainly of the families Vespertilionidae (nine species) and Phyllostomidae (five species), with additional records of unique species of the families Emballonuridae (*Saccopteryxbilineata*), Molossidae (*M.pretiosus*), and Noctilionidae (*N.albiventris*) (Fig. 10). The quantitative modularity QuanBiMo for the interaction network was also high but lower compared to the findings for Streblidae (*Q* = 0.67). The specialization (*H_2_*’ = 0.88) was higher, indicating a high niche differentiation among the bats in the network. Furthermore, we observed low connectivity (*C* = 0.15); however, nestedness (*wNODF* = 16.31) was higher compared to Streblidae. The degree of centrality (*DC*) was notable for *B.ferrisi* (*DC* = 9) and *Basiliaortizi* (*DC* = 4), which were found parasitizing multiple species of host bats. Particularly, *Basiliaortizi* showed strong interaction with the genus *Eptesicus*, while *Basiliaferrisi* exhibited strong interaction with *Myotisnigricans*.

## ﻿Discussion

Our study highlights the high diversity of bat flies that inhabit the Orinoco Region in northern South America. In other neotropical countries such as Brazil, Paraguay, and Panama, several studies have found a directly proportional relationship between the richness of Streblidae flies and bats (Dick and Gettinger 2005; Graciolli and Bianconi 2007). Considering that Colombia and Venezuela, are among the countries with the greatest diversity of bats in the Neotropics (with 217 and 172 species, respectively; Delgado-Jaramillo et al. 2016; Ramírez-Chaves et al. 2021), the finding of 136 species of bat flies in the Orinoquia Region was expected and will increase with additional studies in poorly sampled areas the Andean foothills of the Orinoco basin were endemic bat species such as *Vampyressavoragine* inhabit (Morales-Martínez et al. 2021). Besides the elevated number of the species documented in the Orinoquia, there are still gaps of information about these flies confirming the need for greater sampling efforts, particularly in some states/departments and biomes such as Portuguesa, Cojedes and Delta Amaruco in Venezuela and Guaviare in Colombia. The richness of bat flies in Colombia and Venezuela is also higher and surpasses the number of species documented in countries like Brazil, which despite having the largest area in South America (48%), has 181 species of bats (Garbino et al. 2022) and > 100 species of Streblidae and 26 of Nycteribiidae (da Silva et al. 2023).

Although the high specialization of some species of Streblidae has been controversial and some researchers have previously described them as mostly host-nonspecific (Dittmar et al. 2006), the evaluation of the quality of the non-primary associations reported in the literature for Orinoquia showed that several of them (~ 10% of the records) were probably due to accidental transfers or contamination of the samples during collection. These results support more recent studies suggesting that most species are host-specific (de Vasconcelos et al. 2016; Estrada-Villegas et al. 2018), a change attributable to methodological improvements in sample collection and taxonomic updates of flies and hosts. In addition, we have found controversial non-primary associations that could not be classified as possible cases of contamination. Some of these associations occurred in congeneric species, as is the case of the reports of *M.minuta* (primary host: *L.silvicola*) on *T.maresi* in Casanare, Colombia (Liévano-Romero et al. 2019). Considering that some unusual interaction occurred in non-congeneric species of the primary hosts, explaining the reasons behind is complicated. This is the case of *N.albiventris* parasitized by *M.proxima* (primary host: *Sturnira* species), and *S.giannae* parasitized by *N.maai* (primary host: *N.albiventris*) (López Rivera et al. 2022). Furthermore, the nature of the specificity in some non-primary associations for certain fly species complexes could not be determined (Table 1). In this sense, we suggested that the determination of the true specificity of ectoparasitic flies will only be achieved when: 1) large samples of hosts and parasites are available, 2) sampling protocols strictly control cross-contamination between hosts, and 3) association cases are evaluated statistically (Dick 2005).

Although most studies on the interaction between bats and ectoparasites have been descriptive, these are crucial for understanding host-parasite dynamics in different environments (Patterson et al. 2007; Fagundes et al. 2017; Salinas-Ramos et al. 2018). Previous studies focused on interactions networks only covered few localities in the Colombian Orinoquia (López Rivera et al. 2022) or were limited to enumerating species and abundances in specific locations in Colombia (departments of Arauca, Casanare, and Meta) and Venezuela (states of Anzoátegui, Apure, Aragua, Barinas, Carabobo, Guárico, Lara, Mérida, Monagas, Trujillo and Yaracuy) (Wenzel and Tipton 1966; Wenzel 1976; Marinkelle and Grose 1981; Dick et al. 2016; Liévano-Romero et al. 2019; López Rivera et al. 2022). Considering that, our study represents the first attempt to define the ecological interactions between ectoparasitic flies and their host bats in the Orinoquia Region of Colombia and Venezuela. However, previously reported modularity and specialization values for the interaction networks in the Department of Arauca, in Colombia (*Q* = 0.61 and 0.69; *H_2_*’=0.78 and 0.91) (López Rivera et al. 2022), were similar to the values found for Nycteribiidae in the network of the entire Orinoquia (*Q* = 0.67 and *H_2_*’=0.88), but lower compared to the values found in the Streblidae network of the entire Orinoquia (*Q* = 0.85 and *H_2_*’=0.94). These findings match suggestions in the literature where the size of the network can influence the modularity values, being higher for larger networks (Júnior et al. 2020). This can be explained by the highly specialized nature of the interaction between bats and bat flies (Dick and Patterson 2007), where most fly species are associated with a single host (monoxenic), and even species that parasitize multiple bat species (oligoxenic and polyxenic) are restricted to phylogenetically close hosts, leading to the formation of groups with a similar composition of ectoparasitic fly species (Falcão 2015; López Rivera et al. 2022).

Ectoparasites typically display some host specificity, implying that some ectoparasitic fly species are adapted to parasitize only one or a few bat species (Wenzel 1976; Dick and Patterson 2007). The 11 bat species that were parasitized by more than five bat fly species might have characteristics or behaviors that make them prone to being parasitized by multiple bat fly species. Several studies have shown the common presence of various bat fly species on the same bat (Wenzel 1976; Dick 2005; Hiller et al. 2018). According to Wenzel (1976), 63% of the infested Venezuelan bat species harbored two to four species of bat flies. Several species of Streblidae coexist on the same hosts, most of these associations are negatively correlated in abundance, but competition is not strong enough to lead to local extinction (Dick 2005). In fact, some of the highly connected bat host species within the interaction network for the Orinoquia were reported to be involved in some of the previously documented cases of positive co-occurrence between bat flies (Dick 2005), such as: *E.clovisi* and *Trichobiuspropinquus* on *Anourageoffroyi* (Fig. 7), *Trichobiusjoblingi* and *Streblaguajiro* on *C.perspicillata* (Fig. 8), and *Trichobiuslionycteridis* with *A.spurrelli* on *L.spurrelli* (Fig. 9).

In a lesser extent, positive correlations have also been reported in abundance, indicating mutualistic relationships (Dick 2005; Hiller et al. 2018; Alcantara et al. 2022). It is possible that the presence and greater abundance of one species of parasite facilitates the presence and abundance of the other species, reciprocally eliminating the pressure of grooming (one of the main causes of mortality of ectoparasites) in each population (Marshall 1981). Our interaction networks for the Orinoquia also revealed positive co-occurrences previously reported for ectoparasitic flies from Venezuela (Dick 2005). For example, a relatively equivalent association in the size of links and nodes is observed between *T.parasiticus* and *Streblawiedemanni*, both associated with *D.rotundus* (Fig. 8). Similarly, an equivalent relationship between *S.diaemi* and *T.diaemi* is presented in its primary host *D.youngii* (Fig. 9). Cooccurrences of Neotropical Streblids have been reported mainly between species belonging to different genera (Dick 2005; Hiller et al. 2018) that differ in their general morphology facilitating the coexistence of species by utilizing different regions on the host’s body surface (Wenzel 1976; ter Hofstede et al. 2004; Dick 2005; Hiller et al. 2018). Our interaction networks reveal the separation of the three ecomorphological groups (wing crawler – WC, fur runner – FR, and fur swimmer – FS), associated in a single bat species (Dick 2005; Hiller et al. 2018). Some notable examples of these associations include: i) *Metalasmuspseudopterus* (FS) with *A.phyllostomatis* (WC) or with *M.aranea* (FR), and *Aspidopteraphyllostomatis* (WC) with *Megistopodaaranea* (FR) in *Artibeusplanirostris*; ii) the association of *Speiseriaambigua* (FR) with *S.guajiro* (FW) or with *Trichobiusjoblingi* (WC) on *Carolliaperspicillata*, and the association of *Streblaguajiro* (FS) with *Trichobiusjoblingi* (WC) on the same host; iii) the presence of *T.parasiticus* (WC) and *Streblawiedemanni* (FS) on *D.rotundus*, and iv) the association of *Streblahertigi* (FS) with *Trichobiuscostalimai* (WC) or with *Trichobioidesperspicillatus* (WC) on *Phyllostomusdiscolor* (Fig. 8). Therefore, our results suggest ecological niche partitioning of ectoparasitic flies on bat hosts in the Orinoco region. However, due to the lack of a detailed phylogeny of bat flies, it is unclear whether these morphological differences reflect the evolutionary history of bat flies or represent convergent adaptations to host habitat type (Hiller et al. 2018).

In this study, new records are presented that describe the co-occurrence of *N.maai* and *P.parvuloides* in *N.albiventris* in the Department of Arauca, Colombia, an association previously reported by López Rivera et al. (2022) (Table 2). The positive interactions between *Noctiliostrebla* and *Paradyschria* species in *Noctilio* spp. are well known and documented (Wenzel 1976; Guerrero 1995; Guerrero 1998; Moura et al. 2003; Schad et al. 2012). For example, in the case of *N.albiventris*, *N.maai* coexists with *Paradyschiriacurvata* or *Paradyschiriaparvula*, and in the case of *Noctilioleporinus*, each individual host is infested with *Noctiliostreblaaitkeni* and *Paradyschiriafusca* or *Noctiliostreblatraubi* and *Paradyschirialineata* (Wenzel 1976; Dick 2005). In this sense, *Paradyschiria* almost always presents the highest values of prevalence and abundance, while *Noctiliostrebla* occurs more commonly in the presence of *Paradyschiria* (Moura et al. 2003; Presley 2007; Schad et al. 2012; Guerrero 2019). Our results agree with this pattern since we observed a higher abundance of *P.parvuloides* compared to *N.maai* in *N.albiventris* (Fig. 8, Table 2).

*Paradyschiria* species are fur runners, while *Noctiliostrebla* spp. are wing-crawlers, again supporting the niche partitioning of these species on *Noctilio* spp. The ecomorphological classification for *Paradyschiria* has been controversial, as they had previously been classified as wing-crawlers (Dick 2005) for not having longer hind legs, a morphological feature associated with the fur runner microhabitat (Ter Hofstede et al. 2004). However, *Paradyschiria* spp. Have an extremely strong forefemur compared to other streblid species, which allows them greater agility to move in the fur and and hold on the host’s hair (Wenzel et al. 1966; Wenzel 1976; Alcantara et al. 2022). *Noctilio* bats are considered to be among the shortest-haired of Neotropical and Nearctic bats (Dick and Miller 2010), so *Paradyschiria* flies may not need longer hind legs, otherwise a strong forefemur can be used to hold on to the host’s hair tightly if it cannot avoid grooming with rapid movements (Alcantara et al. 2022).

Finally, the analysis of ecological networks has been fundamental in the understanding of complex biological systems, providing information on how species are organized and connected in a community (Bascompte 2009; Butts 2009; Thébault and Fontaine 2010). In this sense, the study of host-parasite specificity interactions is essential to understand the mechanisms behind parasitism and its relationship with the functioning of biodiversity (Frainer et al. 2018), since parasites play an important role in the regulation of populations of host species (Poulin et al. 2006). Our results indicated that highly connected bat species in the Orinoquia interaction network act as hosts for a diversity of ectoparasitic fly species belonging to different ecomorphological groups. These groups differ in how they interact and are located on the bat’s body. Therefore, the presence of these bats is not only important for ectoparasitic fly species in general, but also for maintaining the coexistence and interaction of different ecomorphological groups of ectoparasitic flies in the network.

## ﻿Conclusions

The Orinoco Region, located in northern South America, harbors a remarkable diversity of bat flies. This diversity is largely attributed to the rich bat fauna found in Colombia and Venezuela, two countries known for their high bat diversity in the Neotropical region. In the Orinoco Region, associations have been identified between bat fly species belonging to different ecomorphological groups and unique host species. This supports the idea of a potential niche partitioning among ectoparasitic bat flies on their bat hosts. However, due to the lack of a detailed phylogeny of bat flies, it is unclear whether the observed morphological differences are the result of evolutionary history or convergent adaptations to different host habitat types. In this study, we present new records of associations between bat flies and their bat hosts, thereby expanding our knowledge of these interactions in the Orinoco Region. Overall, this study contributes to our understanding of the diversity, specificity, and ecological interactions between bat flies and their host bats in this region. These findings underscore the need for further research and sampling efforts to fill knowledge gaps in this field.

## ﻿Additional information

### Author ORCIDs

Erika M. Ospina-Pérez https://orcid.org/0000-0001-5784-6216

Fredy A. Rivera-Páez https://orcid.org/0000-0001-8048-5818

Héctor E. Ramírez-Chaves https://orcid.org/0000-0002-2454-9482

### Data availability

All of the data that support the findings of this study are available in the main text or Supplementary Information.

## Supplementary materials

Supplementary material 1 Supplementary tables Data type docx Explanation note **table S1**: Information and coordinates of the 63 sampling locations in the Orinoquia Region in South America shown on the map in Fig. 1; **table S2**: Localities of the records of flies in bats in Orinoquia from 1911 to 2023; **table S3**: Centrality metric of the species of ectoparasitic flies; **table S4**: Centrality metrics of the species of bats; **table S5**: Metrics of the interaction networks for the study areas, the first landscape: Herbaceous plains and chaparral – HPC and Orinoco-Amazonian Forests – OAF in the Colombian and Venezuelan Orinoquia Region; **table S6**: Centrality metric of the species of ectoparasitic flies, the first landscape: Herbaceous plains and chaparral – HPC and Orinoco-Amazonian Forests – OAF in the Colombian and Venezuelan Orinoquia Region; **table S7**: Centrality metric of the species of bats, the first landscape: Herbaceous plains and chaparral – HPC and Orinoco-Amazonian Forests – OAF in the Colombian and Venezuelan Orinoquia Region. https://binary.pensoft.net/file/903239This dataset is made available under the Open Database License (http://opendatacommons.org/licenses/odbl/1.0/). The Open Database License (ODbL) is a license agreement intended to allow users to freely share, modify, and use this Dataset while maintaining this same freedom for others, provided that the original source and author(s) are credited.Erika M. Ospina-Pérez, Fredy A. Rivera-Páez, Héctor E. Ramírez-Chaves

Supplementary material 2 Bipartite bat-fly quantitative network of the Herbaceous plains and chaparral – HPC Data type pdf Explanation note **fig. S1**: Bipartite bat-fly quantitative network of the Herbaceous plains and chaparral – HPC. The size of the bar on the left (red) represents the abundance (number of individuals) of bat flies per species observed and the size of the bar on the right (blue) represents the abundance of bats for which the bat fly sample was obtained. The width of the black lines/bars indicates the frequency of interactions. https://binary.pensoft.net/file/903240This dataset is made available under the Open Database License (http://opendatacommons.org/licenses/odbl/1.0/). The Open Database License (ODbL) is a license agreement intended to allow users to freely share, modify, and use this Dataset while maintaining this same freedom for others, provided that the original source and author(s) are credited.Erika M. Ospina-Pérez, Fredy A. Rivera-Páez, Héctor E. Ramírez-Chaves

Supplementary material 3 Bipartite bat-fly quantitative network of the Orinoco-Amazonian Forests – OAF https://doi.org/10.3897/zookeys.1179.103479.suppl3 Data type pdf Explanation note **fig. S2**: Bipartite bat-fly quantitative network of the Orinoco-Amazonian Forests – OAF. The size of the bar on the left (red) represents the abundance (number of individuals) of bat flies per species observed and the size of the bar on the right (blue) represents the abundance of bats for which the bat fly sample was obtained. The width of the black lines/bars indicates the frequency of interactions. https://binary.pensoft.net/file/903241This dataset is made available under the Open Database License (http://opendatacommons.org/licenses/odbl/1.0/). The Open Database License (ODbL) is a license agreement intended to allow users to freely share, modify, and use this Dataset while maintaining this same freedom for others, provided that the original source and author(s) are credited.Erika M. Ospina-Pérez, Fredy A. Rivera-Páez, Héctor E. Ramírez-Chaves
