# Supplementary material for: ﻿Exploring the relationship between bats (Mammalia, Chiroptera) and ectoparasitic flies (Diptera, Hippoboscoidea) of the Orinoquia Region in South America
Source: Zookeys. 2023 Sep 8;1179:1–34. doi: 10.3897/zookeys.1179.103479 (PMC10504637; doi:10.3897/zookeys.1179.103479)
Supplement: Supplementary material 1 — Supplementary tables [file zookeys-1179-001_article-103479__-s001.docx]

**Table S1.** Information and coordinates of the 63 sampling locations in the Orinoquia Region in South America shown on the map in Figure 1.

| **Number** | **Country** | **Localities** | **Decimal Latitude (N)** | **Verbatim Longitude (W)** |
| --- | --- | --- | --- | --- |
| 1 | Venezuela | Aragua: Maracay, 4 Km NW, El Limon | 10.28 | -67.6 |
| 2 | Venezuela | Apure: 29 km SSW Santo Domingo. Selvas de San Camilo, Nulita, 24m | 7.32 | -71.95 |
| 3 | Venezuela | Apure: 1 km W Pto. Páez, Cerro de Murcielagos, Pto. Páez, 76m | 6.2 | -67.45 |
| 4 | Venezuela | Apure: Hato Cariben, 32 Km NE Pto. Paez, La Villa | 6.55 | -67.22 |
| 5 | Venezuela | Amazonas: 108 km SSE Esmeralda, Río Mavaca, 140 m | 2.25 | -65.28 |
| 6 | Venezuela | Amazonas: 163 Km ESE Pto. Ayacucho, Río Manapiare, San Juan, 155 m | 5.35 | -66.18 |
| 7 | Venezuela | Amazonas: Boca Mavaca, 84 Km SSE Esmeralda | 2.5 | -65.22 |
| 8 | Venezuela | Amazonas: Pto. Ayacucho, 32 Km S Pto. Ayacucho, Raya | 5.4 | -67.65 |
| 9 | Venezuela | Amazonas: Rio Mavaca, 108 Km SSE Esmeralda | 2.08 | -65.3 |
| 10 | Venezuela | Amazonas: San Juan, 163 Km ESE Pto. Ayacucho, Rio Manapiare | 5.3 | -66.22 |
| 11 | Venezuela | Amazonas: Tamatama, Rio Orinoco | 3.17 | -65.82 |
| 12 | Venezuela | Amazonas: Belén, 56 Km NNW Esmeralda, Rio Cunucunuma | 3.65 | -65.77 |
| 13 | Venezuela | Amazonas: Cabecera del Caño Culebra, 40 km NNW Esmeralda, Cerro Duida, 1400 m | 3.5 | -65.72 |
| 14 | Venezuela | Amazonas: 65 km SSW Pto. Ayacucho, nr. Morganito, Pto. Ayacucho, 161 m | 5.1 | -67.75 |
| 15 | Venezuela | Barinas: Altamira, Altamira | 8.83 | -70.5 |
| 16 | Venezuela | Bolívar: Serranía de Nuria, | 7.676972 | -61.617091 |
| 17 | Venezuela | Bolívar: 5 km NNW Guasipati, Guasipati, 190 m | 7.472474 | -61.892381 |
| 18 | Venezuela | Bolívar: Hato La Florida, 47 Km ESE Caicara | 7.5 | -65.78 |
| 19 | Venezuela | Bolívar: km. 125, 85 Km SSE El Dorado | 5.98 | -61.43 |
| 20 | Venezuela | Bolívar: 28 km SE El Manteco, Los Patos, 150 m | 7.18 | -62.37 |
| 21 | Venezuela | Bolívar: Rio Supamo, 50 Km SE El Manteco | 7 | -62.25 |
| 22 | Venezuela | Bolívar: 20 km W La Paragua, Hato San José, 306 m | 6.82 | -63.48 |
| 23 | Venezuela | Bolívar: 56 km SE El Dorado, km 74, El Manaco, 150m | 6.32 | -61.32 |
| 24 | Venezuela | Bolívar: 59 km SE El Dorado, km 74, El Manaco, 150 m | 6.28 | -61.32 |
| 25 | Venezuela | Bolívar: 21 km NE Icabarú, Icabarú, 750 m | 4.42 | -61.58 |
| 26 | Venezuela | Bolívar: 11 km NE Icabarú, Icabarú , 750m | 4.4 | -61.63 |
| 27 | Venezuela | Bolívar: 70 km SSE El Dorado, Piedra Virgen, KM 125, 374 M | 6.15 | -61.37 |
| 28 | Venezuela | Carabobo: 4 km NW Montalbán, La Copa, Montalbán, 1537 m, 29/09/1967 | 10.25 | -68.35 |
| 29 | Venezuela | Guárico: 35 km SSW San Juan de los Morros, Hto. Las Palmitas, 181m | 8.87 | -67.38 |
| 30 | Venezuela | Guárico: 35 km SSW San Juan de los Morros, Hto. Las Palmitas, 181m | 9.6 | -67.45 |
| 31 | Venezuela | Guárico: 14 km SE Calabozom nr. Ría Orituco, Estaction Biologica de los Lllanos, 100m | 8.82 | -67.37 |
| 32 | Venezuela | Guárico: 10 km NE Altagracia, Hda. El Vira, 630m | 9.92 | -66.32 |
| 33 | Venezuela | Merida: Tabay, 4 Km E Tabay, La Mucuy | 8.63 | -71.03 |
| 34 | Venezuela | Merida: La Carbonera, 12 Km SE La Azulita | 8.63 | -71.35 |
| 35 | Venezuela | Monagas: San Agustin, 5 Km NW Caripe | 10.2 | -63.53 |
| 36 | Venezuela | Monagas: 3 km Sw Caripe, 854m | 10.17 | -63.5 |
| 37 | Venezuela | Monagas: 55 km SSE Maturín, Hato Mata de Bejueo, 18m. | 9.32 | -62.93 |
| 38 | Venezuela | Trujillo: Valera, 25 Km NW Valera, Nr. Agua Santa | 9.53 | -70.67 |
| 39 | Venezuela | Trujillo: Valera, 23 Km N Valera, Nr. Agua Viva | 9.52 | -70.58 |
| 40 | Venezuela | Yaracuy: 20 km NW San Felipe, Minas de Aroa, 380-400 m | 10.42 | -68.9 |
| 41 | Venezuela | Yaracuy: 10 km NW Urama, El Central, Urama, El Central, Urama, 25 m | 10.53 | -68.38 |
| 42 | Colombia | Meta: Los Micos, San Juan de Arama, 1300 | 3.317186 | -73.90658 |
| 43 | Colombia | Vichada: El Parque Tuparro, Centro Administrativo | 5.269972 | -68.608581 |
| 44 | Colombia | Meta: El Parque La Macarena | 2.968454 | -73.900075 |
| 45 | Colombia | Guainía: Vichada, Cumaribo, Cerca Amanaven | 4.093696 | -67.738437 |
| 46 | Colombia | Casanare: Trinidad, Reserva Natural La Palmita, en la vereda La Cañada | 5.4202778 | -71.5997222 |
| 47 | Colombia | Casanare: Orocué | 4.793151 | -71.342066 |
| 48 | Colombia | Meta: La Macarena, Rio Guapaya | 2.885336 | -73.651656 |
| 49 | Colombia | Meta: Villavicencio, Quebrada Honda | 4.257318 | -73.688515 |
| 50 | Colombia | Arauca: Arauca, Vereda Las Plumas | 6.6111111 | -70.5308333 |
| 51 | Colombia | Arauca: Arauca, Vereda Las Plumas | 6.6169444 | -70.525 |
| 52 | Colombia | Arauca: Arauca, Vereda Las Plumas | 6.6041667 | -70.4977778 |
| 53 | Colombia | Arauca: Arauca, Vereda El Socorro | 6.7775 | -70.7069444 |
| 54 | Colombia | Arauca: Arauca, Universidad Nacional Sede Orinoquia | 7.0147222 | -70.7433333 |
| 55 | Colombia | Arauca: Cravo Norte, Vereda El Deleite | 6.5375 | -70.5205556 |
| 56 | Colombia | Arauca: Tame, Vereda Santa Inés | 6.57675 | -70.7166667 |
| 57 | Colombia (New records) | Arauca: Arauca, Vereda El Socorro, Finca Los Trompillos | 6.7797222 | -70.7164722 |
| 58 | Colombia (New records) | Arauca: Arauca, Vereda El Socorro, Finca Marsella | 6.7786667 | -70.7266944 |

**Table S2.** Localities of the records of flies in bats in Orinoquia from 1911 to 2023. Some specific epythets for taxa within some genera (e.g., *Pteronotus*, *Artibeus*, *Anoura*, *Dermanura*, *Platyrrhinus*, and *Sturnira*), are still in need of an update by direct examination of the voucher specimens.

| **bat-flies** | **Host** | **N** | **Locality** | **Departament** | **Country** | **Collection date** | |
| --- | --- | --- | --- | --- | --- | --- | --- |
| *Nycterophilia coxata* | *Pteronotus parnellii* (probably *P. fuscus*) | 41 | Altagracia, Hda. El Vira | Guárico | Venezuela | 16/09/1966 |  |
|  | *Pteronotus parnellii* | 5 | Caripe, San Agustín | Monagas | Venezuela | 26/06/1967 |  |
|  | *Phyllostomus elongatus* | 1 | La Paragua, Hato San José | Bolívar | Venezuela | 4/04/1967 |  |
|  | *Pteronotus parnellii* | 3 | Esmeralda, Río Mavaca | Amazonas | Venezuela | 3/04/1967 | 11/04/1967 |
|  | *Eumops glaucinus* | 1 | Pto. Ayacucho, Río Manapiare, San Juan | Amazonas | Venezuela | 17/07/1967 |  |
|  | *Artibeus planirostris* | 1 | Valera, nr. Agua Viva | Trujillo | Venezuela | 23/10/1965 |  |
|  | *Pteronotus davyi* | 3 | San Felipe, Minas de Aroa | Yaracuy | Venezuela | 6/12/1967 | 23/12/1967 |
|  | *Pteronotus parnellii* | 286 | San Felipe, Minas de Aroa | Yaracuy | Venezuela | 6/12/1967 | 23/12/1967 |
|  | *Pteronotus gymnonotus* | 5 | San Felipe, Minas de Aroa | Yaracuy | Venezuela | 6/12/1967 | 23/12/1967 |
|  | *Pteronotus parnellii* | 2 | Urama, El Central, Urama | Yaracuy | Venezuela | 14/03/1966 |  |
| *Nycterophilia fairchildi* | *Pteronotus davyi* | 16 | San Felipe, Minas de Aroa | Yaracuy | Venezuela | 6/12/1967 | 23/12/1967 |
|  | *Pteronotus parnellii* | 1 | San Felipe, Minas de Aroa | Yaracuy | Venezuela | 6/12/1967 | 23/12/1967 |
|  | *Pteronotus gymnonotus* | 27 | San Felipe, Minas de Aroa | Yaracuy | Venezuela | 6/12/1967 | 23/12/1967 |
|  | *Platyrrhinus helleri* | 1 | San Felipe, Minas de Aroa | Yaracuy | Venezuela | 6/12/1967 | 23/12/1967 |
| *Nycterophilia mormoopsis* | *Mormoops megalophylla* | 1 | San Felipe, Minas de Aroa | Yaracuy | Venezuela | 11/12/1967 |  |
| *Nycterophilia parnelli* | *Pteronotus parnellii* | 1 | Pto. Páez, La Villa, Hato Cariben | Apure | Venezuela | 24-dic | 28/12/1965 |
|  | *Lonchorhina orinocensis* | 1 | Pto. Páez, La Villa, Hato Cariben | Apure | Venezuela | 24/12/1967 | 28/12/1965 |
|  | *Pteronotus parnellii* | 1 | Pto. Páez, Cerro de Murcielagos, Pto. Páez | Apure | Venezuela | 19/01/1966 |  |
|  | *Pteronotus parnellii* | 95 | Guasipati, Guasipati | Bolívar | Venezuela | 29/04/1966 |  |
|  | *Pteronotus parnellii* | 11 | Caicara, Hato La Florida | Bolívar | Venezuela | 29/04/1967 | 24/04/1967 |
|  | *Pteronotus parnellii* | 1 | El Dorado | Bolívar | Venezuela | 11-05.1966 |  |
|  | *Pteronotus parnellii* | 1 | El Manteco, Los patos | Bolívar | Venezuela | 11/04/1966 |  |
|  | *Pteronotus parnellii* | 14 | El Manteco, Río Supamo | Bolívar | Venezuela | 10/04/1966 |  |
|  | *Pteronotus parnellii* | 2 | Esmeralda, Boca Mavaca | Amazonas | Venezuela | 17/03/1967 | 23/03/1967 |
|  | *Pteronotus parnellii* | 9 | Pto. Ayacucho, Raya, Pto. Ayacucho | Amazonas | Venezuela | 6/09/1967 | 2/10/1967 |
|  | *Pteronotus parnellii* | 45 | Esmeralda, Río Mavaca | Amazonas | Venezuela | 3/04/1967 | 11/04/1967 |
|  | *Sturnira tildae* | 1 | Esmeralda, Río Mavaca | Amazonas | Venezuela | 3/04/1967 | 11/04/1967 |
|  | *Pteronotus parnellii* | 5 | Pto. Ayacucho, Río Manapiare, San Juan | Amazonas | Venezuela | 12/07/1967 | 27/07/1967 |
|  | *Pteronotus parnellii* | 10 | Río Orinoco, Tamatama | Amazonas | Venezuela | 27/04/1967 |  |
|  | *Pteronotus davyi* | 2 | San Felipe, Minas de Aroa | Yaracuy | Venezuela | 21/12/1967 | 23/12/1967 |
|  | *Lonchorhina aurita* | 1 | Montalban, La Leonera | Carabobo | Venezuela | 22/09/1967 |  |
|  | *Pteronotus rubiginosus* | 11 | Serranía de Nuria | Bolívar | Venezuela | 31/08/1962 |  |
| *Phalcophila puliciformis* | *Lonchophylla robusta* | 1 | Altamira | Barinas | Venezuela | 8/01/1968 |  |
|  | *Artibeus planirostris* | 1 | Altamira | Barinas | Venezuela | 8/01/1968 |  |
| *Anatrichobius scorzai* | *Lonchophylla robusta* | 1 | Altamira, Altamira | Barinas | Venezuela | 26/07/1967 |  |
|  | *Myotis keaysi* | 3 | Montalbán, La Copa | Carabobo | Venezuela | 28/11/1967 |  |
|  | *Myotis oxyotus* | 1 | Icabarú, El Pauji, Icabarú | Bolívar | Venezuela | 3/05/1968 |  |
|  | *Myotis oxyotus* | 1 | El Dorado | Bolívar | Venezuela | 16/05/1966 |  |
| *Aspidoptera falcata* | *Sturnira lilium* (probably *S. giannae*) | 17 | Altamira, Altamira | Barinas | Venezuela | 27/07/1967 | 4/01/1968 |
|  | *Sturnira lilium* (probably *S. giannae*) | 3 | Altamira, Altamira | Barinas | Venezuela | 26/07/1967 |  |
|  | *Sturnira lilium* (probably *S. giannae*) | 13 | Altamira | Barinas | Venezuela | 19/07/1967 | 9/01/1968 |
|  | *Sturnira lilium* (probably *S. giannae*) | 12 | El Dorado km 67, El Manaco | Bolívar | Venezuela | 16/06/1966 |  |
|  | *Sturnira lilium* (probably *S. giannae*) | 31 | El Dorado Km 74, El Manaco | Bolívar | Venezuela | 8/06/1966 | 25/06/1966 |
|  | *Sturnira lilium* (probably *S. giannae*) | 1 | El Dorado, nr. Río Danta, El Manaco | Bolívar | Venezuela | 24/06/1966 |  |
|  | *Sturnira lilium* (probably *S. giannae*) | 10 | Icabarú, Santa Lucia | Bolívar | Venezuela | 29/04/1968 | 2/05/1968 |
|  | *Sturnira lilium* (probably *S. giannae*) | 6 | El Dorado | Bolívar | Venezuela | 9/05/1966 |  |
|  | *Sturnira lilium* (probably *S. giannae*) | 10 | El Manteco, Los Patos | Bolívar | Venezuela | 11/04/1966 |  |
|  | *Sturnira lilium* (probably *S. giannae*) | 3 | El Manteco, Río Supamo | Bolívar | Venezuela | 10/04/1966 |  |
|  | *Sturnira lilium* (probably *S. giannae*) | 1 | Esmeralda, Caño Culebra, Belén | Amazonas | Venezuela | 2/11/1967 |  |
|  | *Sturnira lilium* (probably *S. giannae*) | 2 | Esmeralda, Río Cunucunuma, Belén | Amazonas | Venezuela | 2/11/1967 | 10/11/1967 |
|  | *Sturnira lilium* (probably *S. giannae*) | 21 | Pto. Ayacucho, Paria | Amazonas | Venezuela | 13/09/1967 | 6/10/1967 |
|  | *Sturnira lilium* (probably *S. giannae*) | 2 | Pto. Ayacucho, Guayabal | Amazonas | Venezuela | 7/10/1967 |  |
|  | *Sturnira lilium* (probably *S. giannae*) | 3 | Pto. Ayacucho, Coromoto | Amazonas | Venezuela | 8/10/1967 | 9/10/1967 |
|  | *Sturnira lilium* (probably *S. giannae*) | 8 | Pto. Ayacucho, Raya, Pto. Ayacucho | Amazonas | Venezuela | 6/09/1967 | 7/09/1967 |
|  | *Sturnira lilium* (probably *S. giannae*) | 17 | Pto. Ayacucho, Río Manapiare, San Juan | Amazonas | Venezuela | 6/07/1967 | 24/07/1967 |
|  | *Sturnira lilium* (probably *S. giannae*) | 2 | Montalbán, Potrerito | Carabobo | Venezuela | 31/10/1967 | 1/11/1967 |
|  | *Sturnira lilium* (probably *S. giannae*) | 5 | Montalbán, La Copa | Carabobo | Venezuela | 22/11/1967 |  |
|  | *Sturnira lilium* (probably *S. giannae*) | 3 | Montalban, La Copa | Carabobo | Venezuela | 28/11/1967 |  |
|  | *Sturnira ludovici* | 1 | Montalbán, La Voluntad | Carabobo | Venezuela |  |  |
|  | *Sturnira ludovici* | 1 | Montalbán, Potrerito | Carabobo | Venezuela |  |  |
|  | *Sturnira ludovici* | 7 | Montalbán, La Copa | Carabobo | Venezuela |  |  |
|  | *Sturnira lilium* (probably *S. giannae*) | 10 | Valera, nr. Agua Viva | Trujillo | Venezuela | 3/09/1965 | 7/10/1965 |
|  | *Sturnira lilium* (probably *S. giannae*) | 3 | Valera, nr. Agua Viva | Trujillo | Venezuela | 3/09/1965 | 7/10/1965 |
|  | *Sturnira lilium* (probably *S. giannae*) | 5 | Valera, nr. Agua Viva | Trujillo | Venezuela | 17/09/1965 |  |
|  | *Sturnira lilium* (probably *S. giannae*) | 11 | Urama, El Central, Urama | Yaracuy | Venezuela | 15/08/1965 | 18/10/1965 |
|  | *Sturnira lilium* (probably *S. giannae*) | 50 | Santo Domingo, Selvas de San Camilo, Nulita | Apure | Venezuela | 17/01/1968 | 2/02/1968 |
|  | *Carollia perspicillata* | 1 | Santo Domingo, Selvas de San Camilo, Nulita | Apure | Venezuela | 17/01/1968 | 2/02/1968 |
|  | *Sturnira ludovici* | 1 | Santo Domingo, Selvas de San Camilo, Nulita | Apure | Venezuela | 17/01/1968 | 2/02/1968 |
|  | *Sturnira ludovici* | 15 | Altamira, Altamira | Barinas | Venezuela | 26/07/1967 | 3/01/1968 |
|  | *Sturnira ludovici* | 3 | Altamira | Barinas | Venezuela | 21/07/1967 | 10/01/1968 |
|  | *Sturnira lilium* (probably *S. giannae*) | 1 | Calabozo, nr. Río Orituco, Estación Biologicas de los Llanos | Guárico | Venezuela | 21/08/1968 |  |
|  | *Sturnira lilium* (probably *S. giannae*) | 1 | Calabozo, Estación Biologicas de los Llanos | Guárico | Venezuela | 19/08/1968 |  |
|  | *Sturnira lilium* (probably *S. giannae*) | 6 | Altagracia, Hda. El Vira | Guárico | Venezuela | 16/09/1966 |  |
|  | *Sturnira lilium* (probably *S. giannae*) | 6 | Altagracia, Río Orituco | Guárico | Venezuela | 20/09/1966 |  |
|  | *Sturnira ludovici* | 15 | Altagracia | Guárico | Venezuela | 16/09/1966 |  |
|  | *Sturnira lilium* (probably *S. giannae*) | 3 | Caripe, nr. San Agustín | Monagas | Venezuela | 11/07/1967 |  |
|  | *Sturnira lilium* (probably *S. giannae*) | 4 | Caripe, nr. San Agustín | Monagas | Venezuela | 11/07/1967 |  |
|  | *Sturnira ludovici* | 2 | Caripe, San Agustín | Monagas | Venezuela | 11/07/1967 |  |
|  | *Dermanura cinerea* | 1 | El Manaco | Bolívar | Venezuela | 25/06/1966 |  |
|  | *Sturnira tildae* | 6 | Icabarú, Santa Lucia de Surukun | Bolívar | Venezuela | 30/04/1968 | 2/05/1968 |
|  | *Sturnira tildae* | 6 | El Dorado | Bolívar | Venezuela | 9/05/1966 | 23/05/1966 |
|  | *Artibeus planirostris* | 1 | Pto. Ayacucho, Río Manapiare, San Juan | Amazonas | Venezuela | 24/07/1967 | 27/07/1967 |
|  | *Phyllostomus hastatus* | 1 | Pto. Ayacucho, Río Manapiare, San Juan | Amazonas | Venezuela | 24/07/1967 | 27/07/1967 |
|  | *Artibeus obscurus* | 2 | Esmeralda, Río Cunucunuma, Belén | Amazonas | Venezuela | 1/02/1967 | 2/02/1967 |
|  | *Sturnira tildae* | 57 | Esmeralda, Río Cunucunuma, Belén | Amazonas | Venezuela | 1/02/1967 | 2/02/1967 |
|  | *Sturnira tildae* | 52 | Esmeralda, Caño Culebra, Belén | Amazonas | Venezuela | 2/02/1967 | 3/02/1967 |
|  | *Sturnira tildae* | 8 | Esmeralda, Boca Macava, | Amazonas | Venezuela | 6/03/1967 |  |
|  | *Sturnira tildae* | 1 | Esmeralda, Brazo Casiquiare, Capibara | Amazonas | Venezuela | 29/05/1967 |  |
|  | *Sturnira tildae* | 1 | Río Orinoco, Tamatama | Amazonas | Venezuela | 30/04/1967 |  |
|  | *Sturnira tildae* | 11 | Esmeralda, Río Mavaca | Amazonas | Venezuela | 14/04/1967 |  |
|  | *Uroderma bilobatum* | 1 | Urama, El Central | Yaracuy | Venezuela | 14/03/1966 |  |
| *Aspidoptera phyllostomatis* | *Artibeus planirostris* | 355 | Apure (1 localitv); Barinas (2 localities); Bolivar (3 localities); Carabobo (4 localities); Falcon (5 localities); Guarico (1 locality); Lara (1 locality); Miranda (5 localities); Monagas (2 localities); Amazonas (6 localities); Trujillo (5 localities); Yaracuv (1 locality) ; Zulia ( 7 localities) | Apure, Barinas, Bolívar, Carabobo, Falcon, Guarico, Lara, Miranda, Monagas, Amazonas, Trujillo y Zulia | Venezuela | 1960 | 1967 |
|  | *Artibeus planirostris* | 9 | Tame, Vereda Santa Inés | Arauca | Colombia | 15/03/2019 |  |
|  | *Artibeus planirostris* | 1 | Cravo Norte, Vereda Las plmas, El deleite | Arauca | Colombia | 26/11/2018 |  |
|  | *Phyllostomus discolor* | 1 | Tame, Vereda Santa Inés | Arauca | Colombia | 12/03/2019 |  |
|  | *Sturnira giannae* | 1 | Tame, Vereda Santa Inés | Arauca | Colombia | 4/03/2019 |  |
|  | *Artibeus lituratus* | 2 | Reserva Natural La Palmita | Casanare | Colombia | 1/10/2017 | nov-17 |
| *Aspidoptera delatorrei* | *Artibeus planirostris* | 1 | Arauca, Vereda Las Plumas, Finca Los iguanitos | Arauca | Colombia | 9/11/2018 |  |
|  | *Artibeus planirostris* | 2 | Tame | Arauca | Colombia | 2018 | 2019 |
|  | *Sturnira giannae* | 3 | Tame | Arauca | Colombia | 2018 | 2019 |
|  | *Sturnira* cf*. parvidens* | 15 | Tame | Arauca | Colombia | 2018 | 2019 |
|  | *Sturnira* sp. | 1 | Tame | Arauca | Colombia | 2018 | 2019 |
| *Exastinion clovisi* | *Anoura caudifer* | 1 | Altamira, Altamira | Barinas | Venezuela | 20/07/1967 |  |
|  | *Anoura geoffroyi* | 2 | Altamira, Altamira | Barinas | Venezuela | 20/07/1967 |  |
|  | *Anoura geoffroyi* | 17 | El Dorado, Km 74, El Manaco | Bolívar | Venezuela | 9/06/1966 | 23/06/1966 |
|  | *Anoura* sp. | 9 | El Dorado, Km 74, El Manaco | Bolívar | Venezuela | 9/06/1966 | 23/06/1966 |
|  | *Anoura* sp. | 18 | El Dorado, km 125 | Bolívar | Venezuela | 10/05/1966 | 26/05/1966 |
|  | *Anoura geoffroyi* | 37 | El Dorado, km 125 | Bolívar | Venezuela | 10/05/1966 | 26/05/1966 |
|  | *Anoura geoffroyi* | 7 | La Paragua, Hato San José | Bolívar | Venezuela | 4/04/1967 | 10/04/1967 |
|  | *Anoura geoffroyi* | 4 | Icabarú, El Pauji, Icabarú | Bolívar | Venezuela | 7/05/1968 |  |
|  | *Anoura* sp. | 6 | Icabarú, El Pauji, Icabarú | Bolívar | Venezuela | 4/05/1967 |  |
|  | *Anoura caudifer* | 13 | Montalbán, La Copa | Carabobo | Venezuela | 26/11/1967 | 30/11/1967 |
|  | *Anoura geoffroyi* | 6 | Montalbán, La Copa | Carabobo | Venezuela | 26/11/1967 | 30/11/1967 |
|  | *Anoura* sp. | 8 | Montalbán, La Copa | Carabobo | Venezuela | 26/11/1967 | 30/11/1967 |
|  | *Anoura geoffroyi* | 5 | Montalbán, Potrerito | Carabobo | Venezuela | 1/11/1967 |  |
|  | *Anoura geoffroyi* | 3 | Altagracia, Hda. El Vira | Guárico | Venezuela | 19/06/1966 |  |
|  | *Anoura geoffroyi* | 10 | Caripe, San Agustín | Monagas | Venezuela | 25/06/1967 | 6/07/1967 |
|  | *Peropteryx macrotis* | 2 | Pto. Ayacucho, Platanilla | Amazonas | Venezuela | 12/10/1967 | 13/10/1967 |
|  | *Anoura geoffroyi* | 2 | Pto. Ayacucho, Platanilla | Amazonas | Venezuela | 12/10/1967 | 13/10/1967 |
|  | *Anoura geoffroyi* | 23 | Pto. Ayacucho, Río Manapiare, San Juan | Amazonas | Venezuela | 21/07/1967 | 28/07/1967 |
|  | *Anoura* sp. | 47 | Pto. Ayacucho, Río Manapiare, San Juan | Amazonas | Venezuela | 21/07/1967 | 28/07/1967 |
|  | *Artibeus planirostris* | 1 | Pto. Ayacucho, Río Manapiare, San Juan | Amazonas | Venezuela | 21/07/1967 | 28/07/1967 |
|  | *Anoura geoffroyi* | 21 | Cabecera del Caño Culebra, Esmeralda | Amazonas | Venezuela | 4/11/1967 | 8/11/1967 |
|  | *Anoura geoffroyi* | 4 | Pto. Ayacucho, El Gavilan | Amazonas | Venezuela | 11/10/1967 |  |
|  | *Anoura geoffroyi* | 9 | Pto. Ayacucho, Las Queseras | Amazonas | Venezuela | 21/09/1967 |  |
|  | *Anoura latidens* | 9 | Tame, Vereda Santa Inés, Finca La Porfiria | Arauca | Colombia | 12/03/2019 |  |
| *Exastinion oculatum* | *Anoura cultrata* | 8 | Rancho Grande, El Limón | Aragua | Venezuela | 30/03/1960 |  |
| *Exastinion deceptivum* | *Anoura geoffroyi* | 9 | Tabay, Middle Refugio | Merida | Venezuela | 15/04/1966 |  |
|  | *Anoura geoffroyi* | 7 | San Agustín, Caripa | Monagas | Venezuela | 27/06/1967 | 3/07/1967 |
| *Mastoptera guimaraesi* | *Phyllostomus hastatus* | 14 | Santo Domingo, Selvas de San Camilo, Nulita | Apure | Venezuela | 17/01/1968 | 31/01/1968 |
|  | *Phyllostomus hastatus* | 1 | Altamira, Altamira | Barinas | Venezuela | 26/07/1967 |  |
|  | *Phyllostomus hastatus* | 1 | Altamira, Altamira | Barinas | Venezuela | 10/01/1968 |  |
|  | *Phyllostomus hastatus* | 2 | Valera, nr. Agua Viva | Trujillo | Venezuela | 5/09/1965 | 7/10/1965 |
|  | *Phyllostomus hastatus* | 2 | Valera, nr. Agua Viva | Trujillo | Venezuela | 22/10/1965 |  |
|  | *Phyllostomus hastatus* | 4 | Urama, El Central | Yaracuy | Venezuela | 14/03/1966 |  |
|  | *Phyllostomus hastatus* | 5 | Urama, El Central | Yaracuy | Venezuela | 20/03/1967 |  |
|  | *Phyllostomus hastatus* | 12 | Arauca, Los Trompillos | Arauca | Colombia | 1/11/2021 |  |
| *Mastoptera minuta* | *Lophostoma silvicola* | 3 | Esmeralda, Río Cunucunuma, Belén | Amazonas | Venezuela | 1/01/1967 |  |
|  | *Lophostoma silvicola* | 35 | Esmeralda, Brazo Casiquiare, Capibara | Amazonas | Venezuela | 30/05/1967 | 12/06/1967 |
|  | *Lophostoma silvicola* | 2 | Pto. Ayacucho, nr. Morganito | Amazonas | Venezuela | 9/10/1967 |  |
|  | *Lophostoma silvicola* | 2 | Esmeralda, Río Mavaca | Amazonas | Venezuela | 5/04/1967 | 12/04/1967 |
|  | *Lophostoma silvicola* | 1 | Pto. Ayacucho, Río Manapiare, San Juan | Amazonas | Venezuela | 24/07/1967 |  |
|  | *Lophostoma silvicola* | 10 | Valera, nr. Agua Santa | Trujillo | Venezuela | 20-08-1065 | 18/10/1965 |
|  | *Tonatia saurophila* | 16 | Reserva Natural La Palmita | Casanare | Colombia | 2017 |  |
| *Mastoptera minuta s.l.* | *Phyllostomus elongatus* | 2 | Pto. Ayacucho, Paria | Amazonas | Venezuela | 14/09/1967 | 5/10/1967 |
|  | *Phyllostomus hastatus* | 13 | Pto. Ayacucho, Paria | Amazonas | Venezuela | 14/09/1967 | 5/10/1967 |
|  | *Phyllostomus hastatus* | 23 | Pto. Ayacucho, Raya, Pto. Ayacucho | Amazonas | Venezuela | 6/09/1967 | 10/10/1967 |
|  | *Lophostoma brasiliense* | 1 | Pto. Ayacucho, nr. Morganito | Amazonas | Venezuela | 8/10/1967 |  |
|  | *Lophostoma silvicola* | 1 | Pto. Ayacucho, nr. Morganito | Amazonas | Venezuela | 8/10/1967 |  |
|  | *Lophostoma brasiliense* | 17 | Pto. Ayacucho, Río Manapiare, San Juan | Amazonas | Venezuela | 5/07/1967 | 28/07/1967 |
|  | *Lophostoma silvicola* | 1 | Pto. Ayacucho, Río Manapiare, San Juan | Amazonas | Venezuela | 5/07/1967 | 28/07/1967 |
|  | *Anoura* sp. | 8 | Pto. Ayacucho, Río Manapiare, San Juan | Amazonas | Venezuela | 5/07/1967 | 28/07/1967 |
|  | *Artibeus obscurus* | 1 | Pto. Ayacucho, Río Manapiare, San Juan | Amazonas | Venezuela | 5/07/1967 | 28/07/1967 |
|  | *Artibeus lituratus* | 1 | Pto. Ayacucho, Río Manapiare, San Juan | Amazonas | Venezuela | 5/07/1967 | 28/07/1967 |
|  | *Artibeus planirostris* | 1 | Pto. Ayacucho, Río Manapiare, San Juan | Amazonas | Venezuela | 5/07/1967 | 28/07/1967 |
|  | *Phyllostomus elongatus* | 1 | Pto. Ayacucho, Río Manapiare, San Juan | Amazonas | Venezuela | 5/07/1967 | 28/07/1967 |
|  | *Phyllostomus hastatus* | 64 | Pto. Ayacucho, Río Manapiare, San Juan | Amazonas | Venezuela | 5/07/1967 | 28/07/1967 |
|  | *Phyllostomus hastatus* | 29 | Esmeralda, Brazo Casiquiare, Capibara | Amazonas | Venezuela | 29/05/1967 | 30/05/1967 |
|  | *Lophostoma silvicola* | 35 | Esmeralda, Brazo Casiquiare, Capibara | Amazonas | Venezuela | 29/05/1967 | 30/05/1967 |
|  | *Phyllostomus hastatus* | 2 | Esmeralda, Río Mavaca | Amazonas | Venezuela | 4/04/1967 | 14/04/1967 |
|  | *Phyllostomus elongatus* | 31 | Esmeralda, Río Mavaca | Amazonas | Venezuela | 4/04/1967 | 14/04/1967 |
|  | *Lophostoma carrikeri* | 19 | Esmeralda, Río Mavaca | Amazonas | Venezuela | 4/04/1967 | 14/04/1967 |
|  | *Lophostoma silvicola* | 2 | Esmeralda, Río Mavaca | Amazonas | Venezuela | 4/04/1967 | 14/04/1967 |
|  | *Lophostoma silvicola* | 3 | Esmeralda, Río Cunucunuma, Belén | Amazonas | Venezuela | 3/01/1967 |  |
|  | *Phyllostomus hastatus* | 5 | Río Orinoco, Tamatama | Amazonas | Venezuela | 1/05/1967 |  |
|  | *Lophostoma brasiliense* | 29 | Valera, nr. Agua Viva | Trujillo | Venezuela | 15/09/1965 |  |
|  | *Lophostoma silvicola* | 10 | Valera, nr. Agua Santa | Trujillo | Venezuela | 20/08/1965 | 18/10/1965 |
|  | *Lophostoma brasiliense* | 1 | Urama, El Central | Yaracuy | Venezuela | 14/03/1966 | 22/03/1966 |
|  | *Phyllostomus hastatus* | 1 | Urama, El Central | Yaracuy | Venezuela | 14/03/1966 | 22/03/1966 |
| *Megistopoda proxima* | *Noctilio albiventris* | 3 | Arauca, Vereda Las Plumas, Sector Guayabital | Arauca | Colombia | 12/11/2018 |  |
|  | *Carollia perspicillata* | 1 | Tame, Vereda Santa Inés, Finca La Porfiria | Arauca | Colombia | 4/03/2019 |  |
|  | *Sturnira giannae* | 21 | Tame | Arauca | Colombia | 2018 | 2019 |
|  | *Sturnira parvidens* | 23 | Tame | Arauca | Colombia | 2018 | 2019 |
|  | *Sturnira* sp*.* | 2 | Tame | Arauca | Colombia | 2018 | 2019 |
| *Megistopoda aranea* | *Artibeus planirostris* | 530 | Apure (2 localities, 24-76 m); Barinas (1 locality, 609-611 m); Bolivar (3 localities, 150-324 n'l); Carahobo (4 localities, 598-1,007 m); Dto. Federal (4 localities, 398-2,050 m); Falcon (7 localities, 2-4S0 m); Guarico (5 localities, 100-630 m); Lara (1 localit)', 528 m); Miranda (5 localities, 1-1,180 m); Monagas (1 localit)', 1,170 m); Amazonas (9 localities, 119-1,524 m); Tnijillo (4 localities, 28-900 m); Yaracuy (1 locality, 395 m); Zulia (8 localities, 24-1,135 m). | Apure, Barinas, Bolívar, Carabobo, Dto. Federal, Falcon, Guarico, Lara, Miranda, Monagas, Amazonas, Trujillo, Yaracuy y Zulia | Venezuela | 1960 | 1967 |
|  | *Artibeus lituratus* | 1 | El Dorado | Bolívar | Venezuela | 2/08/1962 |  |
|  | *Artibeus lituratus* | 2 | Reserva Natural La Palmita | Casanare | Colombia | 2017 |  |
|  | *Artibeus planirostris* | 1 | Tame | Arauca | Colombia | 2018 | 2019 |
| *Neotrichobius bisetosus* | *Artibeus obscurus* | 2 | El Manteco, Río Supamo | Bolívar | Venezuela | 7/04/1966 | 11/04/1966 |
|  | *Artibeus obscurus* | 9 | Esmeralda, Río Mavaca | Amazonas | Venezuela | 3/04/1967 | 12/04/1967 |
|  | *Artibeus planirostris* | 2 | Esmeralda, Río Mavaca | Amazonas | Venezuela | 3/04/1967 | 12/04/1967 |
|  | *Artibeus obscurus* | 10 | Esmeralda, Caño Culebra, Belén | Amazonas | Venezuela | 1/02/1967 | 6/02/1967 |
|  | *Artibeus obscurus* | 6 | Esmeralda, Caño Essa, Belén | Amazonas | Venezuela | 7/02/1967 |  |
|  | *Artibeus obscurus* | 16 | Esmeralda, Río Cunucunuma, Belén | Amazonas | Venezuela | 2/01/1967 | 12/02/1967 |
|  | *Artibeus obscurus* | 1 | Esmeralda, Río Mavaca | Amazonas | Venezuela | 3/03/1967 |  |
|  | *Artibeus obscurus* | 10 | Esmeralda, Brazo Casiquiare, Capibara | Amazonas | Venezuela | 30/05/1967 | 1/04/1967 |
|  | *Artibeus obscurus* | 3 | Pto. Ayacucho, Río Manapiare, San Juan | Amazonas | Venezuela | 14/07/1967 |  |
|  | *Artibeus obscurus* | 3 | Río Orinoco, Tamatama | Amazonas | Venezuela | 25/04/1967 | 15/05/1967 |
| *Neotrichobius delicatus* | *Vampyressa thyone* | 6 | Altamira, Altamira | Barinas | Venezuela | 1/01/1968 | 5/01/1968 |
|  | *Vampyressa thyone* | 2 | Altamira | Barinas | Venezuela | 21/07/1967 | 11/01/1968 |
|  | *Vampyressa thyone* | 15 | Montalbán, La Copa | Carabobo | Venezuela | 27/11/1967 | 1/12/1967 |
|  | *Vampyressa thyone* | 9 | Montalbán, Cumbre Canoabo | Carabobo | Venezuela | 1/11/1967 |  |
|  | *Vampyressa thyone* | 3 | San Felipe, Minas de Aroa | Yaracuy | Venezuela | 9/07/1967 | 22/07/1967 |
| *Neotrichobius delicatus s.l.* | *Dermanura cinerea* | 11 | Santo Domingo, Selvas de San Camilo, Nulita | Apure | Venezuela | 17/01/1968 | 5/02/1968 |
|  | *Dermanura cinerea* | 2 | Altamira, Altamira | Barinas | Venezuela | 1/01/1968 | 5/01/1968 |
|  | *Platyrrhinus helleri* | 1 | Altamira, Altamira | Barinas | Venezuela | 1/01/1968 | 5/01/1968 |
|  | *Rhinophylla pumilio* | 2 | El Dorado | Bolívar | Venezuela | 8/06/1966 | 25/06/1966 |
|  | *Dermanura cinerea* | 3 | El Dorado | Bolívar | Venezuela | 8/06/1966 | 25/06/1966 |
|  | *Dermanura cinerea* | 20 | El Dorado | Bolívar | Venezuela | 9/05/1966 | 19/05/1966 |
|  | *Rhinophylla pumilio* | 9 | El Dorado | Bolívar | Venezuela | 9/05/1966 | 19/05/1966 |
|  | *Rhinophylla pumilio* | 1 | El Manteco, Río Supamo | Bolívar | Venezuela | 10/04/1966 |  |
|  | *Dermanura cinerea* | 1 | La Paragua, Hato San José | Bolívar | Venezuela | 20/03/1967 |  |
|  | *Dermanura cinerea* | 2 | Icabarú, El Pauji, Icabarú | Bolívar | Venezuela | 27/04/1968 |  |
|  | *Dermanura cinerea* | 1 | Icabarú, Santa Lucia de Surukun | Bolívar | Venezuela | 2/05/1968 |  |
|  | *Dermanura cinerea* | 26 | Montalbán, La Copa | Carabobo | Venezuela | 27/11/1967 | 1/12/1967 |
|  | *Dermanura cinerea* | 2 | Montalbán, Cumbre Canoabo | Carabobo | Venezuela | 1/11/1967 |  |
|  | *Dermanura cinerea* | 2 | Altagracia, Hda. El Vira | Guárico | Venezuela | 16/09/1966 |  |
|  | *Dermanura cinerea* | 1 | Esmeralda, Brazo Casiquiare, Capibara | Amazonas | Venezuela | 29/05/1967 | 2/06/1967 |
|  | *Rhinophylla pumilio* | 4 | Esmeralda, Brazo Casiquiare, Capibara | Amazonas | Venezuela | 29/05/1967 | 2/06/1967 |
|  | *Rhinophylla pumilio* | 4 | Pto. Ayacucho, nr. Morganito | Amazonas | Venezuela | 4/10/1967 | 09-101967 |
|  | *Artibeus* sp. | 1 | Pto. Ayacucho, nr. Morganito | Amazonas | Venezuela | 4/10/1967 | 09-101967 |
|  | *Uroderma magnirostrum* | 1 | Pto. Ayacucho, Río Manapiare, San Juan | Amazonas | Venezuela | 12/07/1967 |  |
|  | *Dermanura cinerea* | 2 | Esmeralda, Cerro Duida | Amazonas | Venezuela | 19/01/1967 |  |
|  | *Artibeus planirostris* | 1 | Esmeralda, Río Cunucunuma, Belén | Amazonas | Venezuela | 4/04/1967 |  |
|  | *Artibeus* sp. | 1 | Esmeralda, Río Cunucunuma, Belén | Amazonas | Venezuela | 3/01/1967 |  |
|  | *Artibeus* sp. | 6 | Esmeralda, Río Mavaca | Amazonas | Venezuela | 13/04/1967 |  |
| *Neotrichobius ectophyllae* | *Mesoophylla macconelli* | 6 | Esmeralda, Brazo Casiquiare, Capibara | Amazonas | Venezuela | 29/05/1967 | 01-04-11967 |
| *Neotrichobius stenopterus* | *Dermanura cinerea* | 1 | Trujillo | Trujillo | Venezuela |  |  |
| *Noctiliostrebla maai* | *Molossus rufus* | 1 | Pto. Páez, Río Cinaruco, Hato Cariben | Apure | Venezuela | 14/07/1965 | 28/07/1965 |
|  | *Noctilio albiventris* | 26 | Pto. Páez, Río Cinaruco, Hato Cariben | Apure | Venezuela | 14/07/1965 | 28/07/1965 |
|  | *Noctilio albiventris* | 24 | Pto. Páez, La Villa, Hato Cariben | Apure | Venezuela | 6/07/1965 | 28/07/1965 |
|  | *Noctilio albiventris* | 4 | Pto. Páez | Apure | Venezuela | 17/01/1966 |  |
|  | *Noctilio albiventris* | 9 | Maturín, Hato Mata de Bejuco | Monagas | Venezuela | 4/06/1968 |  |
|  | *Noctilio albiventris* | 1 | Pto. Ayacucho, Las Queseras | Amazonas | Venezuela | 24/09/1967 |  |
|  | *Noctilio albiventris* | 10 | Pto. Ayacucho, Guayabal | Amazonas | Venezuela | 7/10/1967 |  |
|  | *Noctilio albiventris* | 16 | Pto. Ayacucho, Río Manapiare, San Juan | Amazonas | Venezuela | 14/07/1967 | 24/07/1967 |
|  | *Noctilio albiventris* | 12 | Urama, El Central | Yaracuy | Venezuela | 14/03/1966 | 15/03/1966 |
|  | *Noctilio albiventris* | 76 | Urama, El Central | Yaracuy | Venezuela | 8/03/1966 | 14/03/1966 |
|  | *Noctilio albiventris* | 2 | Playa del Medio | Bolívar | Venezuela | 19/04/1961 |  |
|  | *Noctilio albiventris* | 87 | Arauca y Cravo Norte | Arauca | Colombia | 12/11/2018 | 26/11/2018 |
|  | *Sturnira giannae* | 7 | Tame, Vda. Santa Inés | Arauca | Colombia | 6/03/2019 |  |
| *Noctiliostrebla aitkeni* | *Noctilio leporinus* | 28 | El Manteco, Río Supamo | Bolívar | Venezuela | 8/04/1966 | 11/04/1966 |
|  | *Noctilio leporinus* | 2 | Maturín, Hato Mata de Bejuco | Monagas | Venezuela | 3/06/1968 |  |
|  | *Saccopteryx bilineata* | 1 | Esmeralda, Río Cunucunuma, Belén | Amazonas | Venezuela | 12/01/1967 |  |
|  | *Noctilio leporinus* | 5 | Esmeralda, Caño Culebra, Belén | Amazonas | Venezuela | 12/01/1967 | 2/02/1967 |
|  | *Noctilio leporinus* | 15 | Esmeralda, Río Mavaca | Amazonas | Venezuela | 20/03/1967 |  |
|  | *Noctilio leporinus* | 9 | Esmeralda, Río Mavaca | Amazonas | Venezuela | 1/03/1967 |  |
|  | *Noctilio leporinus* | 15 | Esmeralda, Río Mavaca | Amazonas | Venezuela | 10/03/1967 |  |
|  | *Noctilio leporinus* | 8 | Esmeralda, Río Mavaca | Amazonas | Venezuela | 5/04/1967 |  |
|  | *Noctilio leporinus* | 1 | Urama, El Central | Yaracuy | Venezuela | 14/03/1966 | 15/03/1966 |
| *Noctiliostrebla traubi* | *Noctilio leporinus* | 1 | San Juan de los Morros, Hto. Las Palmitas | Guárico | Venezuela | 7/01/1968 |  |
|  | *Noctilio leporinus* | 115 | Urama, El Central | Yaracuy | Venezuela | 8/03/1966 | 14/03/1966 |
|  | *Noctilio leporinus* | 1 | Urama, El Central | Yaracuy | Venezuela | 8/03/1966 | 14/03/1966 |
| *Paradyschiria parvula* | *Noctilio albiventris* | 1 | Pto. Páez | Apure | Venezuela | 17/01/1966 |  |
|  | *Noctilio albiventris* | 14 | Maturín, Hato Mata de Bejuco | Monagas | Venezuela | 4/06/1968 |  |
|  | *Molossus aztecus* | 1 | Pto. Ayacucho, Río Manapiare, San Juan | Amazonas | Venezuela | 14/07/1967 | 24/07/1967 |
|  | *Molossus rufus* | 3 | Pto. Ayacucho, Río Manapiare, San Juan | Amazonas | Venezuela | 14/07/1967 | 24/07/1967 |
|  | *Noctilio albiventris* | 298 | Pto. Ayacucho, Río Manapiare, San Juan | Amazonas | Venezuela | 14/07/1967 | 24/07/1967 |
|  | *Noctilio albiventris* | 1 | Pto. Ayacucho, Las Queseras | Amazonas | Venezuela | 24/09/1967 |  |
|  | *Noctilio albiventris* | 185 | Urama, El Central | Yaracuy | Venezuela | 8/03/1966 | 14/03/1966 |
|  | *Noctilio albiventris* | 62 | Urama, El Central | Yaracuy | Venezuela | 15/03/1966 |  |
| *Paradyschiria parvuloides* | *Noctilio albiventris* | 1 | Pto. Páez | Apure | Venezuela | 17/01/1966 |  |
|  | *Noctilio albiventris* | 21 | Valera, La Ceiba | Trujillo | Venezuela | 19/02/1966 |  |
|  | *Noctilio albiventris* | 113 | Arauca | Arauca | Colombia |  |  |
|  | *Cynomops planirostris* | 1 | Arauca, Los Trompillos | Arauca | Colombia | 3/11/2021 |  |
| *Paradyschiria curvata* | *Noctilio albiventris* | 213 | Pto. Páez, Río Cinaruco, Hato Cariben | Apure | Venezuela | 14/07/1967 | 28/07/1965 |
|  | *Noctilio albiventris* | 6 | Pto. Páez, La Villa, Hato Cariben | Apure | Venezuela | 24/07/1965 |  |
|  | *Noctilio albiventris* | 11 | Pto. Paez | Apure | Venezuela | 17/01/1966 |  |
|  | *Trachops cirrhosus* | 1 | Pto. Páez, La Villa, Hato Cariben | Apure | Venezuela | 24/07/1965 |  |
|  | *Molossus rufus* | 2 | Pto. Páez | Apure | Venezuela | 17/01/1966 |  |
|  | *Desmodus rotundus* | 1 | Pto. Páez, Río Cinaruco, Hato Cariben | Apure | Venezuela | 14/07/1967 | 28/07/1965 |
|  | *Molossus rufus* | 1 | Pto. Páez, Río Cinaruco, Hato Cariben | Apure | Venezuela | 14/07/1967 | 28/07/1965 |
|  | *Noctilio albiventris* | 2 | Pto. Páez, Río Cinaruco, Hato Cariben | Apure | Venezuela | 14/07/1967 | 28/07/1965 |
| *Paradyschiria fusca* | *Noctilio leporinus* | 1 | Caicara, Hato La Florida | Bolívar | Venezuela | 4/05/1967 |  |
|  | *Noctilio leporinus* | 82 | El Manteco, Río Supamo | Bolívar | Venezuela | 8/04/1966 | 10/04/1966 |
|  | *Noctilio leporinus* | 18 | Maturín, Hato Mata de Bejuco | Monagas | Venezuela | 3/06/1968 |  |
|  | *Noctilio leporinus* | 52 | Esmeralda, Caño Culebra, Belén | Amazonas | Venezuela | 12/01/1967 | 2/02/1967 |
|  | *Noctilio leporinus* | 10 | Esmeralda, Río Mavaca | Amazonas | Venezuela | 20/03/1967 |  |
|  | *Noctilio leporinus* | 2 | Esmeralda, Río Mavaca | Amazonas | Venezuela | 2/03/1967 |  |
|  | *Noctilio leporinus* | 3 | Esmeralda, Río Mavaca | Amazonas | Venezuela | 10/03/1967 |  |
|  | *Noctilio leporinus* | 1 | Esmeralda, Río Mavaca | Amazonas | Venezuela | 5/04/1967 |  |
| *Paradyschiria lineata* | *Noctilio leporinus* | 7 | San Juan de los Morros, Hato. Las Palmitas | Guárico | Venezuela | 4/09/1966 |  |
|  | *Noctilio albiventris* | 1 | Urama, El Central | Yaracuy | Venezuela | 8/03/1966 | 14/03/1966 |
|  | *Noctilio leporinus* | 246 | Urama, El Central | Yaracuy | Venezuela | 8/03/1966 | 14/03/1966 |
|  | *Noctilio leporinus* | 4 | Urama, El Central | Yaracuy | Venezuela | 14/03/1966 |  |
|  | *Pteronotus parnellii* | 1 | Urama, El Central | Yaracuy | Venezuela | 14/03/1966 |  |
| *Parastrebla handleyi* | *Trinycteris nicefori* | 12 | El Manteco, Los Patos | Bolívar | Venezuela | 5/04/1966 |  |
| *Paratrichobius dunni* | *Uroderma magnirostrum* | 1 | Santo Domingo, Selvas de San Camilo, Nulita | Apure | Venezuela | 29/01/1968 | 6/02/1968 |
|  | *Uroderma bilobatum* | 7 | Santo Domingo, Selvas de San Camilo, Nulita | Apure | Venezuela | 29/01/1968 | 6/02/1968 |
|  | *Uroderma bilobatum* | 19 | Altamira, Altamira | Barinas | Venezuela | 26/07/1967 |  |
|  | *Uroderma bilobatum* | 1 | Altamira, Altamira | Barinas | Venezuela | 26/07/1967 |  |
|  | *Uroderma bilobatum* | 3 | El Dorado, El Manaco | Bolívar | Venezuela | 13/06/1966 | 20/06/1966 |
|  | *Uroderma bilobatum* | 3 | Icabarú, Santa Lucia de Surukun | Bolívar | Venezuela | 29/04/1968 |  |
|  | *Desmodus rotundus* | 1 | Pto. Ayacucho, Río Manapiare, San Juan | Amazonas | Venezuela | 11/07/1967 | 27/07/1967 |
|  | *Uroderma magnirostrum* | 4 | Pto. Ayacucho, Río Manapiare, San Juan | Amazonas | Venezuela | 11/07/1967 | 27/07/1967 |
|  | *Uroderma bilobatum* | 2 | Pto. Ayacucho, Río Manapiare, San Juan | Amazonas | Venezuela | 11/07/1967 | 27/07/1967 |
|  | *Uroderma magnirostrum* | 1 | Río Orinoco, Tamatama | Amazonas | Venezuela | 28/04/1967 |  |
|  | *Uroderma bilobatum* | 4 | Esmeralda, Río Cunucunuma, Belén | Amazonas | Venezuela | 6/01/1967 | 3/02/1967 |
|  | *Uroderma bilobatum* | 1 | Pto. Ayacucho, nr. Morganito | Amazonas | Venezuela | 8/10/1967 |  |
|  | *Uroderma bilobatum* | 2 | Esmeralda, Río Mavaca | Amazonas | Venezuela | 14/04/1967 |  |
|  | *Uroderma bilobatum* | 2 | Valera, La Ceiba | Trujillo | Venezuela | 28/10/1967 |  |
|  | *Uroderma bilobatum* | 1 | Valera, nr. Agua Viva | Trujillo | Venezuela | 6/10/1965 |  |
|  | *Uroderma bilobatum* | 3 | Urama, El Central | Yaracuy | Venezuela | 14/03/1966 | 21/03/1966 |
|  | *Uroderma bilobatum* | 3 | Urama, El Central | Yaracuy | Venezuela | 22/03/1966 |  |
| *Paratrichobius longicrus* | *Artibeus lituratus* | 46 | Santo Domingo, Selvas de San Camilo, Nulita | Apure | Venezuela | 17/01/1968 | 5/02/1968 |
|  | *Artibeus lituratus* | 4 | Altamira | Barinas | Venezuela | 9/01/1968 | 11/01/1968 |
|  | *Artibeus lituratus* | 4 | El Dorado, El Manaco | Bolívar | Venezuela | 9/06/1966 | 23/06/1966 |
|  | *Artibeus lituratus* | 2 | La Paragua, Hato San José | Bolívar | Venezuela | 6/03/1967 | 6/04/1967 |
|  | *Artibeus lituratus* | 4 | El Dorado | Bolívar | Venezuela | 16/05/1966 | 17/05/1966 |
|  | *Artibeus lituratus* | 1 | Pto. Ayacucho, Pto. Ayacucho | Amazonas | Venezuela | 13/10/1967 |  |
|  | *Artibeus lituratus* | 1 | Pto. Ayacucho, Raya, Pto. Ayacucho | Amazonas | Venezuela | 9/10/1967 |  |
|  | *Artibeus lituratus* | 1 | Pto. Ayacucho, Río Manapiare, San Juan | Amazonas | Venezuela | 12/07/1967 |  |
|  | *Artibeus lituratus* | 4 | Río Orinoco, Tamatama | Amazonas | Venezuela | 8/05/1967 | 9/05/1967 |
|  | *Artibeus lituratus* | 2 | Montalbán, Potrerito | Carabobo | Venezuela | 1/11/1967 |  |
|  | *Artibeus lituratus* | 3 | Montalbán, La Copa | Carabobo | Venezuela | 28/11/1967 | 30/11/1967 |
|  | *Artibeus lituratus* | 2 | Valera, La Ceiba | Trujillo | Venezuela | 5/11/1965 |  |
|  | *Artibeus lituratus* | 1 | Valera, nr. Sabana de Mendoza | Trujillo | Venezuela | 27/08/1965 |  |
|  | *Artibeus lituratus* | 1 | Valera, nr. Agua Viva | Trujillo | Venezuela | 23/10/1965 |  |
|  | *Artibeus lituratus* | 4 | Valera, nr. Agua Santa | Trujillo | Venezuela | 15/08/1965 |  |
|  | *Artibeus lituratus* | 1 | San Felipe, Minas de Aroa | Yaracuy | Venezuela | 14/07/1967 |  |
|  | *Artibeus lituratus* | 1 | Rancho Grande, El Limón | Aragua | Venezuela | 30/03/1960 |  |
| *Paratrichobius longicrus s.l.* | *Desmodus rotundus* | 1 | Santo Domingo, Selvas de San Camilo, Nulita | Apure | Venezuela | 24/01/1968 | 31/01/1968 |
|  | *Carollia perspicillata* | 1 | Santo Domingo, Selvas de San Camilo, Nulita | Apure | Venezuela | 24/01/1968 | 31/01/1968 |
|  | *Platyrrhinus vittatus* | 1 | Altamira, Altamira | Barinas | Venezuela | 5/01/1968 |  |
|  | *Platyrrhinus aurarinus* | 28 | El Dorado | Bolívar | Venezuela | 23/03/1966 | 26/05/1966 |
|  | *Platyrrhinus umbratus* | 1 | Caripe, San Agustín | Monagas | Venezuela | 1/07/1967 |  |
|  | *Platyrrhinus umbratus* | 1 | Caripe, San Agustín | Monagas | Venezuela | 14/07/1967 |  |
|  | *Platyrrhinus aurarinus* | 1 | Esmeralda, Cerro Duida | Amazonas | Venezuela | 17/01/1967 |  |
| *Paratrichobius lowei* | *Dermanura cinerea* | 12 | El Dorado | Bolívar | Venezuela | 12/05/1966 | 19/05/1966 |
| *Paratrichobius sanchezi* | *Euchistenes hartii* | 1 | Altagracia, Hda. El Vira | Guárico | Venezuela | 16/09/1966 |  |
|  | *Euchistenes hartii* | 2 | Montalbán, La Copa | Carabobo | Venezuela | 6/08/1968 | 7/08/1968 |
|  | *Euchistenes hartii* | 1 | Caripe, San Agustín | Monagas | Venezuela | 6/07/1967 |  |
| *Paratrichobius salvini* | *Chiroderma salvini* | 1 | Montalbán, La Copa | Carabobo | Venezuela | 29/11/1967 |  |
|  | *Chiroderma salvini* | 1 | Caripe, San Agustín | Monagas | Venezuela | 28/06/1967 |  |
| *Paratrichobius salvini s.l.* | *Platyrrhinus helleri* | 4 | Santo Domingo, Selvas de San Camilo, Nulita | Apure | Venezuela | 17/01/1968 |  |
|  | *Chiroderma villosum* | 1 | Santo Domingo, Selvas de San Camilo, Nulita | Apure | Venezuela | 6/02/1968 |  |
|  | *Platyrrhinus helleri* | 25 | Altamira, Altamira | Barinas | Venezuela | 31/07/1967 | 2/02/1968 |
|  | *Platyrrhinus helleri* | 1 | Altamira | Barinas | Venezuela | 19/07/1967 | 21/07/1967 |
|  | *Chiroderma trinitatum* | 1 | Altamira | Barinas | Venezuela | 19/07/1967 | 21/07/1967 |
|  | *Platyrrhinus helleri* | 3 | Montalbán | Carabobo | Venezuela | 27/11/1967 | 29/11/1967 |
|  | *Platyrrhinus helleri* | 2 | Pto. Ayacucho, Río Manapiare, San Juan | Amazonas | Venezuela | 6/07/1967 | 27/07/1967 |
|  | *Chiroderma villosum* | 2 | Pto. Ayacucho, Río Manapiare, San Juan | Amazonas | Venezuela | 6/07/1967 | 27/07/1967 |
|  | *Vampyrodes caraccioli* | 1 | Esmeralda, Caño Essa, Belén | Amazonas | Venezuela | 14/11/1967 |  |
|  | *Vampyriscus bidens* | 1 | Pto. Ayacucho, Las Queseras | Amazonas | Venezuela | 24/09/1967 |  |
|  | *Vampyriscus bidens* | 4 | Pto. Ayacucho, nr. Morganito | Amazonas | Venezuela | 4/10/1967 | 8/10/1967 |
|  | *Chiroderma villosum* | 1 | Pto. Ayacucho, Chaparito | Amazonas | Venezuela | 2/10/1967 |  |
|  | *Platyrrhinus helleri* | 19 | San Felipe, Minas de Aroa | Yaracuy | Venezuela | 6/07/1966 | 22/07/1966 |
|  | *Chiroderma villosum* | 10 | San Felipe, Minas de Aroa | Yaracuy | Venezuela | 6/07/1966 | 22/07/1966 |
|  | *Chiroderma villosum* | 1 | Urama, El Central | Yaracuy | Venezuela | 22/03/1966 |  |
| *Pseudostrebla greenwelli* | *Lophostoma brasiliense* | 1 | Pto. Ayacucho, Río Manapiare, San Juan | Amazonas | Venezuela | 27/07/1967 |  |
|  | *Tonatia maresi* | 4 | Reserva Natural La Palmita | Casanare | Colombia | 2017 |  |
| *Pseudostrebla ribeiroi* | *Lophostoma silvicola* | 2 | Esmeralda, Brazo Casiquiare, Capibara | Amazonas | Venezuela | 30/05/1967 | 12/06/1967 |
|  | *Lophostoma silvicola* | 1 | Pto. Ayacucho, nr. Morganito | Amazonas | Venezuela | 9/10/1967 |  |
| *Pseudostrebla sparsisetis* | *Lophostoma carrikeri* | 2 | Esmeralda, Río Mavaca | Amazonas | Venezuela | 10/04/1967 |  |
|  | *Lophostoma carrikeri* | 2 | Los Micos, San Juan de Aroma | Meta | Colombia | 16/04/1957 |  |
|  | *Lophostoma carrikeri* | 4 | Los Micos, San Juan de Aroma | Meta | Colombia | 16/04/1957 |  |
| *Speiseria ambigua* | *Carollia perspicillata* | 277 | Barinas (2 localities, 619-794 m); Bolivar (8 localities, 150-916 m); Carabobo (5 localities, 25-1,537 m); Falcon (5 localities, 2-250 m); Guarico (2 localities, 470-630 m); Miranda (5 locaUties, 1-1,160 m); Monagas (3 localities, 854-1,170 m); Amazonas (9 localiHes, 114-161 m); Trujillo (3 localities, 90-164 m); Yaracuy (2 localities, 25-37 m); Zulia (9 localities, 37-270 m). | Barinas, Bolívar, Carabobo, Falcon, Guarico, Lara, Miranda, Monagas, Amazonas, Trujillo, Yaracuy y Zulia | Venezuela | 1960 | 1967 |
|  | *Carollia perspicillata* | 5 | Reserva Natural La Palmita | Casanare | Venezuela | 2017 |  |
|  | *Carollia brevicauda* | 1 | Tame | Arauca | Colombia | 2018 | 2019 |
| *Speiseria magnioculus* | *Trachops cirrhosus* | 2 | El Dorado, El Manaco | Bolívar | Venezuela | 14/06/1966 |  |
|  | *Trachops cirrhosus* | 2 | La Paragua, Hato San José | Bolívar | Venezuela | 6/03/1967 | 10/04/1967 |
|  | *Trachops cirrhosus* | 1 | Esmeralda, Brazo Casiquiare, Capibara | Amazonas | Venezuela | 8/07/1967 |  |
|  | *Trachops cirrhosus* | 13 | Pto. Ayacucho, Río Manapiare, San Juan | Amazonas | Venezuela | 5/07/1967 | 27/07/1967 |
|  | *Trachops cirrhosus* | 38 | Esmeralda, Río Mavaca | Amazonas | Venezuela | 3/04/1967 | 14/04/1967 |
| *Speseria peytoni* | *Carollia brevicauda* | 1 | Santo Domingo, Selvas de San Camilo, Nulita | Apure | Venezuela | 1960 | 1967 |
|  | *Carollia brevicauda* | 6 | Altamira, Alramira | Barinas | Venezuela | 1960 | 1967 |
|  | *Carollia brevicauda* | 32 | Altamira, Alramira | Barinas | Venezuela | 1960 | 1967 |
|  | *Carollia brevicauda* | 2 | Altamira, Alramira | Barinas | Venezuela | 1960 | 1967 |
|  | *Carollia brevicauda* | 4 | Altamira, Alramira | Barinas | Venezuela | 1960 | 1967 |
|  | *Carollia brevicauda* | 19 | Altamira, Alramira | Barinas | Venezuela | 1960 | 1967 |
|  | *Carollia brevicauda* | 2 | Montalbán, La Voluntad | Carabobo | Venezuela | 1960 | 1967 |
|  | *Carollia brevicauda* | 5 | Montalbán, Hda. La Canada | Carabobo | Venezuela | 1960 | 1967 |
|  | *Carollia brevicauda* | 1 | Montalbán, La Leonera | Carabobo | Venezuela | 1960 | 1967 |
|  | *Carollia brevicauda* | 4 | Montalbán, Cumbre Canoabo | Carabobo | Venezuela | 1960 | 1967 |
|  | *Carollia brevicauda* | 9 | Montalbán, Cumbre Canoabo | Carabobo | Venezuela | 1960 | 1967 |
|  | *Carollia brevicauda* | 1 | El Dorado | Bolívar | Venezuela | 1960 | 1967 |
| *Stizostrebla longirostris* | *Lophostoma carrikeri* | 4 | Esmeralda, Río Mavaca | Amazonas | Venezuela | 10/04/1967 |  |
| *Trichobius anducei* | *Carollia perspicillata* | 3 | Arauca | Arauca | Venezuela | 2018 | 2019 |
| *Trichobius pallidus* | *Furipterus horrens* | 1 | Río Orinoco, Tamatama | Amazonas | Venezuela | 20/04/1967 |  |
| *Trichobius caecus* | *Trachops cirrhosus* | 3 | Pto.Páez, La Villa, Hato Cariben | Apure | Venezuela | 24-071965 | 28/07/1965 |
|  | *Macrophyllum macrophyllum* | 2 | Pto.Páez, La Villa, Hato Cariben | Apure | Venezuela | 24-071965 | 28/07/1965 |
|  | *Pteronotus parnellii* | 18 | Pto.Páez, La Villa, Hato Cariben | Apure | Venezuela | 24-071965 | 28/07/1965 |
|  | *Pteronotus parnellii* | 10 | Pto. Páez, Cerro de Murcielagos, Pto. Páez | Apure | Venezuela | 19/01/1966 |  |
|  | *Desmodus rotundus* | 1 | Caripe | Monagas | Venezuela | 13/07/1967 |  |
|  | *Anoura geoffroyi* | 8 | La Paragua, Hato San José | Bolívar | Venezuela | 10/04/1967 |  |
|  | *Pteronotus parnellii* | 35 | La Paragua, Hato San José | Bolívar | Venezuela | 10/04/1967 |  |
|  | *Artibeus obscurus* | 3 | El Manteco, Los Patos | Bolívar | Venezuela | 11/04/1966 |  |
|  | *Artibeus lituratus* | 1 | El Manteco, Los Patos | Bolívar | Venezuela | 11/04/1966 |  |
|  | *Pteronotus parnellii* | 52 | El Manteco, Los Patos | Bolívar | Venezuela | 11/04/1966 |  |
|  | *Pteronotus parnellii* | 5 | El Dorado, Km 67, El Manaco | Bolívar | Venezuela | 16/04/1966 |  |
|  | *Pteronotus parnellii* | 174 | Guasipati, Guasipati | Bolívar | Venezuela | 29/04/1966 |  |
|  | *Pteronotus parnellii* | 625 | Caicara, Hato La Florida | Bolívar | Venezuela | 19/04/1967 | 5/05/1967 |
|  | *Pteronotus parnellii* | 1 | Icabarú, Icabarú | Bolívar | Venezuela | 11/05/1968 |  |
|  | *Pteronotus parnellii* | 8 | El Dorado km 125 | Bolívar | Venezuela | 11/05/1966 | 16/05/1966 |
|  | *Pteronotus parnellii* | 22 | El Manteco, Río Supamo | Bolívar | Venezuela | 10/04/1966 |  |
|  | *Pteronotus parnellii* | 16 | Altagracia, Hda. El Vira | Guárico | Venezuela | 16/09/1966 |  |
|  | *Uroderma bilobatum* | 2 | Esmeralda, Río Cunucunuma, Belén | Amazonas | Venezuela | 10/01/1967 | 9/02/1967 |
|  | *Pteronotus parnellii* | 12 | Esmeralda, Río Cunucunuma, Belén | Amazonas | Venezuela | 10/01/1967 | 9/02/1967 |
|  | *Rhynchonycteris naso* | 1 | Esmeralda, Río Mavaca | Amazonas | Venezuela | 3/04/1967 | 11/04/1967 |
|  | *Pteronotus parnellii* | 150 | Esmeralda, Río Mavaca | Amazonas | Venezuela | 3/04/1967 | 11/04/1967 |
|  | *Desmodus rotundus* | 2 | Río Orinoco, Tamatama | Amazonas | Venezuela | 27/04/1967 | 7/05/1967 |
|  | *Pteronotus parnellii* | 19 | Río Orinoco, Tamatama | Amazonas | Venezuela | 27/04/1967 | 7/05/1967 |
|  | *Pteronotus parnellii* | 7 | Esmeralda, Caño Culebra, Belén | Amazonas | Venezuela | 3/02/1967 |  |
|  | *Pteronotus parnellii* | 72 | Esmeralda, Boca Mavaca | Amazonas | Venezuela | 17/03/1967 | 24/03/1967 |
|  | *Pteronotus parnellii* | 10 | Pto. Ayacucho. Raya. Pto. Ayacucho | Amazonas | Venezuela | 7/09/1967 | 2/10/1967 |
|  | *Pteronotus parnellii* | 35 | Pto. Ayacucho, Río Manapiare, San Juan | Amazonas | Venezuela | 5/07/1967 | 27/07/1967 |
|  | *Pteronotus davyi* | 1 | San Felipe, Minas de Aroa | Yaracuy | Venezuela | 6/07/1967 | 19/07/1967 |
|  | *Pteronotus parnellii* | 60 | San Felipe, Minas de Aroa | Yaracuy | Venezuela | 6/07/1967 | 19/07/1967 |
|  | *Pteronotus parnellii* | 3 | Urama, El Central, Urama | Yaracuy | Venezuela | 8/03/1966 |  |
|  | *Pteronotus parnellii* | 18 | Urama, El Central, Urama | Yaracuy | Venezuela | 14/03/1966 | 15/03/1966 |
|  | *Pteronotus parnellii* | 1 | Montalbán | Carabobo | Venezuela | 29/11/1967 |  |
|  | *Pteronotus parnellii* | 10 | Rancho Grande, El Limón | Aragua | Venezuela |  |  |
|  | *Pteronotus parnellii* | 19 | Rancho Grande, El Limón | Aragua | Venezuela | 5/08/1965 |  |
|  | *Myotis keaysi* | 1 | Maracay | Aragua | Venezuela | 5/08/1965 |  |
|  | *Pteronotus rubiginosus* | 5 | Serranía de Nuria | Bolívar | Venezuela | 31/07/1962 |  |
| *Trichobius johnsonae* | *Pteronotus personatus* | 2 | El Manteco, Los Patos | Bolívar | Venezuela | 11/04/1966 |  |
|  | *Pteronotus davyi* | 11 | San Felipe, Minas de Aroa | Yaracuy | Venezuela | 7/07/1967 | 23/07/1967 |
|  | *Pteronotus personatus* | 1 | San Felipe, Minas de Aroa | Yaracuy | Venezuela | 7/07/1967 | 23/07/1967 |
|  | *Pteronotus gymnonotus* | 20 | San Felipe, Minas de Aroa | Yaracuy | Venezuela | 7/07/1967 | 23/07/1967 |
|  | *Noctilio albiventris* | 1 | El Central, Urama | Yaracuy | Venezuela | 8/03/1966 |  |
|  | *Pteronotus gymnonotus* | 5 | Rancho Grande, El Limón | Aragua | Venezuela | 30/03/1960 |  |
| *Trichobius bilobus* | *Pteronotus gymnonotus* | 3 | Valera, nr. Agua Viva | Trujillo | Venezuela | 14/09/1965 |  |
|  | *Pteronotus gymnonotus* | 1 | San Felipe, Minas de Aroa | Yaracuy | Venezuela | 16/07/1967 |  |
| *Trichobius galei* | *Natalus tumidirostris* | 30 | Caicara, Hato La Florida | Bolívar | Venezuela | 19/04/1967 | 24/04/1967 |
|  | *Natalus tumidirostris* | 2 | Rancho Grande | Aragua | Venezuela | 17/08/1949 | 19/08/1949 |
| *Trichobius sparsus* | *Natalus tumidirostris* | 1 | Caicara, Hato La Florida | Bolívar | Venezuela | 19/04/1967 | 24/04/1967 |
|  | *Pteronotus parnellii* | 24 | Caicara, Hato La Florida | Bolívar | Venezuela | 19/04/1967 | 24/04/1967 |
|  | *Pteronotus parnellii* | 5 | El Dorado, El Manaco | Bolívar | Venezuela | 16/04/1966 |  |
|  | *Pteronotus parnellii* | 9 | El Dorado, El Manaco | Bolívar | Venezuela | 13/06/1966 | 23/03/1966 |
|  | *Pteronotus parnellii* | 3 | Guasipati, Guasipati | Bolívar | Venezuela | 29/04/1966 |  |
|  | *Pteronotus parnellii* | 16 | La Paragua, Hato San José | Bolívar | Venezuela | 10/04/1967 |  |
|  | *Pteronotus parnellii* | 1 | El Dorado | Bolívar | Venezuela | 11/05/1966 |  |
|  | *Pteronotus parnellii* | 18 | El Manteco, Los Patos | Bolívar | Venezuela | 11/04/1966 |  |
|  | *Pteronotus parnellii* | 6 | El Manteco, Río Supamo | Bolívar | Venezuela | 8/04/1966 | 10/04/1966 |
|  | *Pteronotus parnellii* | 1 | Esmeralda, Boca Mavaca | Amazonas | Venezuela | 17/03/1967 |  |
|  | *Pteronotus parnellii* | 1 | Esmeralda, Boca Mavaca | Amazonas | Venezuela | 3/04/1967 |  |
|  | *Pteronotus parnellii* | 13 | Pto. Ayacucho, Río Manapiare, San Juan | Amazonas | Venezuela | 5/07/1967 | 25/07/1967 |
|  | *Pteronotus parnellii* | 1 | Altagracia, Hda. El Vira | Guárico | Venezuela |  |  |
| *Trichobius parasparsus* | *Pteronotus parnellii* | 4 | Pto. Páez, La Villa, Hato Cariben | Apure | Venezuela | 24/07/1965 | 28/07/1965 |
|  | *Pteronotus parnellii* | 2 | Pto. Páez, Cerro de Murcielagos, Pto. Páez | Apure | Venezuela | 19/01/1966 |  |
|  | *Pteronotus parnellii* | 4 | El Dorado | Bolívar | Venezuela | 16/06/1966 |  |
|  | *Pteronotus parnellii* | 4 | El Dorado, El Manaco | Bolívar | Venezuela | 13/06/1966 | 14/06/1966 |
|  | *Pteronotus parnellii* | 5 | Guasipati, Guasipati | Bolívar | Venezuela | 29/04/1966 |  |
|  | *Pteronotus parnellii* | 1 | La Paragua, Hato San José | Bolívar | Venezuela | 10/04/1967 |  |
|  | *Pteronotus parnellii* | 9 | El Dorado | Bolívar | Venezuela | 11/05/1966 | 16/05/1966 |
|  | *Pteronotus parnellii* | 1 | El Manteco, Los Patos | Bolívar | Venezuela | 11/04/1966 |  |
|  | *Pteronotus parnellii* | 2 | El Manteco, Río Supamo | Bolívar | Venezuela | 8/04/1966 | 10/04/1966 |
|  | *Sturnira lilium* (probably *S. giannae*) | 1 | Esmeralda, Río Cunucunuma, Belén | Amazonas | Venezuela | 3/01/1967 | 9/02/1967 |
|  | *Sturnira tildae* | 1 | Esmeralda, Río Cunucunuma, Belén | Amazonas | Venezuela | 3/01/1967 | 9/02/1967 |
|  | *Uroderma convexum* | 1 | Esmeralda, Río Cunucunuma, Belén | Amazonas | Venezuela | 3/01/1967 | 9/02/1967 |
|  | *Carollia perspicillata* | 2 | Esmeralda, Río Cunucunuma, Belén | Amazonas | Venezuela | 3/01/1967 | 9/02/1967 |
|  | *Pteronotus parnellii* | 35 | Esmeralda, Río Cunucunuma, Belén | Amazonas | Venezuela | 3/01/1967 | 9/02/1967 |
|  | *Pteronotus parnellii* | 11 | Esmeralda, Caño Culebra, Belén | Amazonas | Venezuela | 3/03/1967 |  |
|  | *Pteronotus parnellii* | 10 | Esmeralda, Boca Mavaca | Amazonas | Venezuela | 17/03/1967 | 24/03/1967 |
|  | *Pteronotus parnellii* | 40 | Esmeraldas, Río Mavaca | Amazonas | Venezuela | 3/04/1967 | 11/04/1967 |
|  | *Pteronotus parnellii* | 31 | Pto. Ayacucho, Río Manapiare, San Juan | Amazonas | Venezuela | 12/07/1967 | 27/07/1967 |
|  | *Pteronotus parnellii* | 2 | Río Orinoco, Tamatama | Amazonas | Venezuela | 27/04/1967 |  |
|  | *Pteronotus parnellii* | 2 | San Felipe, Minas de Aroa | Yaracuy | Venezuela | 14/07/1967 | 19/07/1967 |
| *Trichobius leiomotus* | *Mormoops megalophylla* | 4 | La Paragua, Hato San José | Bolívar | Venezuela |  |  |
|  | *Mormoops megalophylla* | 7 | San Felipe, Minas de Aroa | Yaracuy | Venezuela | 14/07/1967 | 19/07/1967 |
| *Trichobius longipilis* | *Pteropteryx macrotis* | 2 | Icabarú, Icabarú | Bolívar | Venezuela | 8/05/1968 |  |
|  | *Peropteryx macrotis* | 6 | Icabarú, Icabarú | Bolívar | Venezuela | 9/05/1968 |  |
|  | *Peropteryx macrotis* | 1 | El Dorado, Piedra Virgen | Bolívar | Venezuela | 29/05/1966 |  |
| *Trichobius keemani* | *Carollia perspicillata* | 1 | Santo Domingo, Selvas de San Camilo, Nulita | Apure | Venezuela | 21/01/1968 |  |
|  | *Micronycteris megalotis* | 1 | Altamira, Altamira | Barinas | Venezuela | 3/01/1968 |  |
|  | *Micronycteris megalotis* | 1 | Esmeralda, Río Mavaca | Amazonas | Venezuela | 13/04/1967 |  |
|  | *Micronycteris microtis* | 2 | Esmeralda, Boca Mavaca | Amazonas | Venezuela | 21/02/1966 |  |
| *Trichobius lionycteridis* | *Carollia perspicillata* | 1 | Santo Domingo, Selvas de San Camilo, Nulita | Apure | Venezuela | 2/01/1968 |  |
|  | *Sturnira lilium* (probably *S. giannae*) | 2 | El Dorado, El Manaco | Bolívar | Venezuela | 9/06/1966 | 25/06/1966 |
|  | *Platyrrhinus helleri* | 1 | El Dorado, El Manaco | Bolívar | Venezuela | 9/06/1966 | 25/06/1966 |
|  | *Lionycteris spurrelli* | 31 | El Dorado, El Manaco | Bolívar | Venezuela | 9/06/1966 | 25/06/1966 |
|  | *Carollia perspicillata* | 1 | Icabarú, El Pauji, Icabarú | Bolívar | Venezuela | 2/05/1968 | 8/05/1968 |
|  | *Lionycteris spurrelli* | 65 | Icabarú, El Pauji, Icabarú | Bolívar | Venezuela | 2/05/1968 | 8/05/1968 |
|  | *Lionycteris spurrelli* | 3 | Icabarú, Icabarú | Bolívar | Venezuela | 9/05/1968 |  |
|  | *Lionycteris spurrelli* | 4 | Icabarú, Icabarú | Bolívar | Venezuela | 8/05/1968 |  |
|  | *Lionycteris spurrelli* | 2 | Icabarú, El Pauji, Icabarú | Bolívar | Venezuela | 27/04/1968 |  |
|  | *Lionycteris spurrelli* | 14 | El Dorado | Bolívar | Venezuela | 10/05/1966 | 19/05/1966 |
|  | *Molossus aztecus* | 1 | Pto. Ayacucho, Río Manapiare, San Juan | Amazonas | Venezuela | 15/07/1967 | 20/07/1967 |
|  | *Lionycteris spurrelli* | 25 | Pto. Ayacucho, Río Manapiare, San Juan | Amazonas | Venezuela | 15/07/1967 | 20/07/1967 |
|  | *Lionycteris spurrelli* | 1 | Esmeralda, Río Cunucunuma, Belén | Amazonas | Venezuela | 1/01/1967 |  |
|  | *Lionycteris spurrelli* | 2 | Cabecera del Caño Culebra, Esmeralda | Amazonas | Venezuela | 6/02/1967 |  |
|  | *Lionycteris spurrelli* | 1 | Pto. Ayacucho, Raya, Pto. Ayacucho | Amazonas | Venezuela | 12/09/1967 |  |
|  | *Lionycteris spurrelli* | 4 | Pto. Ayacucho, nr. Morganito | Amazonas | Venezuela | 4/10/1967 |  |
|  | *Lionycteris spurrelli* | 14 | Río Orinoco, Tamatama | Amazonas | Venezuela | 1/05/1967 | 15-05-19167 |
| *Trichobius lonchophyllae* | *Lonchophylla robusta* | 42 | Altamira, Altamira | Barinas | Venezuela | 26/07/1967 |  |
|  | *Lonchophylla robusta* | 10 | Altamira, Altamira | Barinas | Venezuela | 25/07/1967 |  |
|  | *Sturnira lilium* (probably *S. giannae*) | 1 | Altamira, Altamira | Barinas | Venezuela | 25/07/1967 |  |
|  | *Lonchophylla robusta* | 12 | Altamira | Barinas | Venezuela | 20/07/1967 | 10/01/1968 |
|  | *Lonchophylla orienticolina* | 11 | Tame, Vda. Santa Inés | Arauca | Venezuela | 10/03/2019 |  |
|  | *Anoura latidens* | 1 | Tame, Vda. Santa Inés | Arauca | Venezuela | 10/03/2019 |  |
|  | *Myotis handleyi* | 3 | Arauca | Arauca | Venezuela | 2019 |  |
| *Trichobius jubatus* | *Molossus molossus* | 1 | Arauca, Vereda Las Plumas,Finca Los Iguanitos | Arauca | Venezuela | 22/11/2018 |  |
|  | *Molossus pretiossus* | 6 | Arauca, Vereda El Socorro, Finca Los Trompillos | Arauca | Venezuela | 29/07/2019 | 4/08/2019 |
| *Trichobius longipes* | *Phyllostomus hastatus* | 1 | Arauca, Campus La Nacional | Arauca | Venezuela | 27/07/2019 |  |
|  | *Phyllostomus hastatus* | 5 | Tame, Vda. Santa Inés , Finca La Porfia | Arauca | Venezuela | 4/03/2019 |  |
| *Trichobius uniformis* | *Glossophaga longirostris* | 1 | Pto. Páez, La Villa, Hato Cariben | Apure | Venezuela | 8/07/1965 |  |
|  | *Glossophaga longirostris* | 2 | Pto. Páez, Río Cinaruco, Hato Cariben | Apure | Venezuela | 14/07/1965 | 16/07/1965 |
|  | *Glossophaga soricina* | 2 | Altamira, Altamira | Barinas | Venezuela | 27/07/1967 | 1/01/1968 |
|  | *Glossophaga soricina* | 3 | Altamira | Barinas | Venezuela | 20/07/1967 |  |
|  | *Platyrrhinus helleri* | 1 | El Dorado, El Manaco | Bolívar | Venezuela | 8/07/1966 | 25/06/1966 |
|  | *Glossophaga soricina* | 22 | El Dorado, El Manaco | Bolívar | Venezuela | 8/07/1966 | 25/06/1966 |
|  | *Glossophaga longirostris* | 3 | La Paragua, Hato San José | Bolívar | Venezuela | 1/04/1967 | 10/04/1967 |
|  | *Glossophaga soricina* | 15 | La Paragua, Hato San José | Bolívar | Venezuela | 1/04/1967 | 10/04/1967 |
|  | *Glossophaga soricina* | 1 | Caicara, Hato La Florida | Bolívar | Venezuela | 4/05/1967 |  |
|  | *Glossophaga soricina* | 5 | El Dorado, El Manaco | Bolívar | Venezuela | 16/04/1966 |  |
|  | *Glossophaga soricina* | 22 | El Dorado, El Manaco | Bolívar | Venezuela | 8/07/1966 | 25/06/1966 |
|  | *Glossophaga soricina* | 1 | Icabarú, Santa Lucia de Surukun | Bolívar | Venezuela | 29/04/1968 |  |
|  | *Glossophaga soricina* | 2 | El Dorado | Bolívar | Venezuela | 11/05/1966 | 17/05/1966 |
|  | *Glossophaga soricina* | 1 | El Manteco, Río Supamo | Bolívar | Venezuela | 8/04/1966 |  |
|  | *Glossophaga soricina* | 2 | Calabozo, Est. Biol. De los Llanos | Guárico | Venezuela | 20/08/1968 |  |
|  | *Glossophaga soricina* | 1 | San Juan de los Morros, Hato. Las Palmitas | Guárico | Venezuela | 7/01/1968 |  |
|  | *Glossophaga soricina* | 1 | Caripe, San Agustín | Monagas | Venezuela | 12/07/1967 |  |
|  | *Artibeus lituratus* | 2 | Pto. Ayacucho, Río Manapiare, San Juan | Amazonas | Venezuela | 6/07/1967 | 27/07/1967 |
|  | *Glossophaga soricina* | 37 | Pto. Ayacucho, Río Manapiare, San Juan | Amazonas | Venezuela | 6/07/1967 | 27/07/1967 |
|  | *Glossophaga longirostris* | 1 | Pto. Ayacucho, Río Manapiare, San Juan | Amazonas | Venezuela | 6/07/1967 | 27/07/1967 |
|  | *Glossophaga longirostris* | 1 | Pto. Ayacucho, nr. Morganito | Amazonas | Venezuela | 4/10/1967 | 8/10/1967 |
|  | *Glossophaga soricina* | 4 | Pto. Ayacucho, nr. Morganito | Amazonas | Venezuela | 4/10/1967 | 8/10/1967 |
|  | *Glossophaga soricina* | 6 | Esmeralda, Río Cunucunuma, Belén | Amazonas | Venezuela | 1/01/1967 | 3/01/1967 |
|  | *Glossophaga soricina* | 1 | Pto. Ayacucho, Chaparrito | Amazonas | Venezuela | 2/10/1967 |  |
|  | *Glossophaga soricina* | 6 | Pto. Ayacucho, Las Queseras | Amazonas | Venezuela | 24/07/1967 | 27/09/1967 |
|  | *Glossophaga soricina* | 4 | Pto. Ayacucho, Guayabal | Amazonas | Venezuela | 7/10/1967 | 12/10/1967 |
|  | *Glossophaga soricina* | 5 | Río Orinoco, Tamatama | Amazonas | Venezuela | 28/04/1967 | 7/05/1967 |
|  | *Glossophaga soricina* | 1 | Valera, nr. Agua Viva | Trujillo | Venezuela | 14/09/1965 |  |
|  | *Glossophaga soricina* | 1 | Valera, nr. Agua Santa | Trujillo | Venezuela | 3/09/1965 |  |
|  | *Glossophaga soricina* | 12 | San Felipe, Minas de Aroa | Yaracuy | Venezuela | 6/07/1967 | 13/07/1967 |
|  | *Carollia perspicillata* | 1 | San Felipe, Minas de Aroa | Yaracuy | Venezuela | 6/07/1967 | 13/07/1967 |
| *Trichobius dugesii* | *Glossophaga soricina* | 3 | Santo Domingo, Selvas de San Camilo, Nulita | Apure | Venezuela | 30/01/1968 |  |
|  | *Glossophaga longirostris* | 78 | Pto. Páez, La Villa, Hato Cariben | Apure | Venezuela | 6/07/1965 | 28/07/1965 |
|  | *Glossophaga longirostris* | 2 | Pto. Páez, Río Cinaruco, Hato Cariben | Apure | Venezuela | 14/07/1965 | 16/07/1965 |
|  | *Glossophaga longirostris* | 3 | Pto. Páez, Cerro de Murcielagos, Pto. Páez | Apure | Venezuela | 24/01/1966 |  |
|  | *Glossophaga soricina* | 18 | Altamira, Altamira | Barinas | Venezuela | 26/07/1967 |  |
|  | *Glossophaga soricina* | 2 | Altamira, Altamira | Barinas | Venezuela | 20/07/1967 |  |
|  | *Trinycteris nicefori* | 2 | El Manteco, Los Patos | Bolívar | Venezuela | 5/04/1966 |  |
|  | *Glossophaga soricina* | 2 | El Dorado, El Manaco | Bolívar | Venezuela | 16/04/1966 |  |
|  | *Glossophaga soricina* | 15 | El Dorado, El Manaco | Bolívar | Venezuela | 13/06/1966 | 25/06/1966 |
|  | *Glossophaga soricina* | 12 | La Paragua, Hato San José | Bolívar | Venezuela | 1/04/1967 | 10/04/1967 |
|  | *Glossophaga longirostris* | 2 | San Juan de los Morros, Hato. Las Palmitas | Guárico | Venezuela | 7/01/1968 |  |
|  | *Glossophaga soricina* | 1 | San Juan de los Morros, Hato. Las Palmitas | Guárico | Venezuela | 7/01/1968 |  |
|  | *Glossophaga soricina* | 2 | Calabozo, nr. Río Orituco, Est. Biol. De los Llanos | Guárico | Venezuela | 21/08/1968 |  |
|  | *Glossophaga soricina* | 1 | Calabozo, Est. Biol. De los Llanos | Guárico | Venezuela | 20/08/1968 |  |
|  | *Carollia brevicauda* | 1 | Caripe, nr. San Agustín | Monagas | Venezuela | 11/07/1967 |  |
|  | *Glossophaga soricina* | 5 | Maturín, Hato Mata de Bejuco | Monagas | Venezuela | 3/06/1968 |  |
|  | *Glossophaga longirostris* | 3 | Pto. Ayacucho, nr. Morganito | Amazonas | Venezuela | 4/10/1967 |  |
|  | *Glossophaga soricina* | 2 | Pto. Ayacucho, nr. Morganito | Amazonas | Venezuela | 4/10/1967 |  |
|  | *Platyrrhinus helleri* | 6 | Pto. Ayacucho, Río Manapiare, San Juan | Amazonas | Venezuela | 10/07/1967 | 27/09/1967 |
|  | *Glossophaga soricina* | 21 | Pto. Ayacucho, Río Manapiare, San Juan | Amazonas | Venezuela | 10/07/1967 | 27/09/1967 |
|  | *Glossophaga soricina* | 2 | Río Orinoco, Tamatama | Amazonas | Venezuela | 27/04/1967 | 7/05/1967 |
|  | *Glossophaga soricina* | 1 | Esmeralda, Río Cunucunuma, Belén | Amazonas | Venezuela | 3/01/1967 |  |
|  | *Glossophaga soricina* | 2 | Pto. Ayacucho, Las Queseras | Amazonas | Venezuela | 24/07/1967 | 27/09/1967 |
|  | *Glossophaga soricina* | 1 | Pto. Ayacucho, Guayabal | Amazonas | Venezuela | 7/10/1967 |  |
|  | *Glossophaga soricina* | 1 | Pto. Ayacucho, Raya, Pto. Ayacucho | Amazonas | Venezuela | 7/09/1967 |  |
|  | *Glossophaga soricina* | 1 | Valera, Agua Santa | Trujillo | Venezuela | 3/09/1965 |  |
|  | *Glossophaga longirostris* | 2 | Valera, Agua Santa | Trujillo | Venezuela | 3/09/1965 |  |
|  | *Glossophaga longirostris* | 1 | Valera, Agua Santa | Trujillo | Venezuela | 19/08/1965 | 23/08/1965 |
|  | *Glossophaga soricina* | 2 | Valera, Agua Santa | Trujillo | Venezuela | 19/08/1965 | 23/08/1965 |
|  | *Glossophaga longirostris* | 4 | Valera, Quebrada Seca | Trujillo | Venezuela | 21/10/1965 |  |
|  | *Glossophaga soricina* | 7 | San Felipe, Minas de Aroa | Yaracuy | Venezuela | 12/07/1967 | 21/07/1967 |
| *Trichobius propinquus* | *Anoura geoffroyi* | 1 | El Dorado | Bolívar | Venezuela | 19/05/1966 |  |
| *Trichobius joblingi* | *Carollia perspicillata* | 2113 | Barinas (3 localities, 611-1,070 m); Bolivar (15 localities, 50-1,042 m): Carabobo (3 localities, 25-1,537 m); Dto. Federal (2 localities, 380-1,524 111); Falcon (9 localities, 2-1,260 in); Guarico (4 localities, 100-630 m); Miranda (7 localities, 1-1,160 m); Monagas (3 localities, 854- 1,320 m); Aniazonas (15 localities, 114-195 m); Trujillo (7 localities, 23-164 ni); Yaracuv (2 localities, 25- 400 in); Zulia (13 localities, 24-270 m). | Barinas, Bolívar, Carabobo, Dto. Federal, Falcon, Guarico, Miranda, Monagas, Amazonas, Trujillo, Yaracuy y Zulia | Venezuela | 1960 | 1967 |
|  | *Phyllostomus elongatus* | 242 | NA | NA | Venezuela | 1960 | 1967 |
|  | *Carollia brevicauda* | 36 | NA | NA | Venezuela | 1960 | 1967 |
|  | *Carollia perspicillata* | 7 | El Dorado | Bolívar | Venezuela | 30/08/1962 |  |
|  | *Carollia perspicillata* | 15 | Guacharo Cave | Monagas | Venezuela | 16/08/1962 |  |
|  | *Carollia perspicillata* | 78 | Reserva Natural La Palmita | Casanare | Colombia | 2017 |  |
|  | *Carollia brevicauda* | 28 | Reserva Natural La Palmita | Casanare | Colombia | 2017 |  |
|  | *Desmodus rotundus* | 4 | Reserva Natural La Palmita | Casanare | Colombia | 2017 |  |
|  | *Platyrrhinus fusciventris* | 1 | Arauca | Arauca | Colombia | 2018 | 2019 |
|  | *Carollia perspicillata* | 3 | Tame | Arauca | Colombia | 2018 | 2019 |
|  | *Carollia brevicauda* | 5 | Tame | Arauca | Colombia | 2018 | 2019 |
|  | *Phyllostomus hastatus* | 1 | Tame | Arauca | Colombia | 2018 | 2019 |
| *Trichobius permilis* | *Carollia brevicauda* | 1 | Santo Domingo, Selvas de San Camilo, Nulita | Apure | Venezuela | 22/01/1968 |  |
|  | *Carollia perspicillata* | 2 | Montalbán, La Copa | Carabobo | Venezuela | 26/11/1967 | 30/11/1967 |
|  | *Carollia brevicauda* | 46 | Montalbán, La Copa | Carabobo | Venezuela | 26/11/1967 | 30/11/1967 |
|  | *Carollia brevicauda* | 1 | Montabán, Hda. La Canada | Carabobo | Venezuela | 22/11/1967 |  |
|  | *Carollia brevicauda* | 5 | Montabán, La Leonera | Carabobo | Venezuela | 22/11/1967 | 23/11/1967 |
|  | *Carollia brevicauda* | 2 | Montalbán, Cumbre Canoabo | Carabobo | Venezuela | 1/11/1967 |  |
|  | *Carollia perspicillata* | 1 | Altamira | Barinas | Venezuela | 14/07/1967 |  |
|  | *Carollia brevicauda* | 22 | Altamira | Barinas | Venezuela | 10/01/1968 |  |
|  | *Carollia brevicauda* | 3 | Altamira | Barinas | Venezuela | 14/07/1967 |  |
|  | *Carollia brevicauda* | 26 | Altamira | Barinas | Venezuela | 27/07/1968 |  |
|  | *Carollia brevicauda* | 9 | Altamira | Barinas | Venezuela | 13/07/1967 |  |
|  | *Carollia brevicauda* | 1 | Altamira | Barinas | Venezuela | 25/07/1967 |  |
|  | *Carollia perspicillata* | 4 | La Paragua, Hato San José | Bolívar | Venezuela | 6/03/1967 |  |
|  | *Carollia perspicillata* | 1 | Icabarú, Santa Lucia de Surukun | Bolívar | Venezuela | 30/04/1968 |  |
|  | *Phyllostomus elongatus* | 3 | El Manteco, Los Patos | Bolívar | Venezuela | 5/04/1966 |  |
|  | *Carollia brevicauda* | 6 | Caripe, nr. San Agustín | Monagas | Venezuela | 11/07/1967 |  |
|  | *Carollia brevicauda* | 2 | Caripe, San Agustín | Monagas | Venezuela | 26/06/1967 |  |
| *Trichobius macrophylli* | *Macrophyllum macrophyllum* | 10 | Pto. Páez, La Villa, Hato Cariben | Apure | Venezuela | 6/07/1965 |  |
|  | *Macrophyllum macrophyllum* | 2 | El Dorado, El Manaco | Bolívar | Venezuela | 10/06/1966 |  |
|  | *Macrophyllum macrophyllum* | 1 | El Manteco, Río Supamo | Bolívar | Venezuela | 11/04/1966 |  |
|  | *Macrophyllum macrophyllum* | 20 | San Juan de los Morros, Hato. Las Palmitas | Guárico | Venezuela | 7/01/1968 |  |
|  | *Macrophyllum macrophyllum* | 5 | Esmeralda, Río Cunucunuma, Belén | Amazonas | Venezuela | 10/02/1967 |  |
|  | *Macrophyllum macrophyllum* | 21 | Esmeralda, Río Mavaca | Amazonas | Venezuela | 10/04/1967 |  |
| *Trichobius handleyi* | *Micronycteris minuta* | 3 | Pto. Páez, Río Cinaruco, Hato Cariben | Apure | Venezuela | 14/07/1965 |  |
|  | *Micronycteris minuta* | 2 | Pto. Páez, Río Cinaruco, Hato Cariben | Apure | Venezuela | 13/01/1966 |  |
|  | *Micronycteris minuta* | 3 | El Manteco, Los Patos | Bolívar | Venezuela | 11/04/1966 |  |
|  | *Phyllostomus elongatus* | 1 | El Manteco, Río Supamo | Bolívar | Venezuela | 30/03/1966 |  |
|  | *Micronycteris minuta* | 1 | Calabozo, nr. Río Orituco, Est. Biol. De los Llanos | Guárico | Venezuela | 22/08/1968 |  |
|  | *Micronycteris minuta* | 3 | Maturín, Hato Mata de Bejuco | Monagas | Venezuela | 3/06/1968 |  |
|  | *Micronycteris minuta* | 1 | Pto. Ayacucho, Río Manapiare, San Juan | Amazonas | Venezuela | 18/07/1967 |  |
|  | *Micronycteris minuta* | 4 | Valera, nr. Agua Viva | Trujillo | Venezuela | 3/09/1965 | 7/10/1965 |
| *Trichobius urodermae* | *Uroderma bilobatum* | 1 | Esmeralda, Caño Culebra, Belén | Amazonas | Venezuela | 2/02/1967 |  |
|  | *Uroderma bilobatum* | 3 | Esmeralda, Río Cunucunuma, Belén | Amazonas | Venezuela | 6/01/1967 | 3/02/1967 |
|  | *Uroderma bilobatum* | 5 | Pto. Ayacucho, nr. Morganito | Amazonas | Venezuela | 8/10/1967 |  |
|  | *Uroderma bilobatum* | 3 | Valera, La Ceiba | Trujillo | Venezuela | 27/10/1965 |  |
|  | *Uroderma bilobatum* | 1 | Valera, nr. Agua Santa | Trujillo | Venezuela | 7/09/1965 |  |
| *Trichobius tiptoni* | *Anoura caudifer* | 26 | Altamira, Altamira | Barinas | Venezuela | 26/07/1967 | 4/01/1968 |
|  | *Sturnira ludovici* | 1 | Altamira, Altamira | Barinas | Venezuela | 26/07/1967 | 4/01/1968 |
|  | *Desmodus rotundus* | 1 | Altamira, Altamira | Barinas | Venezuela | 26/07/1967 | 4/01/1968 |
|  | *Carollia perspicillata* | 1 | Altamira | Barinas | Venezuela | 13/07/1967 | 20/07/1967 |
|  | *Anoura caudifer* | 12 | Altamira | Barinas | Venezuela | 13/07/1967 | 20/07/1967 |
|  | *Anoura caudifer* | 2 | Altamira, Altamira | Barinas | Venezuela | 26/07/1967 |  |
|  | *Anoura caudifer* | 14 | El Dorado | Bolívar | Venezuela | 10/05/1966 | 23/05/1966 |
|  | *Anoura caudifer* | 4 | Montalbán, La Copa | Carabobo | Venezuela | 30/11/1967 | 1/12/1967 |
|  | *Anoura caudifer* | 4 | San Felipe, Minas de Aroa | Yaracuy | Venezuela | 6/07/1967 | 21/07/1967 |
|  | *Platyrrhinus helleri* | 1 | San Felipe, Minas de Aroa | Yaracuy | Venezuela | 6/07/1967 | 21/07/1967 |
| *Trichobius angulatus* | *Platyrrhynus auraritus* | 19 | El Dorado | Bolívar | Venezuela | 20/04/1966 |  |
|  | *Platyrrhynus auraritus* | 5 | Caño Culebra, Esmeralda | Amazonas | Venezuela | 17/01/1967 | 19/01/1967 |
| *Trichobius assimilis* | *Artibeus planirostris* | 5 | Icabarú, El Pauji, Icabarú | Bolívar | Venezuela | 7/05/1968 | 9/05/1968 |
|  | *Artibeus planirostris* | 1 | Icabarú, Icabarú | Bolívar | Venezuela | 28/04/1968 |  |
|  | *Artibeus planirostris* | 10 | El Dorado | Bolívar | Venezuela | 19/05/1966 |  |
|  | *Platyrrhynus auraritus* | 1 | El Dorado | Bolívar | Venezuela | 19/05/1966 |  |
|  | *Artibeus planirostris* | 4 | Esmeralda, Caño Culebra, Belén | Amazonas | Venezuela | 12/01/1967 | 2/02/1967 |
|  | *Artibeus planirostris* | 4 | Esmeralda, Río Cunucunuma, Belén | Amazonas | Venezuela | 10/01/1967 | 3/02/1967 |
|  | *Artibeus planirostris* | 13 | Cabecera del Caño Culebra, Esmeralda | Amazonas | Venezuela | 2/02/1967 | 7/02/1967 |
| *T. dugesioides (probably Trichobius anducei)* | *Carollia perspicillata* | 3 | Santo Domingo, Selvas de San Camilo, Nulita | Apure | Venezuela | 22/01/1968 | 31-ene |
|  | *Carollia perspicillata* | 5 | Altamira, Altamira | Barinas | Venezuela | 27/07/1967 | 4/01/1968 |
|  | *Carollia perspicillata* | 2 | Altamira, Altamira | Barinas | Venezuela | 25/07/1967 |  |
|  | *Carollia perspicillata* | 3 | Altamira | Barinas | Venezuela | 13/07/1967 | 9/01/1968 |
|  | *Carollia perspicillata* | 1 | Icabarú, El Pauji, Icabarú | Bolívar | Venezuela | 2/05/1968 |  |
|  | *Carollia perspicillata* | 3 | Icabarú, El Pauji, Icabarú | Bolívar | Venezuela | 28/04/2968 | 29/04/1968 |
|  | *Carollia perspicillata* | 4 | Icabarú, Santa Lucia de Surukun | Bolívar | Venezuela | 30/04/1968 | 2/05/1968 |
|  | *Carollia perspicillata* | 1 | Pto. Ayacucho, Raya, Pto. Ayacucho | Amazonas | Venezuela | 7/09/1967 |  |
|  | *Carollia perspicillata* | 1 | Esmeralda, Caño Culebra, Belén | Amazonas | Venezuela | 12/01/1967 |  |
|  | *Carollia perspicillata* | 5 | Esmeralda, Río Cunucunuma, Belén | Amazonas | Venezuela | 10/01/1967 | 9/02/1967 |
|  | *Carollia perspicillata* | 1 | Pto. Ayacucho, Las Queseras | Amazonas | Venezuela | 27/09/1967 |  |
|  | *Carollia perspicillata* | 11 | Esmeralda, Río Mavaca | Amazonas | Venezuela | 3/04/1967 | 11/04/1967 |
|  | *Carollia perspicillata* | 2 | San Felipe, Minas de Aroa | Yaracuy | Venezuela | 7/07/1967 | 13/07/1967 |
| *Trichobius dugesioides* | *Trachops cirrhosus* | 2 | Santo Domingo, Selvas de San Trmilo, Nulita | Apure | Venezuela | 22/01/1968 | 31/01/1968 |
|  | *Macrophyllum macrophyllum* | 1 | Pto. Páez, La Villa, Hato Cariben | Apure | Venezuela | 23/07/1965 | 28/07/1965 |
|  | *Trachops cirrhosus* | 32 | Pto. Páez, La Villa, Hato Cariben | Apure | Venezuela | 23/07/1965 | 28/07/1965 |
|  | *Trachops cirrhosus* | 8 | Pto. Páez, Río Cinaruco, Hato Cariben | Apure | Venezuela | 14/07/1965 | 27/07/1965 |
|  | *Desmodus rotundus* | 3 | Pto. Páez, Río Cinaruco, Hato Cariben | Apure | Venezuela | 14/07/1965 | 27/07/1965 |
|  | *Carollia brevicauda* | 1 | Altamira, Altamira | Barinas | Venezuela | 13/07/1967 |  |
|  | *Trachops cirrhosus* | 5 | Icabarú, Santa Lucia de Surukun | Bolívar | Venezuela | 30/04/1968 | 2/05/1968 |
|  | *Trachops cirrhosus* | 10 | El Dorado, El Manaco | Bolívar | Venezuela | 14/06/1966 | 23/06/1966 |
|  | *Trachops cirrhosus* | 6 | La Paragua, Hato San José | Bolívar | Venezuela | 10/04/1967 |  |
|  | *Trachops cirrhosus* | 1 | El Manteco, Río Supamo | Bolívar | Venezuela | 30/03/1966 | 10-abr |
|  | *Trachops cirrhosus* | 74 | Calabozo, nr. Río Orituco, Est. Biol. De los Llanos | Guárico | Venezuela | 22/08/1964 | 22/08/1968 |
|  | *Trachops cirrhosus* | 3 | Esmeralda, Boca Mavaca | Amazonas | Venezuela | 20/02/1966 |  |
|  | *Trachops cirrhosus* | 6 | Esmeralda, Brazo Casiquiare, Capibara | Amazonas | Venezuela | 30/05/1967 | 2/06/1967 |
|  | *Trachops cirrhosus* | 11 | Pto. Ayacucho, El Gavilan | Amazonas | Venezuela | 11/10/1967 |  |
|  | *Trachops cirrhosus* | 4 | Pto. Ayacucho, Raya, Pto. Ayacucho | Amazonas | Venezuela | 7/09/1967 |  |
|  | *Chrotopterus auritus* | 1 | Esmeralda, Río Cunucunuma, Belén | Amazonas | Venezuela | 10/01/1967 | 9/02/1967 |
|  | *Trachops cirrhosus* | 9 | Pto. Ayacucho, El Raudal | Amazonas | Venezuela | 20/09/1967 | 5/10/1967 |
|  | *Phyllostomus hastatus* | 1 | Río Orinoco, Tamatama | Amazonas | Venezuela | 28/04/1967 | 8/05/1967 |
|  | *Lophostoma silvicola* | 1 | Esmeralda, Río Mavaca | Amazonas | Venezuela | 3/04/1967 | 11/04/1967 |
|  | *Phyllostomus discolor* | 1 | Esmeralda, Río Mavaca | Amazonas | Venezuela | 3/04/1967 | 11/04/1967 |
|  | *Sphaeronycteris toxophyllum* | 1 | Esmeralda, Río Mavaca | Amazonas | Venezuela | 3/04/1967 | 11/04/1967 |
|  | *Trachops cirrhosus* | 182 | Esmeralda, Río Mavaca | Amazonas | Venezuela | 3/04/1967 | 11/04/1967 |
|  | *Chrotopterus auritus* | 2 | Esmeralda, Río Mavaca | Amazonas | Venezuela | 3/04/1967 | 11/04/1967 |
|  | *Trachops cirrhosus* | 95 | Pto. Ayacucho, Río Manapiare, San Juan | Amazonas | Venezuela | 5/07/1967 | 27/07/1967 |
|  | *Chrotopterus auritus* | 19 | Pto. Ayacucho, Río Manapiare, San Juan | Amazonas | Venezuela | 5/07/1967 | 27/07/1967 |
|  | *Chrotopterus auritus* | 1 | Valera, nr. Agua Viva | Trujillo | Venezuela | 6/06/1965 |  |
|  | *Trachops cirrhosus* | 2 | Valera, Rí Motatan | Trujillo | Venezuela | 8/10/1965 |  |
|  | *Trachops cirrhosus* | 2 | Valera, nr. Agua Santa | Trujillo | Venezuela | 22/10/1965 |  |
|  | *Trachops cirrhosus* | 4 | Urama, El Central | Yaracuy | Venezuela | 15/03/1966 |  |
| *Trichobius dugesioides phyllostomus* | *Phyllostomus elongatus* | 1 | Río Orinoco, Tamatama | Amazonas | Venezuela | 28/04/1967 | 8/05/1967 |
|  | *Phyllostomus elongatus* | 5 | El Manteco, Río Supamo | Bolívar | Venezuela | 30/03/1966 |  |
|  | *Phyllostomus elongatus* | 1 | El Dorado, Piedra Virgen | Bolívar | Venezuela | 29/05/1966 |  |
|  | *Phyllostomus elongatus* | 2 | El Manteco, Los Patos | Bolívar | Venezuela | 5/04/1966 |  |
|  | *Phyllostomus elongatus* | 1 | Esmeralda, Río Mavaca | Amazonas | Venezuela | 3/04/1967 | 11/04/1967 |
|  | *Phyllostomus elongatus* | 1 | Pto. Ayacucho, Río Manapiare, San Juan | Amazonas | Venezuela | 5/07/1967 | 27/07/1967 |
| *Trichobius tuttlei* | *Micronycteris brachyotis* | 3 | Río Orinoco, Tamatama | Amazonas | Venezuela | 4/05/1967 |  |
| *Trichobius ethophallus* | *Lonchorhina orinocensis* | 1 | Pto. Páez, La Villa, Hato Cariben | Apure | Venezuela | 24/07/1965 |  |
|  | *Lonchorhina orinocensis* | 1 | Pto. Páez, La Villa, Hato Cariben | Apure | Venezuela | 23/07/1965 |  |
|  | *Lonchorhina orinocensis* | 403 | Pto. Páez, La Villa, Hato Cariben | Apure | Venezuela | 6/07/1965 | 28/07/1965 |
|  | *Lonchorhina orinocensis* | 8 | Pto. Páez, Cerro de Murcielagos, Pto. Páez | Apure | Venezuela | 19/01/1966 | 24/01/1966 |
|  | *Lonchorhina orinocensis* | 3 | Pto. Ayacucho, Las Queseras | Amazonas | Venezuela | 21/09/1967 |  |
|  | *Lonchorhina orinocensis* | 2 | Pto. Ayacucho, Coromoto | Amazonas | Venezuela | 8/10/1967 |  |
|  | *Lonchorhina orinocensis* | 1 | Pto. Ayacucho, Guayabal | Amazonas | Venezuela | 12/10/1967 |  |
| *Trichobius flagellatus* | *Lonchorhina aurita* | 2 | Valera, nr. Agua Viva | Trujillo | Venezuela | 3/09/1965 |  |
|  | *Lonchorhina aurita* | 1 | Altamira, Altamira | Barinas | Venezuela | 25/07/1967 |  |
|  | *Lonchorhina aurita* | 14 | La Paragua, Hato San José | Bolívar | Venezuela | 8/04/1967 |  |
|  | *Lonchorhina orinocensis* | 2 | Pto. Ayacucho, Paria | Amazonas | Venezuela | 13/09/1967 |  |
|  | *Lonchorhina aurita* | 1 | Esmeralda, Caño Culebra, Belén | Amazonas | Venezuela | 6/02/1967 |  |
|  | *Lonchorhina aurita* | 2 | Esmeralda, Río Mavaca | Amazonas | Venezuela | 2/03/1967 |  |
| *Trichobius diphyllae* | *Diphylla ecaudata* | 7 | Rancho Grande, El Limón | Aragua | Venezuela |  |  |
| *Trichobius diaemi* | *Diaemus youngii* | 2 | Pto. Ayacucho, Río Manapiare, San Juan | Amazonas | Venezuela | 17/07/1967 | 18/07/1967 |
|  | *Diaemus youngii* | 9 | Pto. Ayacucho, Chaparito | Amazonas | Venezuela | 2/10/1967 |  |
|  | *Diaemus youngii* | 47 | Pto. Ayacucho, Guayabal | Amazonas | Venezuela | 7/10/1967 |  |
|  | *Diaemus youngii* | 18 | Pto. Ayacucho, Río Manapiare, San Juan | Amazonas | Venezuela | 17/07/1967 | 18/07/1967 |
|  | *Diaemus youngii* | 2 | Guainia, nr. Amanaven | Guainía | Colombia | 17/09/1967 |  |
| *Trichobius parasiticus* | *Desmodus rotundus* | 4146 | Apure (6 localities); Barinas (3 localities); Bolivar (2 localities); Carabobo (4 localities); Dto. Federal (2 localities); Falcon ( 5 localities) ; Guarico (3 localities); Lara (1 localities); Miranda (7 localities); Monagas (3 locahties); Amazonas ( 12 localities); Trujillo (7 localities); Yaracuy (1 locality); Zulia (4 localities). | Apure, Barinas, Bolívar, Carabobo, Dto. Federal, Falcon, Guarico, Lara, Miranda, Monagas, Amazonas, Trujillo, Yaracuy y Zulia | Venezuela | 1960 | 1967 |
|  | *Platyrrhinus umbratus* | 17 | NA | NA | Venezuela |  |  |
|  | *Chiroderma villosum* | 16 | NA | NA | Venezuela |  |  |
|  | *Carollia perspicillata* | 26 | NA | NA | Venezuela |  |  |
|  | *Desmodus rotundus* | 2 | Arauca y Meta (El Parque La Macarena, Cabana Duda, 20 ft e; San Juan de Arama, Los Micos) | Arauca y Meta | Colombia |  |  |
| *Trichobius costalimai* | *Phyllostomus discolor* | 2111 | Barinas (1 locality); Bolivar (2 localities); Carabobo (2 localities); Dto. Federal (2 localities); Falcon (4 localities); Guarico (2 localities); Miranda (3 localittes); Monagas (2 localities); Amazonas (4 localities); Trujillo (2 localities); Zulia (6 localities). | Barinas, Bolívar, Carabobo, Dto. Federal, Falcon, Guarico, Miranda, Monagas, Amazonas, Trujillo y Zulia | Venezuela | 1960 | 1967 |
|  | *Eptesicus orinocensis* | 1 | Cravo Norte, Vereda Las plmas, El deleite | Arauca | Colombia | 26/11/2018 |  |
|  | *Phyllostomus discolor* | 3 | Cravo Norte, Vereda Las plmas, El deleite | Arauca | Colombia | 26/11/2018 |  |
|  | *Phyllostomus discolor* | 25 | Tame, Vda. Santa Inés | Arauca | Colombia | 12/03/2019 |  |
|  | *Phyllostomus elongatus* | 8 | Tame, Vda. Santa Inés | Arauca | Colombia | 12/03/2019 |  |
| *Trichobius longipes* | *Molossus rufus* | 1 | Pto. Páez | Apure | Venezuela | 17/01/1966 |  |
|  | *Phyllostomus hastatus* | 83 | Santo Domingo, Selvas de San Camilo, Nulita | Apure | Venezuela | 17/01/1968 | 1/02/1968 |
|  | *Phyllostomus hastatus* | 1 | Montalbán, Hda. La Canada | Carabobo | Venezuela | 22/11/1967 |  |
|  | *Phyllostomus hastatus* | 4 | Altamira | Barinas | Venezuela | 21/07/1967 |  |
|  | *Phyllostomus elongatus* | 1 | El Manteco, Los Patos | Bolívar | Venezuela | 5/04/1966 |  |
|  | *Phyllostomus hastatus* | 18 | El Dorado, El Manaco | Bolívar | Venezuela | 8/06/1966 | 20/06/1966 |
|  | *Phyllostomus hastatus* | 1 | Icabarú | Bolívar | Venezuela | 9/05/1968 |  |
|  | *Phyllostomus hastatus* | 2 | El Manteco, Los Patos | Bolívar | Venezuela | 11/04/1966 |  |
|  | *Phyllostomus hastatus* | 4 | Calabozo, nr. Río Orituco, Est. Biol. De los Llanos | Guárico | Venezuela | 21/08/1968 | 23/08/1968 |
|  | *Desmodus rotundus* | 1 | Calabozo, nr. Río Orituco, Est. Biol. De los Llanos | Guárico | Venezuela | 21/08/1968 | 23/08/1968 |
|  | *Phyllostomus elongatus* | 34 | Calabozo, nr. Río Orituco, Est. Biol. De los Llanos | Guárico | Venezuela | 21/08/1968 | 23/08/1968 |
|  | *Phyllostomus elongatus* | 10 | Calabozo, Est. Biol. De los Llanos | Guárico | Venezuela | 20/08/1968 |  |
|  | *Phyllostomus elongatus* | 3 | Maturín, Hato Mata de Bejuco | Monagas | Venezuela | 3/06/1968 | 4/06/1968 |
|  | *Phyllostomus hastatus* | 21 | Maturín, Hato Mata de Bejuco | Monagas | Venezuela | 3/06/1968 | 4/06/1968 |
|  | *Uroderma bilobatum* | 1 | Pto. Ayacucho, Río Manapiare, San Juan | Amazonas | Venezuela | 5/07/1967 | 28/07/1967 |
|  | *Artibeus planirostris* | 3 | Pto. Ayacucho, Río Manapiare, San Juan | Amazonas | Venezuela | 5/07/1967 | 28/07/1967 |
|  | *Rhynchonycteris naso* | 1 | Pto. Ayacucho, Río Manapiare, San Juan | Amazonas | Venezuela | 5/07/1967 | 28/07/1967 |
|  | *Phyllostomus elongatus* | 30 | Pto. Ayacucho, Río Manapiare, San Juan | Amazonas | Venezuela | 5/07/1967 | 28/07/1967 |
|  | *Phyllostomus hastatus* | 168 | Pto. Ayacucho, Río Manapiare, San Juan | Amazonas | Venezuela | 5/07/1967 | 28/07/1967 |
|  | *Phyllostomus elongatus* | 6 | Pto. Ayacucho, Paria | Amazonas | Venezuela | 13/09/1967 | 4/10/1967 |
|  | *Phyllostomus elongatus* | 5 | Pto. Ayacucho, Paria | Amazonas | Venezuela | 13/09/1967 | 4/10/1967 |
|  | *Phyllostomus elongatus* | 3 | Río Orinoco, Tamatama | Amazonas | Venezuela | 27/04/1967 | 7/05/1967 |
|  | *Phyllostomus hastatus* | 2 | Río Orinoco, Tamatama | Amazonas | Venezuela | 27/04/1967 | 7/05/1967 |
|  | *Phyllostomus hastatus* | 11 | Esmeralda, Brazo Casiquiare, Capibara | Amazonas | Venezuela | 29/05/1967 | 1/06/1967 |
|  | *Phyllostomus hastatus* | 3 | Pto. Ayacucho, Coromoto | Amazonas | Venezuela | 11/09/1967 |  |
|  | *Phyllostomus hastatus* | 4 | Pto. Ayacucho, Raya, Pto. Ayacucho | Amazonas | Venezuela | 6/09/1967 | 7/09/1967 |
|  | *Phyllostomus hastatus* | 2 | Pto. Ayacucho, El Raudal | Amazonas | Venezuela | 20/09/1967 |  |
|  | *Phyllostomus elongatus* | 4 | Esmeralda, Boca Mavaca | Amazonas | Venezuela | 20/02/1966 | 24/03/1967 |
|  | *Phyllostomus elongatus* | 14 | Esmeralda, Río Mavaca | Amazonas | Venezuela | 5/04/1967 | 14/04/1967 |
|  | *Phyllostomus hastatus* | 1 | Valera, nr. Agua Viva | Trujillo | Venezuela | 5/09/1965 |  |
|  | *Phyllostomus hastatus* | 8 | Valera, nr. Agua Viva | Trujillo | Venezuela | 16/09/1965 |  |
|  | *Phyllostomus hastatus* | 1 | Valera, Río Motatan | Trujillo | Venezuela | 2/09/1965 |  |
|  | *Phyllostomus hastatus* | 2 | Valera, nr. Agua Santa | Trujillo | Venezuela | 18/10/1965 | 22/10/1965 |
|  | *Uroderma bilobatum* | 1 | Urama, El Central | Yaracuy | Venezuela | 14/03/1966 |  |
|  | *Phyllostomus hastatus* | 29 | Urama, El Central | Yaracuy | Venezuela | 14/03/1966 |  |
|  | *Phyllostomus hastatus* | 5 | Urama, El Central | Yaracuy | Venezuela | 20/03/1966 |  |
| *Trichobius silvicolae* | *Lophostoma silvicola* | 2 | Esmeralda, Brazo Casiquiare, Capibara | Amazonas | Venezuela | 30/05/1967 |  |
|  | *Phyllostomus hastatus* | 1 | El Dorado, El Manaco | Bolívar | Venezuela | 15/06/1966 |  |
|  | *Lophostoma silvicola* | 9 | Esmeralda, Río Cunucunuma, Belén | Amazonas | Venezuela | 3/01/1967 |  |
|  | *Lophostoma silvicola* | 10 | Esmeralda, Brazo Casiquiare, Capibara | Amazonas | Venezuela | 12/06/1967 |  |
|  | *Lophostoma silvicola* | 2 | Esmeralda, Río Mavaca | Amazonas | Venezuela | 5/04/1967 | 12/04/1967 |
| *Trichobius affinis* | *Lophostoma brasiliense* | 2 | Pto. Ayacucho, Río Manapiare, San Juan | Amazonas | Venezuela | 27/07/1967 |  |
|  | *Lophostoma brasiliense* | 2 | Santo Domingo, Selvas de San Camilo, Nulita | Apure | Venezuela | 23/01/1968 |  |
|  | *Lophostoma brasiliense* | 1 | Pto. Ayacucho, Río Manapiare, San Juan | Amazonas | Venezuela | 27/07/1967 |  |
| *Trichobius strictisternus* | *Lophostoma carrikeri* | 1 | Pto. Ayacucho, Río Manapiare, San Juan | Amazonas | Venezuela | 24/07/1967 |  |
| *Trichobius imitator* | *Anoura sp.* | 1 | Caicara, Hato La Florida | Bolívar | Venezuela | 5/04/1967 |  |
| *Trichobius jubatus* | *Molossus rufus* | 2 | Pto. Páez, Río Cinaruco, Hato Cariben | Apure | Venezuela | 17/07/1965 |  |
|  | *Molossus rufus* | 1 | Pto. Páez, Hato Cariben | Apure | Venezuela | 17/07/1965 |  |
|  | *Molossus rufus* | 5 | Pto. Páez, Río Cinaruco, Hato Cariben | Apure | Venezuela | 13/07/1965 | 17/07/1965 |
|  | *Molossus rufus* | 14 | Caripe, San Agustín | Monagas | Venezuela | 28/06/1967 | 8/07/1967 |
|  | *Molossus rufus* | 2 | Esmeralda, Río Cunucunuma, Belén | Amazonas | Venezuela | 7/01/1967 |  |
|  | *Molossus rufus* | 2 | Pto. Ayacucho, Río Manapiare, San Juan | Amazonas | Venezuela | 18/07/1967 | 24/07/1967 |
|  | *Molossus rufus* | 1 | Pto. Ayacucho, Río Manapiare, San Juan | Amazonas | Venezuela | 25/07/1967 |  |
|  | *Molossus rufus* | 1 | Arauca, Vereda El Socorro, Finca Marsella | Arauca | Venezuela | 3/11/2021 |  |
| *Trichobius vampyrops* | *Platyrrhinus vittatus* | 1 | Altamira, Altamira | Barinas | Venezuela | 5/01/1969 |  |
| *Trichobius petersoni* | *Sturnira erythromos* | 2 | Caripe, nr. San Agustín | Monagas | Venezuela | 11/07/1967 |  |
|  | *Sturnira bogotensis* | 1 | Tabay, La Mucuy | Mérida | Venezuela | 8/03/1966 |  |
|  | *Sturnira erythromos* | 12 | La Azulita, La Carbonera | Mérida | Venezuela | 21/04/1966 | 23/04/1966 |
|  | *Sturnira erythromos* | 3 | La Azulita, La Carbonera | Mérida | Venezuela | 23/04/1966 |  |
| *Trichobius hispidus* | *Sturnira bidens* | 2 | Tabay, Middle Refugio | Mérida | Venezuela | 6/04/1966 |  |
|  | *Sturnira bidens* | 6 | Tabay, Middle Refugio | Mérida | Venezuela | 6/04/1966 | 15/04/1966 |
|  | *Sturnira sp.* | 26 | Tabay, Middle Refugio | Mérida | Venezuela | 6/04/1966 | 15/04/1966 |
| *Xenotrichobius noctilionis* | *Noctilio albiventris* | 1 | Pto. Páez, Río Cinaruco, Hato Cariben | Apure | Venezuela | 17/09/1965 |  |
|  | *Noctilio leporinus* | 1 | Esmeralda, Caño Culebra, Belén | Amazonas | Venezuela | 12/01/1967 |  |
| *Anastrebla modestini* | *Anoura geoffroyi* | 1 | Altamira, Altamira | Barinas | Venezuela | 3/01/1968 |  |
|  | *Anoura geoffroyi* | 1 | Altamira | Barinas | Venezuela | 20/07/1967 |  |
|  | *Anoura sp.* | 2 | El Dorado, El Manaco | Bolívar | Venezuela | 13/06/1966 |  |
|  | *Anoura geoffroyi* | 3 | El Dorado, El Manaco | Bolívar | Venezuela | 23/06/1966 |  |
|  | *Anoura geoffroyi* | 13 | El Dorado | Bolívar | Venezuela | 10/05/1966 | 26/05/1966 |
|  | *Anoura sp.* | 4 | El Dorado | Bolívar | Venezuela | 10/05/1966 | 26/05/1966 |
|  | *Anoura geoffroyi* | 1 | La Paragua, Hato San José | Bolívar | Venezuela | 8/04/1967 |  |
|  | *Anoura geoffroyi* | 1 | Icabarú | Bolívar | Venezuela | 7/05/1968 |  |
|  | *Anoura sp.* | 1 | Montalbán, La Copa | Carabobo | Venezuela | 27/11/1967 | 30/11/1967 |
|  | *Anoura geoffroyi* | 1 | Montalbán, La Copa | Carabobo | Venezuela | 27/11/1967 | 30/11/1967 |
|  | *Anoura geoffroyi* | 2 | Montalbán, Potrerito | Carabobo | Venezuela | 1/11/1967 |  |
|  | *Anoura geoffroyi* | 1 | Altagracia, Hda. El Vira | Guárico | Venezuela | 16/09/1966 |  |
|  | *Anoura geoffroyi* | 2 | La Azulita, La Carbonera | Mérida | Venezuela | 21/04/1966 |  |
|  | *Anoura geoffroyi* | 1 | Tabay, Middle Refugio | Mérida | Venezuela | 15/04/1966 |  |
|  | *Anoura geoffroyi* | 1 | Caripe, nr. San Agustín | Monagas | Venezuela | 1/07/1967 |  |
|  | *Anoura geoffroyi* | 3 | Caripe, San Agustín | Monagas | Venezuela | 27/04/1967 | 3/07/1967 |
|  | *Anoura sp.* | 2 | Pto. Ayacucho, Río Manapiare, San Juan | Amazonas | Venezuela | 24/07/1967 | 27/07/1967 |
|  | *Anoura geoffroyi* | 3 | Pto. Ayacucho, Río Manapiare, San Juan | Amazonas | Venezuela | 24/07/1967 | 27/07/1967 |
|  | *Anoura geoffroyi* | 3 | Cabecera del Caño Culebra, Esmeralda | Amazonas | Venezuela | 8/02/1967 |  |
|  | *Anoura geoffroyi* | 1 | Pto. Ayacucho, Platanilla | Amazonas | Venezuela | 13/10/1967 |  |
|  | *Anoura geoffroyi* | 1 | Pto. Ayacucho, nr. Morganito | Amazonas | Venezuela | 8/10/1967 |  |
| *Anastrebla nycteridis* | *Lonchophylla robusta* | 5 | Altamira, Altamira | Barinas | Venezuela | 26/07/1967 | 4/01/1968 |
|  | *Lonchophylla robusta* | 2 | Altamira, Altamira | Barinas | Venezuela | 25/07/1967 |  |
|  | *Lonchophylla robusta* | 5 | Altamira, Altamira | Barinas | Venezuela | 21/07/1967 | 10/01/1968 |
|  | *Lonchophylla orienticolina* | 4 | Tame, Vda. Santa Inés | Arauca | Venezuela | 10/03/2019 | 15/03/2019 |
| *Anastrebla spurrelli* | *Lionycteris spurrelli* | 2 | El Dorado, El Manaco | Bolívar | Venezuela | 8/06/1966 | 25/06/1966 |
|  | *Ametrida centurio* | 1 | El Dorado | Bolívar | Venezuela | 10/05/1966 | 19/05/1966 |
|  | *Lionycteris spurrelli* | 5 | El Dorado | Bolívar | Venezuela | 10/05/1966 | 19/05/1966 |
|  | *Lionycteris spurrelli* | 15 | Icabarú, Icabarú | Bolívar | Venezuela | 09-051968 |  |
|  | *Lionycteris spurrelli* | 2 | Icabarú, Icabarú | Bolívar | Venezuela | 09-051968 |  |
|  | *Lionycteris spurrelli* | 2 | Icabarú, Icabarú | Bolívar | Venezuela | 8/05/1968 |  |
|  | *Lionycteris spurrelli* | 1 | Icabarú, Icabarú | Bolívar | Venezuela | 6/05/1968 |  |
|  | *Lionycteris spurrelli* | 16 | Icabarú, El Pauji, Icabarú | Bolívar | Venezuela | 6/05/1966 | 8/05/1968 |
|  | *Lionycteris spurrelli* | 1 | Esmeralda, Río Cunucunuma, Belén | Amazonas | Venezuela | 1/01/1967 |  |
|  | *Lionycteris spurrelli* | 1 | Cabecera del Caño Culebra, Esmeralda | Amazonas | Venezuela | 6/02/1967 |  |
|  | *Lionycteris spurrelli* | 1 | Pto. Ayacucho, Raya, Pto. Ayacucho | Amazonas | Venezuela | 12/09/1967 |  |
|  | *Lionycteris spurrelli* | 1 | Pto. Ayacucho, nr. Morganito | Amazonas | Venezuela | 4/10/1967 |  |
|  | *Lionycteris spurrelli* | 4 | Pto. Ayacucho, Río Manapiare, San Juan | Amazonas | Venezuela | 25/07/1967 |  |
| *Anastrebla caudiferae* | *Anoura caudifer* | 1 | Altamira | Barinas | Venezuela | 26/07/1967 |  |
|  | *Anoura caudifer* | 7 | Altamira, Altamira | Barinas | Venezuela | 28/07/1967 | 1/01/1968 |
|  | *Anoura caudifer* | 7 | El Dorado | Bolívar | Venezuela | 16/05/1966 | 23/05/1966 |
|  | *Anoura caudifer* | 1 | Caño Culebra, Esmeralda | Amazonas | Venezuela | 17/01/1967 |  |
| *Metalasmus sp.* | *Sturnira ludovici* | 1 | Altamira, Altamira | Barinas | Venezuela | 21/07/1967 |  |
|  | *Sturnira ludovici* | 1 | Altamira, Altamira | Barinas | Venezuela | 21/07/1967 |  |
| *Metalasmus pseudopterus* | *Artibeus planirostris* | 214 | Apure (1 locality); Barinas (2 localities); Bofivar (3 localities): Carabobo (4 localities); Dto. Federal (2 localities); Falcon (6 localities); Guarico (2 localities); Lara (1 locality); Miranda (4 localities); Monagas (1 locality); Amazonas (3 localities); Trujillo (4 localities); Yaracuy (1 locality); Zulia (7 localities). | Apure, Barinas, Bolívar, Carabobo, Dto. Federal, Falcon, Guarico, Lara, Miranda, Monagas, Amazonas, Trujillo y Zulia | Venezuela | 1960 | 1967 |
|  | *Artibeus lituratus* | 2 | Santo Domingo, Selvas de San Camilo, Nulita | Apure | Venezuela | 25/01/1968 | 5/02/1968 |
|  | *Artibeus sp.* | 2 | El Dorado, El Manaco | Bolívar | Venezuela | 20/06/1966 |  |
|  | *Myotis nigricans* | 1 | Caripe, nr. San Agustín | Monagas | Venezuela | 3/07/1967 |  |
|  | *Peropteryx macrotis* | 1 | Río Orinoco, Tamatama | Amazonas | Venezuela | 11/05/1967 |  |
|  | *Phyllostomus hastatus* | 4 | Pto. Ayacucho, Río Manapiare, San Juan | Amazonas | Venezuela | 13/07/1967 | 27/07/1967 |
|  | *Uroderma magnirostrum* | 1 | Pto. Ayacucho, Río Manapiare, San Juan | Amazonas | Venezuela | 13/07/1967 | 27/07/1967 |
|  | *Chiroderma villosum* | 1 | San Felipe, Minas de Aroa | Yaracuy | Venezuela | 22/07/1967 |  |
| *Trichobioides perspicillatus* | *Phyllostomus discolor* | 668 | Aragua (1 locality), Barinas (1 locality), Bolivar (2 localities), Carabobo (2 localities), Dto. Federal (1 locality). Falcon (4 localities), Guarico ( 1 locality), Miranda (3 locahties), Monagas (2 localities), Amazonas (4 localities), Trujillo (1 locality) | Apure, Barinas, Bolívar, Carabobo, Dto. Federal, Falcon, Guarico, Miranda, Monagas, Amazonas y Trujillo | Venezuela | 1960 | 1967 |
| *Paraeuctenodes longipes* | *Glossophaga soricina* | 4 | El Dorado, El Manaco | Bolívar | Venezuela | 9/06/1966 | 23/06/1966 |
|  | *Glossophaga soricina* | 3 | La Paragua, Hato San José | Bolívar | Venezuela | 4/04/1967 | 7/04/1967 |
|  | *Glossophaga soricina* | 1 | El Manteco, Río Supamo | Bolívar | Venezuela | 8/04/1966 |  |
|  | *Nyctinomops laticaudatus* | 1 | Esmeralda, Río Cunucunuma, Belén | Amazonas | Venezuela | 2/01/1967 | 13/01/1967 |
|  | *Glossophaga soricina* | 1 | Esmeralda, Río Cunucunuma, Belén | Amazonas | Venezuela | 2/01/1967 | 13/01/1967 |
|  | *Glossophaga soricina* | 10 | Pto. Ayacucho, Río Manapiare, San Juan | Amazonas | Venezuela | 13/07/1967 | 20-071967 |
|  | *Glossophaga soricina* | 1 | Río Orinoco, Tamatama | Amazonas | Venezuela | 4/05/1967 |  |
|  | *Glossophaga soricina* | 1 | San Felipe, Minas de Aroa | Yaracuy | Venezuela | 12/12/1968 |  |
|  | *Artibeus lituratus* | 1 | El Dorado | Bolívar | Venezuela | 2/08/1962 |  |
| *Paraeuctenodes similis* | *Carollia perspicillata* | 1 | Icabarú, Icabarú | Bolívar | Venezuela | 8/05/1968 |  |
|  | *Carollia perspicillata* | 1 | El Dorado | Bolívar | Venezuela | 13/05/1966 |  |
| *Strebla altmani* | *Lonchorhina orinocensis* | 71 | Pto. Páez, La Villa, Hato Cariben | Apure | Venezuela | 6/07/1965 | 28/07/1965 |
|  | *Lonchorhina orinocensis* | 3 | Pto. Páez, Cerro de Murcielagos, Pto. Páez | Apure | Venezuela | 19/01/1966 | 24/01/1966 |
|  | *Lonchorhina aurita* | 3 | Altamira, Altamira | Barinas | Venezuela | 25/07/1967 |  |
|  | *Lonchorhina aurita* | 2 | La Paragua, Hato San José | Bolívar | Venezuela | 8/04/1967 |  |
|  | *Lonchorhina orinocensis* | 3 | Pto. Ayacucho, El Gavilan | Amazonas | Venezuela | 11/10/1967 |  |
|  | *Lonchorhina orinocensis* | 2 | Pto. Ayacucho, Paria | Amazonas | Venezuela | 13/09/1967 | 20/09/1967 |
|  | *Lonchorhina orinocensis* | 1 | Pto. Ayacucho, Coromoto | Amazonas | Venezuela | 8/10/1967 |  |
|  | *Lonchorhina aurita* | 3 | Esmeralda, Río Mavaca | Amazonas | Venezuela | 2/03/1967 |  |
|  | *Macrophyllum macrophyllum* | 1 | Pto. Ayacucho, Río Manapiare, San Juan | Amazonas | Venezuela | 25/07/1967 |  |
|  | *Lonchorhina aurita* | 47 | Valera, nr. Agua Viva | Trujillo | Venezuela | 3/09/1965 |  |
|  | *Lonchorhina aurita* | 7 | Valera, nr. Agua Viva | Trujillo | Venezuela | 30/08/1965 |  |
|  | *Lonchorhina aurita* | 1 | Valera, Río Motatan | Trujillo | Venezuela | 8/10/1965 |  |
|  | *Lonchorhina aurita* | 3 | Valera, nr. Agua Santa | Trujillo | Venezuela | 18-091965 |  |
|  | *Lonchorhina aurita* | 21 | Valera, Quebrada Seca | Trujillo | Venezuela | 21/10/1965 |  |
|  | *Lonchorhina aurita* | 3 | San Felipe, Minas de Aroa | Yaracuy | Venezuela | 6/07/1967 | 23/07/1967 |
| *Strebla alvarezi* | *Micronycteris microtis* | 4 | Icabarú, El Pauji, Icabarú | Bolívar | Venezuela | 3/05/1968 |  |
|  | *Carollia brevicauda* | 1 | El Dorado, El Manaco | Bolívar | Venezuela | 9/06/1966 |  |
|  | *Lonchophylla thomasi* | 1 | Pto. Ayacucho, Paria | Amazonas | Venezuela | 17/09/1967 |  |
|  | *Micronycteris microtis* | 1 | Esmeralda, Boca Mavaca | Amazonas | Venezuela | 21/02/1966 |  |
|  | *Micronycteris megalotis* | 1 | Urama, El Central | Yaracuy | Venezuela | 8/03/1966 |  |
| *Strebla asternalis* | *Saccopteryx bilineata* | 2 | Esmeralda, Río Mavaca | Amazonas | Venezuela | 5/04/1967 |  |
|  | *Saccopteryx bilineata* | 10 | Esmeralda, Río Mavaca | Amazonas | Venezuela | 10/03/1967 |  |
|  | *Saccopteryx sp.* | 7 | Esmeralda, Río Mavaca | Amazonas | Venezuela | 10/03/1967 |  |
|  | *Saccopteryx bilineata* | 4 | Esmeralda, Río Cunucunuma, Belén | Amazonas | Venezuela | 3/01/1967 |  |
| *Strebla choropteri* | *Chrotopterus auritus* | 5 | Icabarú, Santa Lucia de Surukun | Bolívar | Venezuela | 1/05/1968 |  |
|  | *Chrotopterus auritus* | 5 | Esmeralda, Río Cunucunuma, Belén | Amazonas | Venezuela | 9/02/1967 |  |
|  | *Chrotopterus auritus* | 18 | Esmeralda, Río Mavaca | Amazonas | Venezuela | 3/04/1967 | 14/04/1967 |
|  | *Chrotopterus auritus* | 13 | Pto. Ayacucho, Río Manapiare, San Juan | Amazonas | Venezuela | 26/07/1967 |  |
| *Strebla christinae* | *Phylloderma stenops* | 16 | Pto. Páez, Río Cinaruco, Hato Cariben | Apure | Venezuela | 16/07/1965 |  |
|  | *Phylloderma stenops* | 12 | La Paragua, Hato San José | Bolívar | Venezuela | 6/04/1967 |  |
|  | *Eumops glaucinus* | 1 | Pto. Ayacucho, Río Manapiare, San Juan | Amazonas | Venezuela | 13/07/1967 | 28/07/1967 |
|  | *Phylloderma stenops* | 39 | Pto. Ayacucho, Río Manapiare, San Juan | Amazonas | Venezuela | 13/07/1967 | 28/07/1967 |
|  | *Uroderma magnirostrum* | 1 | Río Orinoco, Tamatama | Amazonas | Venezuela | 20/04/1967 | 15/05/1967 |
|  | *Phylloderma stenops* | 9 | Río Orinoco, Tamatama | Amazonas | Venezuela | 20/04/1967 | 15/05/1967 |
|  | *Phylloderma stenops* | 51 | Esmeralda, Río Cunucunuma, Belén | Amazonas | Venezuela | 2/01/1967 | 4/01/1967 |
|  | *Phylloderma stenops* | 17 | Esmeralda, Brazo Casiquiare, Capibara | Amazonas | Venezuela | 6/06/1967 |  |
|  | *Phylloderma stenops* | 10 | Pto. Ayacucho, El Raudal | Amazonas | Venezuela | 20/09/1967 |  |
|  | *Phylloderma stenops* | 36 | Esmeralda, Río Mavaca | Amazonas | Venezuela | 3/04/1967 | 14/04/1967 |
| *Strebla consocia* | *Phyllostomus hastatus* | 6 | Santo Domingo, Selvas de San Camilo, Nulita | Apure | Venezuela | 30/01/1968 | 31/01/1968 |
|  | *Phyllostomus elongatus* | 1 | Pto. Páez, La Villa, Hato Cariben | Apure | Venezuela | 24/07/1965 |  |
|  | *Phyllostomus hastatus* | 3 | Altamira, Altamira | Barinas | Venezuela | 26/12/1967 |  |
|  | *Phyllostomus hastatus* | 8 | Altamira | Barinas | Venezuela | 21/12/1967 |  |
|  | *Platyrrhinus helleri* | 1 | Altamira | Barinas | Venezuela | 21/12/1967 |  |
|  | *Phyllostomus elongatus* | 1 | El Manteco, Los Patos | Bolívar | Venezuela | 5/04/1966 |  |
|  | *Phyllostomus elongatus* | 8 | El Manteco, Río Supamo | Bolívar | Venezuela | 11/04/1966 |  |
|  | *Phyllostomus hastatus* | 1 | El Dorado, El Manaco | Bolívar | Venezuela | 17/06/1966 |  |
|  | *Phyllostomus hastatus* | 2 | El Manteco, Los Patos | Bolívar | Venezuela | 11/04/1966 |  |
|  | *Phyllostomus hastatus* | 1 | Montalbán, Potrerito | Carabobo | Venezuela |  |  |
|  | *Phyllostomus elongatus* | 1 | Maturín, Hato Mata de Bejuco | Monagas | Venezuela | 3/06/1968 | 4/06/1968 |
|  | *Phyllostomus hastatus* | 6 | Maturín, Hato Mata de Bejuco | Monagas | Venezuela | 3/06/1968 | 4/06/1968 |
|  | *Phyllostomus hastatus* | 8 | Caripe, nr. San Agustín | Monagas | Venezuela | 11/07/1967 |  |
|  | *Phyllostomus hastatus* | 10 | Caripe, San Agustín | Monagas | Venezuela | 26/06/1967 |  |
|  | *Phyllostomus elongatus* | 3 | Esmeralda, Boca Mavaca | Amazonas | Venezuela | 20/02/1967 | 24/03/1967 |
|  | *Phyllostomus hastatus* | 5 | Esmeralda, Boca Mavaca | Amazonas | Venezuela | 20/02/1967 | 24/03/1967 |
|  | *Phyllostomus hastatus* | 29 | Esmeralda, Brazo Casiquiare, Capibara | Amazonas | Venezuela | 30/05/1967 | 7/06/1967 |
|  | *Phyllostomus elongatus* | 2 | Esmeralda, Brazo Casiquiare, Capibara | Amazonas | Venezuela | 30/05/1967 | 7/06/1967 |
|  | *Phyllostomus elongatus* | 3 | Pto. Ayacucho, Raya, Pto. Ayacucho | Amazonas | Venezuela | 6/09/1967 | 10/10/1967 |
|  | *Phyllostomus hastatus* | 7 | Pto. Ayacucho, Raya, Pto. Ayacucho | Amazonas | Venezuela | 6/09/1967 | 10/10/1967 |
|  | *Phyllostomus elongatus* | 59 | Esmeralda, Río Mavaca | Amazonas | Venezuela | 3/04/1967 | 14/04/1967 |
|  | *Trachops cirrhosus* | 3 | Esmeralda, Río Mavaca | Amazonas | Venezuela | 3/04/1967 | 14/04/1967 |
|  | *Phyllostomus hastatus* | 44 | Pto. Ayacucho, Río Manapiare, San Juan | Amazonas | Venezuela | 5/07/1967 | 27/07/1967 |
|  | *Phyllostomus elongatus* | 29 | Pto. Ayacucho, Río Manapiare, San Juan | Amazonas | Venezuela | 5/07/1967 | 27/07/1967 |
|  | *Desmodus rotundus* | 2 | Pto. Ayacucho, Río Manapiare, San Juan | Amazonas | Venezuela | 5/07/1967 | 27/07/1967 |
|  | *Phyllostomus elongatus* | 15 | Río Orinoco, Tamatama | Amazonas | Venezuela | 28/04/1967 | 8/05/1967 |
|  | *Phyllostomus hastatus* | 3 | Río Orinoco, Tamatama | Amazonas | Venezuela | 28/04/1967 | 8/05/1967 |
|  | *Phyllostomus elongatus* | 3 | Esmeralda, Río Cunucunuma, Belén | Amazonas | Venezuela | 16/02/1967 |  |
|  | *Phyllostomus hastatus* | 2 | Pto. Ayacucho, El Raudal | Amazonas | Venezuela | 20/09/1967 |  |
|  | *Phyllostomus hastatus* | 11 | Valera, Río Motatan | Trujillo | Venezuela | 2/09/1965 |  |
|  | *Phyllostomus hastatus* | 3 | Urama, El Central | Yaracuy | Venezuela | 14/03/1966 | 22/03/1966 |
| *Strebla cormurae* | *Cormura brevirostris* | 2 | Esmeralda, Río Cunucunuma, Belén | Amazonas | Venezuela | 19/01/1967 |  |
| *Strebla curvata* | *Glossophaga soricina* | 1 | El Dorado, El Manaco | Bolívar | Venezuela | 13/06/1966 |  |
|  | *Glossophaga soricina* | 1 | El Dorado, El Manaco | Bolívar | Venezuela | 16/06/1966 |  |
|  | *Noctilio albiventris* | 1 | Pto. Páez, Río Cinaruco, Hato Cariben | Apure | Venezuela | 14/07/1965 |  |
|  | *Glossophaga longirostris* | 4 | Pto. Páez, La Villa, Hato Cariben | Apure | Venezuela | 6/12/1967 | 9/12/1967 |
|  | *Carollia brevicauda* | 1 | Altamira, Altamira | Barinas | Venezuela | 27/12/1967 | 28/12/1967 |
|  | *Carollia perspicillata* | 4 | Altamira, Altamira | Barinas | Venezuela | 27/12/1967 | 28/12/1967 |
|  | *Glossophaga soricina* | 1 | El Dorado, El Manaco | Bolívar | Venezuela | 16/06/1966 |  |
|  | *Glossophaga soricina* | 4 | El Dorado, El Manaco | Bolívar | Venezuela | 13/06/1966 | 21/06/1966 |
|  | *Glossophaga soricina* | 3 | La Paragua, Hato San José | Bolívar | Venezuela | 4/04/1967 | 7/04/1967 |
|  | *Glossophaga soricina* | 1 | Maturín, Hato Mata de Bejuco | Monagas | Venezuela | 3/06/1968 |  |
|  | *Glossophaga longirostris* | 1 | Pto. Ayacucho, Las Queseras | Amazonas | Venezuela | 27/09/1967 |  |
|  | *Glossophaga longirostris* | 2 | Pto. Ayacucho, nr. Morganito | Amazonas | Venezuela | 4/10/1967 |  |
|  | *Glossophaga soricina* | 6 | Esmeralda, Río Cunucunuma, Belén | Amazonas | Venezuela | 2/01/1967 | 3/01/1967 |
|  | *Glossophaga soricina* | 10 | Pto. Ayacucho, Río Manapiare, San Juan | Amazonas | Venezuela | 6/07/1967 | 27/07/1967 |
|  | *Glossophaga soricina* | 3 | Río Orinoco, Tamatama | Amazonas | Venezuela | 2/05/1967 | 4/05/1967 |
| *Strebla diaemi* | *Diaemus youngii* | 7 | Pto. Ayacucho, Chaparito | Amazonas | Venezuela | 2/10/1967 |  |
|  | *Diaemus youngii* | 18 | Pto. Ayacucho, Guayabal | Amazonas | Venezuela | 7/10/1967 |  |
|  | *Diaemus youngii* | 42 | Pto. Ayacucho, Río Manapiare, San Juan | Amazonas | Venezuela | 7/10/1967 |  |
| *Strebla diphyllae* | *Diphylla ecaudata* | 11 | Rancho Grande, El Limón | Aragua | Venezuela |  |  |
| *Strebla galindoi* | *Tonatia bidens* | 2 | Pto. Páez, Río Cinaruco, Hato Cariben | Apure | Venezuela | 14/07/1965 |  |
|  | *Tonatia bidens* | 1 | Santo Domingo, Selvas de San Camilo, Nulita | Apure | Venezuela | 24/01/1968 |  |
|  | *Tonatia bidens* | 2 | El Dorado, El Manaco | Bolívar | Venezuela | 22/06/1966 |  |
|  | *Tonatia bidens* | 2 | Esmeralda, Río Mavaca | Amazonas | Venezuela | 10/04/1967 |  |
| *Strebla guajiro* | *Carollia perspicillata* | 523 | Apure (3 localities); Barinas (5 localities); Bolivar (10 localities); Carabobo (6 localities); Falcon (8 localities); Guarico (2 localities); Miranda (5 localities); Monagas (4 localities); Amazonas (14 localities); Trujillo (4 localities); Yaracuy (2 localities); and Zulia (8 localities). | Apure, Bolívar, Carabobo, Falcon, Guarico, Miranda, Monagas, Amazonas, Trujillo, Yaracuy and Zulia | Venezuela | 1960 | 1967 |
|  | *Carollia perspicillata* | 1 | Rancho Grande, El Limón | Aragua | Venezuela | 30/03/1960 |  |
|  | *Carollia perspicillata* | 4 | Guacharo Cave | Monagas | Venezuela | 16/08/1962 |  |
|  | *Carollia brevicauda* | 3 | Reserva Natural La Palmita | Casanare | Colombia | 2017 |  |
|  | *Carollia perspicillata* | 4 | Reserva Natural La Palmita | Casanare | Colombia | 2017 |  |
| *Strebla harderi* | *Anoura geoffroyi* | 2 | Pto. Ayacucho, Río Manapiare, San Juan | Amazonas | Venezuela | 27/07/1967 |  |
|  | *Anoura sp.* | 1 | El Dorado | Bolívar | Venezuela | 18/05/1966 |  |
|  | *Anoura geoffroyi* | 1 | El Dorado, El Manaco | Bolívar | Venezuela | 13/06/1966 |  |
|  | *Anoura geoffroyi* | 2 | Cabecera del Caño Culebra, Esmeralda | Amazonas | Venezuela | 7/02/1967 | 8/02/1967 |
|  | *Anoura geoffroyi* | 2 | Pto. Ayacucho, Río Manapiare, San Juan | Amazonas | Venezuela | 27/07/1967 |  |
| *Strebla hertigi* | *Phyllostomus discolor* | 6 | Altamira, Altamira | Barinas | Venezuela | 26/07/1967 | 2/01/1968 |
|  | *Phyllostomus discolor* | 10 | La Paragua, Hato San José | Bolívar | Venezuela | 10/04/1967 |  |
|  | *Phyllostomus discolor* | 2 | La Paragua, Hato San José | Bolívar | Venezuela | 10/04/1967 |  |
|  | *Phyllostomus discolor* | 52 | Montalbán, Potrerito | Carabobo | Venezuela | 31/10/1967 | 1/11/1967 |
|  | *Phyllostomus discolor* | 1 | Altagracia, Hda. El Vira | Guárico | Venezuela | 16/09/1966 |  |
|  | *Phyllostomus discolor* | 7 | San Juan de los Morros, Hato. Las Palmitas | Guárico | Venezuela | 4/09/1966 |  |
|  | *Phyllostomus discolor* | 4 | Maturín, Hato Mata de Bejuco | Monagas | Venezuela | 3/04/1968 |  |
|  | *Phyllostomus discolor* | 3 | Caripe, San Agustín | Monagas | Venezuela | 28/06/1967 |  |
|  | *Phyllostomus discolor* | 16 | Pto. Ayacucho, Coromoto | Amazonas | Venezuela | 11/09/1967 |  |
|  | *Phyllostomus discolor* | 15 | Pto. Ayacucho, El Raudal | Amazonas | Venezuela | 19/09/1967 | 20/09/1967 |
|  | *Phyllostomus discolor* | 20 | Esmeralda, Río Mavaca | Amazonas | Venezuela | 3/04/1967 | 14/04/1967 |
|  | *Phyllostomus discolor* | 31 | Pto. Ayacucho, Río Manapiare, San Juan | Amazonas | Venezuela | 13/07/1967 | 27/07/1967 |
|  | *Phyllostomus discolor* | 1 | Valera, nr. Agua Viva | Trujillo | Venezuela | 23/10/1965 |  |
|  | *Phyllostomus discolor* | 9 | Valera, nr. Agua Santa | Trujillo | Venezuela | 7/09/1965 | 22/10/1965 |
|  | *Phyllostomus elongatus* | 5 | Tame, Vda. Santa Inés | Arauca | Colombia | 12/03/2019 |  |
|  | *Phyllostomus discolor* | 2 | Tame, Vda. Santa Inés | Arauca | Colombia | 15/03/2019 |  |
| *Strebla kohlsi* | *Lophostoma silvicola* | 1 | Esmeralda, Río Cunucunuma, Belén | Amazonas | Venezuela | 3/01/1967 |  |
|  | *Lophostoma silvicola* | 7 | Esmeralda, Brazo Casiquiare, Capibara | Amazonas | Venezuela | 30/05/1967 | 12/06/1967 |
| *Strebla machadoi* | *Micronycteris minuta* | 1 | Santo Domingo, Selvas de San Camilo, Nulita | Apure | Venezuela | 31/01/1968 |  |
|  | *Micronycteris minuta* | 1 | El Manteco, Los Patos | Bolívar | Venezuela | 11/04/1966 |  |
|  | *Micronycteris minuta* | 1 | Maturín, Hato Mata de Bejuco | Monagas | Venezuela | 3/06/1968 |  |
|  | *Micronycteris minuta* | 3 | Pto. Ayacucho, nr. Morganito | Amazonas | Venezuela | 4/10/1967 |  |
| *Strebla matsoni* | *Macrophyllum macrophyllum* | 1 | Pto. Páez, La Villa, Hato Caribean | Apure | Venezuela | 6/07/1965 |  |
|  | *Rhynchonycteris naso* | 1 | El Dorado, El Manaco | Bolívar | Venezuela | 10/06/1966 | 24/06/1966 |
|  | *Macrophyllum macrophyllum* | 4 | El Dorado, El Manaco | Bolívar | Venezuela | 10/06/1966 | 24/06/1966 |
|  | *Macrophyllum macrophyllum* | 1 | El Manteco, Río Supamo | Bolívar | Venezuela | 11/04/1966 |  |
|  | *Macrophyllum macrophyllum* | 4 | Esmeralda, Río Cunucunuma | Amazonas | Venezuela | 10/02/1967 |  |
|  | *Macrophyllum macrophyllum* | 7 | Esmeralda, Río Mavaca | Amazonas | Venezuela | 5/04/1967 | 10/04/1967 |
| *Strebla mirabilis* | *Phyllostomus elongatus* | 2 | Pto. Páez, La Villa, Hato Caribean | Apure | Venezuela | 23/07/1965 | 28/07/1965 |
|  | *Trachops cirrhosus* | 34 | Pto. Páez, La Villa, Hato Caribean | Apure | Venezuela | 23/07/1965 | 28/07/1965 |
|  | *Trachops cirrhosus* | 1 | Pto. Páez, Río Cinaruco | Apure | Venezuela | 27/07/1965 |  |
|  | *Phyllostomus elongatus* | 1 | El Dorado, El Manaco | Bolívar | Venezuela | 13/06/1966 | 23/06/1966 |
|  | *Trachops cirrhosus* | 14 | El Dorado, El Manaco | Bolívar | Venezuela | 13/06/1966 | 23/06/1966 |
|  | *Trachops cirrhosus* | 1 | Icabarú, Santa Lucia de Surukun | Bolívar | Venezuela | 1/05/1968 | 2/05/1968 |
|  | *Phyllostomus hastatus* | 8 | Icabarú, Santa Lucia de Surukun | Bolívar | Venezuela | 1/05/1968 | 2/05/1968 |
|  | *Phyllostomus elongatus* | 1 | El Dorado, Piedra Virgen | Bolívar | Venezuela | 29/05/1966 |  |
|  | *Trachops cirrhosus* | 26 | La Paragua, Hato San José | Bolívar | Venezuela | 6/03/1967 | 10/04/1967 |
|  | *Trachops cirrhosus* | 2 | El Dorado | Bolívar | Venezuela | 9/05/1966 |  |
|  | *Trachops cirrhosus* | 2 | El Manteco, Río Supamo | Bolívar | Venezuela | 11/04/1966 |  |
|  | *Trachops cirrhosus* | 5 | Calabozo, nr. Río Orituco | Guárico | Venezuela | 21/08/1968 | 22/08/1968 |
|  | *Chrotopterus auritus* | 1 | Esmeralda, Río Mavaca | Amazonas | Venezuela | 3/04/1967 | 14/04/1967 |
|  | *Phyllostomus elongatus* | 2 | Esmeralda, Río Mavaca | Amazonas | Venezuela | 3/04/1967 | 14/04/1967 |
|  | *Trachops cirrhosus* | 59 | Esmeralda, Río Mavaca | Amazonas | Venezuela | 3/04/1967 | 14/04/1967 |
|  | *Artibeus planirostris* | 1 | Esmeralda, Caño Culebra, Belén | Amazonas | Venezuela | 12/01/1967 |  |
|  | *Chrotopterus auritus* | 1 | Pto. Ayacucho, Río Manapiare, San Juan | Amazonas | Venezuela | 5/07/1967 | 27/07/1967 |
|  | *Trachops cirrhosus* | 17 | Pto. Ayacucho, Río Manapiare, San Juan | Amazonas | Venezuela | 5/07/1967 | 27/07/1967 |
|  | *Trachops cirrhosus* | 3 | Esmeralda,Boca Mavaca | Amazonas | Venezuela | 20/02/1966 |  |
|  | *Trachops cirrhosus* | 2 | Esmeralda, Brazo Casiquiare, Capibara | Amazonas | Venezuela | 30/05/1967 | 2/06/1967 |
|  | *Trachops cirrhosus* | 10 | Pto. Ayacucho, El Gavilan | Amazonas | Venezuela | 11/10/1967 |  |
|  | *Trachops cirrhosus* | 1 | Pto. Ayacucho, Raya | Amazonas | Venezuela | 7/09/1967 |  |
|  | *Trachops cirrhosus* | 4 | Pto. Ayacucho, El Raudal | Amazonas | Venezuela | 20/09/1967 | 5/10/1967 |
|  | *Trachops cirrhosus* | 5 | Urama, El Central | Yaracuy | Venezuela | 15/03/1966 |  |
|  | *Diphylla ecaudata* | 6 | Rancho Grande, El Limón | Aragua | Venezuela | 30/03/1960 |  |
| *Strebla obtusa* | *Trinycteris nicefori* | 2 | El Manteco, Los Patos | Bolívar | Venezuela | 5/04/1966 |  |
|  | *Trinycteris nicefori* | 18 | El Manteco, Los Patos | Bolívar | Venezuela | 5/04/1966 |  |
|  | *Phyllostomus elongatus* | 2 | El Manteco, Los Patos | Bolívar | Venezuela | 5/04/1966 |  |
|  | *Trinycteris nicefori* | 2 | Esmeralda, Boca Mavaca | Amazonas | Venezuela | 14/03/1967 |  |
|  | *Trinycteris nicefori* | 2 | Pto. Ayacucho, Paria | Amazonas | Venezuela | 19/09/1967 |  |
|  | *Trinycteris nicefori* | 1 | Esmeralda, Río Mavaca | Amazonas | Venezuela | 10/04/1967 |  |
|  | *Trinycteris nicefori* | 3 | Río Orinoco, Tamatama | Amazonas | Venezuela | 21/04/1967 |  |
| *Strebla paramirabilis* | *Artibeus planirostris* | 2 | Cabecera del Caño Culebra, Esmeralda | Amazonas | Venezuela | 2/02/1967 | 7/02/1967 |
|  | *Platyrrhinus aurarius* | 6 | El Dorado | Bolívar | Venezuela | 5/05/1966 | 26/05/1966 |
|  | *Artibeus planirostris* | 1 | El Dorado | Bolívar | Venezuela | 5/05/1966 | 26/05/1966 |
|  | *Artibeus planirostris* | 2 | Icabarú, El Pauji, Icabarú | Bolívar | Venezuela | 5/05/1968 | 7/05/1968 |
|  | *Anoura geoffroyi* | 1 | Cabecera del Caño Culebra, Esmeralda | Amazonas | Venezuela | 2/02/1967 | 7/02/1967 |
|  | *Artibeus planirostris* | 12 | Cabecera del Caño Culebra, Esmeralda | Amazonas | Venezuela | 2/02/1967 | 7/02/1967 |
|  | *Platyrrhinus aurarius* | 6 | Caño Culebra, Esmeralda | Amazonas | Venezuela | 11/01/1967 | 19/01/1967 |
|  | *Artibeus planirostris* | 2 | Caño Culebra, Esmeralda | Amazonas | Venezuela | 11/01/1967 | 19/01/1967 |
|  | *Artibeus planirostris* | 4 | Esmeralda, Río Cunucunuma | Amazonas | Venezuela | 2/02/1967 |  |
| *Strebla proxima* | *Peropteryx macrotis* | 2 | Pto. Ayacucho, Río Manapiare, San Juan | Amazonas | Venezuela | 18/07/1967 |  |
|  | *Peropteryx macrotis* | 11 | San Felipe, Minas de Aroa | Yaracuy | Venezuela | 14/07/1967 | 16/07/1967 |
|  | *Peropteryx trinitatis* | 2 | San Felipe, Minas de Aroa | Yaracuy | Venezuela | 14/07/1967 | 16/07/1967 |
| *Strebla tonatiae* | *Sturnira lilium* (probably *S. giannae*) | 1 | Santo Domingo, Selvas de San Camilo, Nulita | Apure | Venezuela | 17/01/1968 | 1/02/1968 |
|  | *Lophostoma brasiliense* | 18 | Santo Domingo, Selvas de San Camilo, Nulita | Apure | Venezuela | 17/01/1968 | 1/02/1968 |
|  | *Lophostoma brasiliense* | 1 | El Dorado, El Manaco | Bolívar | Venezuela | 13/06/1966 |  |
|  | *Lophostoma brasiliense* | 4 | Maturín, Hato Mata de Bejuco | Monagas | Venezuela | 5/08/1966 |  |
|  | *Lophostoma carrikeri* | 2 | Pto. Ayacucho, Río Manapiare, San Juan | Amazonas | Venezuela | 24/07/1967 | 28/07/1967 |
|  | *Lophostoma brasiliense* | 16 | Pto. Ayacucho, Río Manapiare, San Juan | Amazonas | Venezuela | 24/07/1967 | 28/07/1967 |
|  | *Lophostoma brasiliense* | 1 | Pto. Ayacucho, nr. Morganito | Amazonas | Venezuela | 8/10/1967 |  |
|  | *Lophostoma brasiliense* | 9 | Valera, nr. Agua Viva | Trujillo | Venezuela | 15/09/1965 |  |
|  | *Lophostoma brasiliense* | 1 | Urama, El Central | Yaracuy | Venezuela | 15/03/1966 |  |
|  | *Tonatia saurophila* | 3 | Reserva Natural La Palmita | Casanare | Venezuela | 2017 |  |
| *Strebla wiedemanni* | *Desmodus rotundus* | 1937 | Bolivar (2 localities); Carabobo (5 localities); Dto. Federal (1 locality); Falcon (5 localities); Guarico (4 locahties); Lara (1 locality); Miranda (6 localities); Monagas (3 localities); Amazonas (9 localities); Trujillo (6 localities); Yaracuy (1 locality); and Zulia (3 localities). | Bolívar, Carabobo, Dt. Federal, Guarico, Larar, Miranda, Monagas, Amazonas, Trujillo, Yaracuy and Zulia | Venezuela | 1966 | 1967 |
|  | *Desmodus rotundus* | 2 | Caripe | Monagas | Venezuela | 1966 | 1967 |
| *Basilia wenzeli* | *Eptesicus fuscus* | 1 | Rancho Grande, El Limón | Aragua | Venezuela | 1966 | 1967 |
|  | *Lonchorhina aurita* | 1 | Rancho Grande, El Limón | Aragua | Venezuela | 1966 | 1967 |
| *Basilia tiptoni* | *Gardnerycteris crenulatum* | 2 | Valera, nr. Agua Viva | Trujillo | Venezuela | 1966 | 1967 |
|  | *Gardnerycteris crenulatum* | 1 | Pto. Páez, nr. Hato Caribean, Río Cinaruco | Apure | Venezuela | 1966 | 1967 |
| *Basilia ortizi* | *Eptesicus brasiliensis* | 5 | Esmeralda, Boca Mavaca | Amazonas | Venezuela | 13/02/1966 |  |
|  | *Eptesicus furinalis* | 2 | Ciudad Bolívar, Hato San José | Bolívar | Venezuela | 6/04/1967 |  |
|  | *Eptesicus furinalis* | 1 | Maturín, Hato Santa Barbara | Monagas | Venezuela | 8/08/1966 |  |
|  | *Eptesicus furinalis* | 1 | Tamatama, Río Orinuco | Amazonas | Venezuela | 26/04/1967 |  |
|  | *Eptesicus brasiliensis* | 5 | Tamatama, Río Orinuco | Amazonas | Venezuela | 28/04/1967 |  |
|  | *Eptesicus brasiliensis* | 2 | Tamatama, Río Orinuco | Amazonas | Venezuela | 28/04/1967 |  |
|  | *Myotis riparius* | 1 | Casiquiare Canal, Capibara | Amazonas | Venezuela | 8/04/1967 |  |
|  | *Eptesicus brasiliensis* | 1 | Maracay, Río Manapiare | Amazonas | Venezuela | 17/07/1967 |  |
|  | *Eptesicus brasiliensis* | 5 | Pto. Ayacucho, Río Manapiare, San Juan | Amazonas | Venezuela | 27/07/1967 |  |
|  | *Eptesicus orinocensis* | 9 | Arauca | Arauca | Colombia |  |  |
| *Basilia juquiensis* | *Myotis riparius* | 2 | Nula, La Chiricoa | Apure | Venezuela | 30/01/1968 |  |
|  | *Myotis riparius* | 1 | Nula, La Chiricoa | Apure | Venezuela | 31/01/1968 |  |
| *Basilia dubia* | *Myotis albescens* | 1 | Pto. Páez, Río Cinaruco | Apure | Venezuela | 25/01/1966 |  |
|  | *Myotis albescens* | 1 | Pto. Páez, Río Cinaruco | Apure | Venezuela | 25/01/1966 |  |
|  | *Myotis albescens* | 3 | Esmeralda, Río Mavaca | Amazonas | Venezuela | 3/03/1967 |  |
|  | *Saccopteryx bilineata* | 1 | Nula, Nulita | Apure | Venezuela | 17/01/1968 |  |
|  | *Myotis albescens* | 5 | Nula, Nulita | Apure | Venezuela | 17/01/1968 |  |
| *Basilia tuttlei* | *Myotis nigricans* | 2 | Río Cunucunuma, Belén | Amazonas | Venezuela | 2/02/1967 |  |
| *Basilia typhlops* | *Myotis oxyotus* | 2 | El Dorado | Bolívar | Venezuela | 16/05/1966 |  |
|  | *Myotis oxyotus* | 1 | Icabarú, El Mundo Nuevo de Surukun | Bolívar | Venezuela | 3/05/1968 |  |
| *Basilia ferrisi* | *Myotis nigricans* | 2 | Caripe, Hda. San Fernando | Monagas | Venezuela | 4/07/1967 |  |
|  | *Myotis albescens* | 1 | Caripe, Hda. San Fernando | Monagas | Venezuela | 5/07/1967 |  |
|  | *Desmodus rotundus* | 1 | Caripe, Hda. Tucuseto | Monagas | Venezuela | 13/07/1967 |  |
|  | *Myotis nigricans* | 2 | Pto. Ayacucho, Paria | Amazonas | Venezuela | 4/10/1967 |  |
|  | *Myotis riparius* | 2 | Nula, Nulita | Apure | Venezuela | 17/01/1968 |  |
|  | *Myotis handleyi* | 2 | Arauca, Vda. El Socorro | Arauca | Colombia | 28/07/2019 |  |
|  | *Molossus pretiossus* | 2 | Arauca, Vda. El Socorro | Arauca | Colombia | 28/07/2019 |  |
|  | *Myotis nigricans* | 5 | Cravo Norte, Vereda Las plmas, El deleite | Arauca | Colombia | 10/11/2018 | 30/11/2018 |
|  | *Myotis nigricans* | 3 | Arauca, Vda. Las plumas | Arauca | Colombia | 10/11/2018 | 30/11/2018 |
|  | *Noctilio albiventris* | 1 | Cravo Norte, Vereda Las plmas, El deleite | Arauca | Colombia | 26/11/2018 |  |
|  | *Phyllostomus hastatus* | 1 | Arauca, Sede Universidad Nacional | Arauca | Colombia | 27/07/2019 |  |
|  | *Platyrrhinus helleri* | 6 | Arauca, Vda. Las plumas | Arauca | Colombia | 30/11/2018 |  |

**Table S3.** Centrality metric of the species of ectoparasitic flies.

| **Species ectoparasitic flies** | **Degree centrality (DC)** |
| --- | --- |
| *Mastoptera guimaraesi* | 1 |
| *Anastrebla caudiferae* | 1 |
| *Anastrebla mattadeni* | 1 |
| *Anastrebla modestini* | 2 |
| *Anastrebla nycteridis* | 2 |
| *Anastrebla spurrelli* | 2 |
| *Anatrichobius scorzai* | 2 |
| *Aspidoptera delatorrei* | 5 |
| *Aspidoptera falcata* | **9** |
| *Aspidoptera phyllostomatis* | 5 |
| *Basilia dubia* | 2 |
| *Basilia ferrisi* | 6 |
| *Basilia juquiensis* | 1 |
| *Basilia myotis* | 6 |
| *Basilia ortizi* | 5 |
| *Basilia tiptoni* | 1 |
| *Basilia tuttlei* | 1 |
| *Basilia typhlops* | 1 |
| *Basilia wenzeli* | 2 |
| *Exastinion clovisi* | 5 |
| *Exastinion deceptivum* | 1 |
| *Exastinion oculatum* | 1 |
| *Mastoptera minuta* | **11** |
| *Megistopoda aranea* | 3 |
| *Megistopoda proxima* | 4 |
| *Metalasmus pseudopterus* | **8** |
| *Metalasmus* sp. | 1 |
| *Neotrichobius bisetosus* | 2 |
| *Neotrichobius delicatus* | 4 |
| *Neotrichobius ectophyllae* | 1 |
| *Neotrichobius stenopterus* | 1 |
| *Noctiliostrebla aitkeni* | 2 |
| *Noctiliostrebla maai* | 3 |
| *N. coxata* | 3 |
| *Nycterophilia parnelli* | 3 |
| *Paradyschiria curvata* | 4 |
| *Paradyschiria fusca* | 1 |
| ***Paradyschiria lineata*** | 3 |
| *Paradyschiria parvula* | 3 |
| *Paradyschiria parvuloides* | 2 |
| *Paraeuctenoides longipes* | 4 |
| *Paraeuctenoides similis* | 1 |
| *Parastrebla handleyi* | 1 |
| *Paratrichobius dunni* | 2 |
| *Paratrichobius longicrus* | 3 |
| *Paratrichobius lowei* | 1 |
| *Paratrichobius salvini* | 6 |
| *Paratrichobius sanchezi* | 1 |
| *Phalcophila puliciformis* | 1 |
| *Pseudostrebla greenwelli* | 2 |
| *Pseudostrebla ribeiroi* | 1 |
| *Pseudostrebla sparsisetis* | 1 |
| *Speiseria ambigua* | 2 |
| *Speiseria magnioculus* | 1 |
| *Speseria peytoni* | 1 |
| *Stizostrebla longirostris* | 1 |
| *Strebla altmani* | 3 |
| *Strebla alvarezi* | 4 |
| *Strebla asternalis* | 1 |
| *Strebla choropteri* | 1 |
| *Strebla christinae* | 3 |
| *Strebla consocia* | 5 |
| *Strebla cormurae* | 1 |
| *Strebla curvata* | 5 |
| *Strebla diaemi* | 1 |
| *Strebla diphyllae* | 1 |
| *Strebla galindoi* | 1 |
| *Strebla guajiro* | 3 |
| *Strebla harderi* | 2 |
| *Strebla hertigi* | 1 |
| *Strebla kohlsi* | 1 |
| *Strebla machadoi* | 1 |
| *Strebla matsoni* | 2 |
| *Strebla mirabilis* | 6 |
| *Strebla obtusa* | 2 |
| *Strebla paramirabilis* | 3 |
| *Strebla proxima* | 2 |
| *Strebla tonatiae* | 4 |
| *Strebla wiedemanni* | 1 |
| *Trichobioides perspicillatus* | 1 |
| *Trichobius affinis* | 1 |
| *Trichobius anducei* | 1 |
| *Trichobius angulatus* | 1 |
| *Trichobius assimilis* | 2 |
| *Trichobius caecus* | **13** |
| *Trichobius costalimai* | 2 |
| *Trichobius diaemi* | 1 |
| *Trichobius diphyllae* | 1 |
| *Trichobius dugesii* | 5 |
| *Trichobius dugesioides* | **10** |
| *Trichobius ethophallus* | 1 |
| *Trichobius flagellatus* | 2 |
| *Trichobius galei* | 1 |
| *Trichobius handleyi* | 2 |
| *Trichobius hispidus* | 1 |
| *Trichobius imitator* | 1 |
| *Trichobius joblingi* | 6 |
| *Trichobius johnsonae* | 4 |
| *Trichobius jubatus* | 1 |
| *Trichobius keemani* | 3 |
| *Trichobius leiomotus* | 1 |
| *Trichobius lionycteridis* | 5 |
| *Trichobius lonchophyllae* | 5 |
| *Trichobius longipilis* | 2 |
| *Trichobius macrophylli* | 1 |
| *Trichobius mendezi* | 4 |
| *Trichobius pallidus* | 1 |
| *Trichobius parasiticus* | 1 |
| *Trichobius parasparsus* | 5 |
| *Trichobius permilis* | 3 |
| *Trichobius petersoni* | 2 |
| *Trichobius propinquus* | 1 |
| *Trichobius silvicolae* | 2 |
| *Trichobius sparsus* | 2 |
| *Trichobius sphaeronotus* | 4 |
| *Trichobius strictisternus* | 1 |
| *Trichobius tiptoni* | 5 |
| *Trichobius tuttlei* | 1 |
| *Trichobius uniformis* | 5 |
| *Trichobius urodermae* | 1 |
| *Trichobius vampyropis* | 1 |
| *Trinchobius longiceps* | 7 |
| *Xenotrichobius noctilionis* | 2 |

**Table S4.** Centrality metrics of the species of bats.

| **Species bats** | **Degree centrality (DC)** |
| --- | --- |
| *Ametrida centurio* | 1 |
| *Anoura caudifer* | 3 |
| *Anoura cultrata* | 2 |
| *Anoura geoffroyi* | **7** |
| *Anoura latidens* | 2 |
| *Anoura* sp. | 4 |
| *Artibeus fuliginosus* | 4 |
| *Artibeus jamaicensis* | **13** |
| *Artibeus lituratus* | **8** |
| *Artibeus obscurus* | 2 |
| *Artibeus planirostris* | 2 |
| *Artibeus* sp. | 1 |
| *Carollia brevicauda* | **10** |
| *Carollia perspicillata* | **14** |
| *Carollia* sp. | 1 |
| *Chiroderma salvini* | 1 |
| *Chiroderma trinitatum* | 1 |
| *Chiroderma villosum* | 2 |
| *Chrotopterus auritus* | 3 |
| *Cormura brevirostris* | 1 |
| *Cynomops planirostris* | 1 |
| *Dermanura cinerea* | 4 |
| *Desmodus rotundus* | **11** |
| *Diaemus youngii* | 2 |
| *Diphylla ecaudata* | 3 |
| *Eptesicus brasiliensis* | 1 |
| *Eptesicus furinalis* | 1 |
| *Eptesicus fuscus* | 1 |
| *Eptesicus orinocensis* | 2 |
| *Enchisthenes hartii* | 3 |
| *Eumops glaucinus* | 2 |
| *Furipterus horrens* | 1 |
| *Gardnerycteris crenulatum* | 2 |
| *Glossophaga longirostris* | 5 |
| *Glossophaga soricina* | 4 |
| *Lionycteris spurrelli* | 2 |
| *Lonchophylla orienticollina* | 2 |
| *Lonchophylla robusta* | 4 |
| *Lonchophylla thomasi* | 1 |
| *Lonchorhina aurita* | 3 |
| *Lonchorhina orinocensis* | 4 |
| *Lophostoma brasiliense* | 4 |
| *Lophostoma carrikeri* | 5 |
| *Lophostoma silvicolum* | 5 |
| *Macrophyllum macrophyllum* | 5 |
| *Mesoophylla macconelli* | 1 |
| *Lampronycteris brachyotis* | 1 |
| *Micronycteris megalotis* | 2 |
| *Micronycteris microtis* | 2 |
| *Micronycteris minuta* | 2 |
| *Molossus aztecus* | 2 |
| *Molossus molossus* | 2 |
| *Molossus rufus* | 6 |
| *Mormoops megalophylla* | 1 |
| *Myotis albescens* | 2 |
| *Myotis handleyi* | 2 |
| *Myotis nigricans* | 4 |
| *Myotis oxyotus* | 2 |
| *Myotis riparius* | 3 |
| *Myotis* sp. | 2 |
| *Natalus tumidirostris* | 2 |
| *Noctilio albiventris* | **10** |
| *Noctilio leporinus* | 4 |
| *Nyctinomops laticaudatus* | 1 |
| *Peropteryx macrotis* | 4 |
| *Peropteryx trinitatis* | 1 |
| *Phylloderma stenops* | 1 |
| *Phyllostomus discolor* | 6 |
| *Phyllostomus elongatus* | **11** |
| *Phyllostomus hastatus* | **10** |
| *Platyrrhinus aurarinus* | 1 |
| *Platyrrhinus aurarius* | 1 |
| *Platyrrhinus helleri* | **8** |
| *Platyrrhinus vittatus* | 1 |
| *Platyrrhynus auraritus* | 2 |
| *Pteronotus davyi* | 2 |
| *Pteronotus parnellii* (posible *P. fuscus*) | **7** |
| *Pteronotus personatus* | 1 |
| *Pteronotus rubiginosus* | 1 |
| *Pteronotus suapurensis* | 1 |
| *Pteropteryx macrotis* | 1 |
| *Rhinophylla pumilio* | 1 |
| *Rhynchonycteris naso* | 3 |
| *Saccopteryx bilineata* | 3 |
| *Sphaeronycteris toxophyllum* | 1 |
| *Sturnira bidens* | 1 |
| *Sturnira bogotensis* | 1 |
| *Sturnira erythromos* | 1 |
| *Sturnira giannae* | 3 |
| *Sturnira lilium* (possible *S. giannae*) | 5 |
| *Sturnira ludovici* | 3 |
| *Sturnira parvidens* | 2 |
| *Sturnira* sp. | 1 |
| *Sturnira tildae* | 3 |
| *Tonatia bidens* | 1 |
| *Tonatia maresi* | 3 |
| *Trachops cirrhosus* | 6 |
| *Trinycteris nicefori* | 3 |
| *Uroderma bilobatum* | **9** |
| *Uroderma magnirostrum* | **5** |
| *Vampyriscus bidens* | 1 |
| *Vampyrodes caraccioli* | 1 |
| *Platyrrhinus dorsalis* | 1 |
|  |  |

**Table S5**. Metrics of the interaction networks for the study areas, the first landscape: Herbaceous plains and chaparral – HPC and Orinoco-Amazonian Forests - OAF in the Colombian and Venezuelan Orinoquia Region.

| **Metrics** | **OAF** | **HPC** |
| --- | --- | --- |
| Bat species | 71 | 78 |
| Fly species | 91 | 93 |
| Links | 195 | 205 |
| Specialization (*H_2_'*) | 0.56 | 0.62 |
| Number of modules | 13 | 16 |
| Modularity (*Q*) | 0.73 | 0.79 |

**Table S6**. Centrality metric of the species of ectoparasitic flies, the first landscape: Herbaceous plains and chaparral – HPC and Orinoco-Amazonian Forests - OAF in the Colombian and Venezuelan Orinoquia Region.

| **Species ectoparasitic fly** | **HPC** | **OAF** |
| --- | --- | --- |
| *Anastrebla caudiferae* | 1 | 1 |
| *Anastrebla modestini* | 1 | 2 |
| *Anatrichobius scorzai* | 1 | 1 |
| *Aspidoptera falcata* | 4 | 6 |
| *Aspidoptera phyllostomatis* | 5 | 1 |
| *Basilia ferrisi* | 1 | 1 |
| *Basilia ortizi* | 2 | 1 |
| *Exastinion clovisi* | 3 | 3 |
| *Mastoptera minuta* | 3 | 9 |
| *Megistopoda aranea* | 3 | 1 |
| *Metalasmus pseudopterus* | 12 | 12 |
| *Neotrichobius delicatus* | 1 | 4 |
| *N. coxata* | 1 | 3 |
| *Nycterophilia parnelli* | 2 | 2 |
| *Paradyschiria fusca* | 1 | 1 |
| *Paradyschiria parvula* | 1 | 3 |
| *Paraeuctenoides longipes* | 2 | 3 |
| *Paratrichobius dunni* | 1 | 3 |
| *Paratrichobius longicrus* | 2 | 2 |
| *Paratrichobius salvini* | 4 | 4 |
| *Pseudostrebla greenwelli* | 1 | 1 |
| *Pseudostrebla sparsisetis* | 1 | 1 |
| *Speiseria ambigua* | 2 | 1 |
| *Speseria peytoni* | 1 | 1 |
| *Strebla altmani* | 2 | 3 |
| *Strebla alvarezi* | 1 | 3 |
| *Strebla christinae* | 1 | 3 |
| *Strebla consocia* | 3 | 4 |
| *Strebla galindoi* | 1 | 1 |
| *Strebla guajiro* | 3 | 1 |
| *Strebla hertigi* | 2 | 1 |
| *Strebla machadoi* | 1 | 1 |
| *Strebla matsoni* | 1 | 2 |
| *Strebla mirabilis* | 3 | 5 |
| *Strebla proxima* | 2 | 1 |
| *Strebla tonatiae* | 3 | 2 |
| *Strebla wiedemanni* | 1 | 1 |
| *Trichobioides perspicillatus* | 1 | 1 |
| *Trichobius affinis* | 1 | 1 |
| *Trichobius costalimai* | 2 | 1 |
| *Trichobius dugesii* | 3 | 2 |
| *Trichobius dugesioides* | 6 | 7 |
| *Trichobius ethophallus* | 1 | 1 |
| *Trichobius flagellatus* | 1 | 2 |
| *Trichobius galei* | 1 | 1 |
| *Trichobius handleyi* | 1 | 2 |
| *Trichobius joblingi* | 6 | 1 |
| *Trichobius johnsonae* | 4 | 1 |
| *Trichobius keemani* | 2 | 2 |
| *Trichobius leiomotus* | 1 | 1 |
| *Trichobius lionycteridis* | 1 | 5 |
| *Trichobius macrophylli* | 1 | 1 |
| *Trichobius pallidus* | 7 | 9 |
| *Trichobius parasiticus* | 1 | 1 |
| *Trichobius permilis* | 2 | 2 |
| *Trichobius sparsus* | 1 | 6 |
| *Trichobius tiptoni* | 5 | 1 |
| *Trichobius uniformis* | 2 | 2 |
| *Trichobius urodermae* | 1 | 1 |
| *Trinchobius longiceps* | 5 | 5 |
| *Xenotrichobius noctilionis* | 1 | 1 |
| *Anastrebla mattadeni* | 1 | NA |
| *Anastrebla nycteridis* | 2 | NA |
| *Aspidoptera delatorrei* | 6 | NA |
| *Basilia dubia* | 1 | NA |
| *Basilia juquiensis* | 1 | NA |
| *Basilia myotis* | 6 | NA |
| *Basilia tiptoni* | 1 | NA |
| *Basilia wenzeli* | 1 | NA |
| *Exastinion deceptivum* | 1 | NA |
| *Exastinion oculatum* | 1 | NA |
| *Mastoptera guimaraesi* | 1 | NA |
| *Megistopoda proxima* | 5 | NA |
| *Neotrichobius stenopterus* | 1 | NA |
| *Noctiliostrebla aitkeni* | 1 | NA |
| *Noctiliostrebla maai* | 3 | NA |
| *Paradyschiria curvata* | 4 | NA |
| *Paradyschiria lineata* | 3 | NA |
| *Paradyschiria parvuloides* | 1 | NA |
| *Paratrichobius sanchezi* | 1 | NA |
| *Strebla diphyllae* | 1 | NA |
| *Trichobius anducei* | 1 | NA |
| *Trichobius diphyllae* | 1 | NA |
| *Trichobius hispidus* | 1 | NA |
| *Trichobius jubatus* | 1 | NA |
| *Trichobius lonchophyllae* | 5 | NA |
| *Trichobius mendezi* | 4 | NA |
| *Trichobius parasparsus* | 1 | NA |
| *Trichobius petersoni* | 2 | NA |
| *Trichobius sphaeronotus* | 4 | NA |
| *Trichobius vampyropis* | 1 | NA |
| *Strebla curvata* | 3 | NA |
| *Anastrebla spurrelli* | NA | 2 |
| *Basilia bequaerti* | NA | 1 |
| *Basilia tuttlei* | NA | 1 |
| *Basilia typhlops* | NA | 1 |
| *Neotrichobius bisetosus* | NA | 2 |
| *Neotrichobius ectophyllae* | NA | 1 |
| *Paraeuctenoides similis* | NA | 1 |
| *Parastrebla handleyi* | NA | 1 |
| *Paratrichobius lowei* | NA | 1 |
| *Pseudostrebla ribeiroi* | NA | 1 |
| *Speiseria magnioculus* | NA | 1 |
| *Stizostrebla longirostris* | NA | 1 |
| *Strebla asternalis* | NA | 1 |
| *Strebla choropteri* | NA | 1 |
| *Strebla cormurae* | NA | 1 |
| *Strebla curvata* | NA | 2 |
| *Strebla harderi* | NA | 2 |
| *Strebla kohlsi* | NA | 1 |
| *Strebla obtusa* | NA | 2 |
| *Strebla paramirabilis* | NA | 3 |
| *Trichobius angulatus* | NA | 1 |
| *Trichobius assimilis* | NA | 2 |
| *Trichobius diaemi* | NA | 1 |
| *Trichobius imitator* | NA | 1 |
| *Trichobius longipilis* | NA | 2 |
| *Trichobius propinquus* | NA | 1 |
| *Trichobius silvicolae* | NA | 2 |
| *Trichobius strictisternus* | NA | 1 |
| *Trichobius tuttlei* | NA | 1 |

**Table S7**. Centrality metric of the species of bats, the first landscape: Herbaceous plains and chaparral – HPC and Orinoco-Amazonian Forests - OAF in the Colombian and Venezuelan Orinoquia Region.

| **Species bat** | **HPC** | **OAF** |
| --- | --- | --- |
| *Anoura caudifer* | 3 | 2 |
| *Anoura geoffroyi* | 3 | 6 |
| *Artibeus jamaicensis* | 4 | 11 |
| *Artibeus lituratus* | 4 | 6 |
| *Carollia brevicauda* | 9 | 2 |
| *Carollia perspicillata* | 11 | 7 |
| *Chiroderma villosum* | 2 | 1 |
| *Chrotopterus auritus* | 1 | 3 |
| *Dermanura cinerea* | 1 | 2 |
| *Desmodus rotundus* | 9 | 5 |
| *Eptesicus furinalis* | 1 | 1 |
| *Glossophaga longirostris* | 4 | 2 |
| *Glossophaga soricina* | 3 | 2 |
| *Lonchorhina aurita* | 3 | 2 |
| *Lonchorhina orinocensis* | 3 | 3 |
| *Lophostoma brasiliense* | 3 | 4 |
| *Lophostoma carrikeri* | 1 | 5 |
| *Lophostoma silvicolum* | 1 | 5 |
| *Macrophyllum macrophyllum* | 4 | 3 |
| *Micronycteris megalotis* | 2 | 1 |
| *Micronycteris minuta* | 2 | 2 |
| *Molossus rufus* | 5 | 1 |
| *Mormoops megalophylla* | 1 | 1 |
| *Myotis albescens* | 2 | 1 |
| *Myotis nigricans* | 3 | 3 |
| *Myotis riparius* | 2 | 1 |
| *Natalus tumidirostris* | 1 | 2 |
| *Noctilio albiventris* | 10 | 2 |
| *Noctilio leporinus* | 3 | 3 |
| *Peropteryx macrotis* | 1 | 5 |
| *Phylloderma stenops* | 1 | 1 |
| *Phyllostomus discolor* | 5 | 4 |
| *Phyllostomus elongatus* | 5 | 9 |
| *Phyllostomus hastatus* | 5 | 7 |
| *Platyrrhinus helleri* | 5 | 3 |
| *Pteronotus parnellii* (posible *P. fuscus*) | 7 | 4 |
| *Pteronotus personatus* | 1 | 1 |
| *Saccopteryx bilineata* | 1 | 2 |
| *Sturnira lilium* (probable *S. giannae)* | 3 | 3 |
| *Tonatia bidens* | 1 | 1 |
| *Trachops cirrhosus* | 4 | 4 |
| *Uroderma bilobatum* | 4 | 5 |
| *Uroderma magnirostrum* | 1 | 4 |
| *Anoura cultrata* | 2 | NA |
| *Anoura latidens* | 2 | NA |
| *Artibeus obscurus* | 2 | NA |
| *Artibeus planirostris* | 2 | NA |
| *Chiroderma salvini* | 1 | NA |
| *Chiroderma trinitatum* | 1 | NA |
| *Diphylla ecaudata* | 3 | NA |
| *Enchisthenes hartii* | 3 | NA |
| *Eptesicus fuscus* | 1 | NA |
| *Eptesicus orinocensis* | 2 | NA |
| *Gardnerycteris crenulatum* | 2 | NA |
| *Lonchophylla orienticolina* | 2 | NA |
| *Lonchophylla robusta* | 4 | NA |
| *Molossus molossus* | 2 | NA |
| *Myotis handleyi* | 2 | NA |
| *Peropteryx trinitatis* | 1 | NA |
| *Phalcophila puliciformis* | 1 | NA |
| *Platyrrhinus vittatus* | 1 | NA |
| *Pteronotus davyi* | 2 | NA |
| *Pteronotus fuscus* | 1 | NA |
| *Sturnira bidens* | 1 | NA |
| *Sturnira bogotensis* | 1 | NA |
| *Sturnira* cf. *parvidens* | 2 | NA |
| *Sturnira erythromos* | 1 | NA |
| *Sturnira giannae* | 3 | NA |
| *Sturnira ludovici* | 3 | NA |
| *Sturnira parvidens* | 2 | NA |
| *Tonatia maresi* | 3 | NA |
| *Platyrrhinus dorsalis* | 1 | NA |
| *Ametrida centurio* | NA | 1 |
| *Artibeus fuliginosus* | NA | 4 |
| *Diaemus youngii* | NA | 2 |
| *Eptesicus brasiliensis* | NA | 1 |
| *Eptesicus brasiliensis* | NA | 1 |
| *Eumops glaucinus* | NA | 2 |
| *Furipterus horrens* | NA | 1 |
| *Lionycteris spurrelli* | NA | 2 |
| *Lonchophylla thomasi* | NA | 1 |
| *Mesophylla macconelli* | NA | 1 |
| *Micronycteris microtis* | NA | 2 |
| *Molossus aztecus* | NA | 2 |
| *Myotis oxyotus* | NA | 3 |
| *Nyctinomops laticaudatus* | NA | 1 |
| *Platyrrhinus aurarius* | NA | 4 |
| *Pteronotus rubiginosus* | NA | 1 |
| *Rhinophylla pumilio* | NA | 1 |
| *Rhynchonycteris naso* | NA | 3 |
| *Sphacronycteris toxophyllum* | NA | 1 |
| *Sturnira tildae* | NA | 3 |
| *Uroderma* | NA | 4 |
| *Vampyriscus bidens* | NA | 1 |
| *Vampyrodes caraccioli* | NA | 1 |
| *Trinycteris nicefori* | NA | 3 |
| *Cormura brevirostris* | NA | 1 |
