# Supplementary figures and images for: ﻿Exploring the relationship between bats (Mammalia, Chiroptera) and ectoparasitic flies (Diptera, Hippoboscoidea) of the Orinoquia Region in South America
Source: Zookeys. 2023 Sep 8;1179:1–34. doi: 10.3897/zookeys.1179.103479 (PMC10504637; doi:10.3897/zookeys.1179.103479)

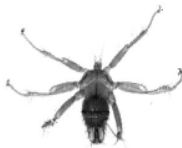

BAT FLIES

HOST

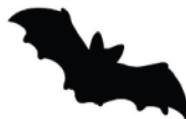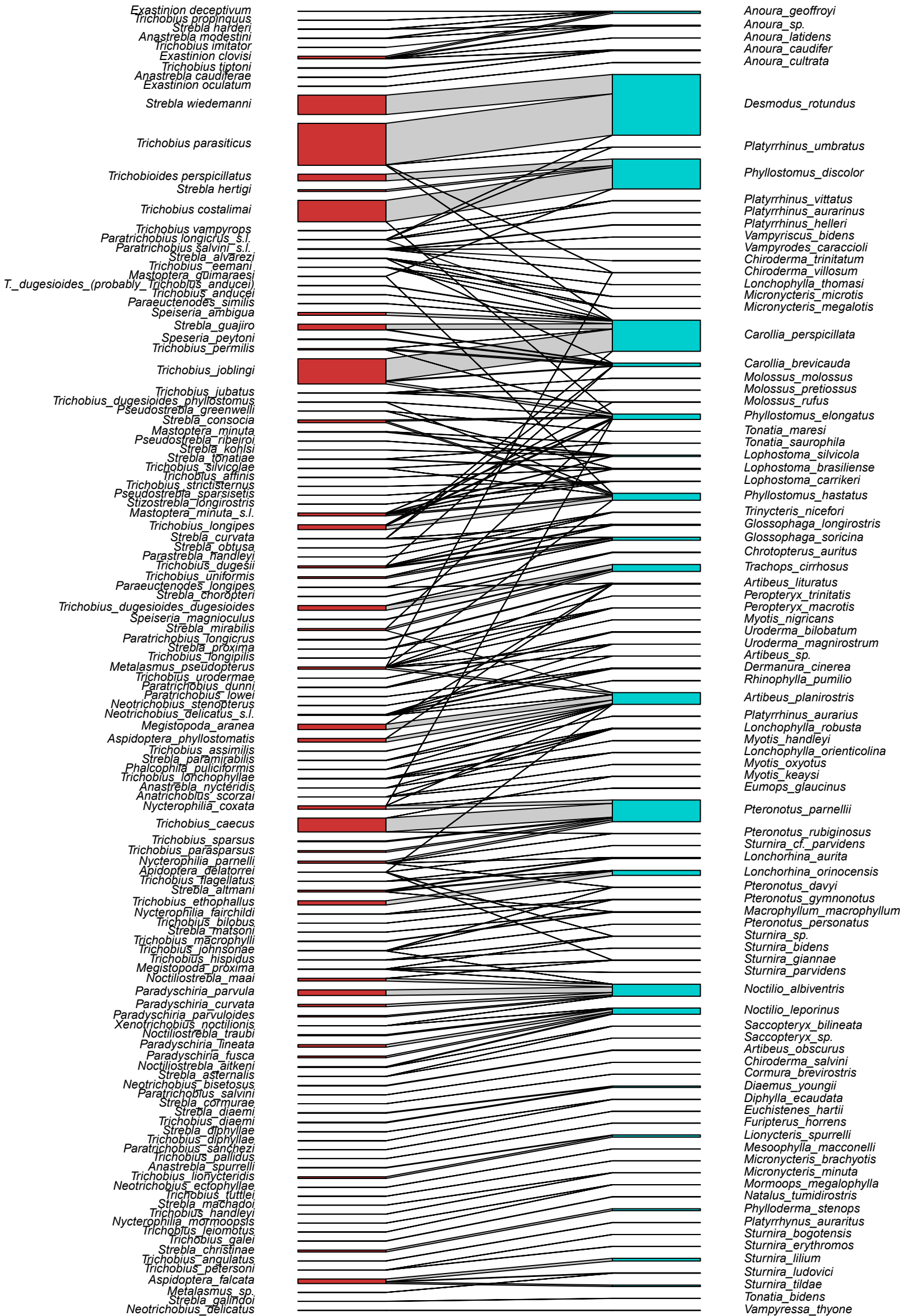

Supplement: Supplementary material 2 — Bipartite bat-fly quantitative network of the Herbaceous plains and chaparral – HPC [file zookeys-1179-001_article-103479__-s002.pdf]
