# Supplementary material for: ﻿Exploring the relationship between bats (Mammalia, Chiroptera) and ectoparasitic flies (Diptera, Hippoboscoidea) of the Orinoquia Region in South America
Source: Zookeys. 2023 Sep 8;1179:1–34. doi: 10.3897/zookeys.1179.103479 (PMC10504637; doi:10.3897/zookeys.1179.103479)
Supplement: Supplementary material 3 — Bipartite bat-fly quantitative network of the Orinoco-Amazonian Forests – OAF [file zookeys-1179-001_article-103479__-s003.pdf]

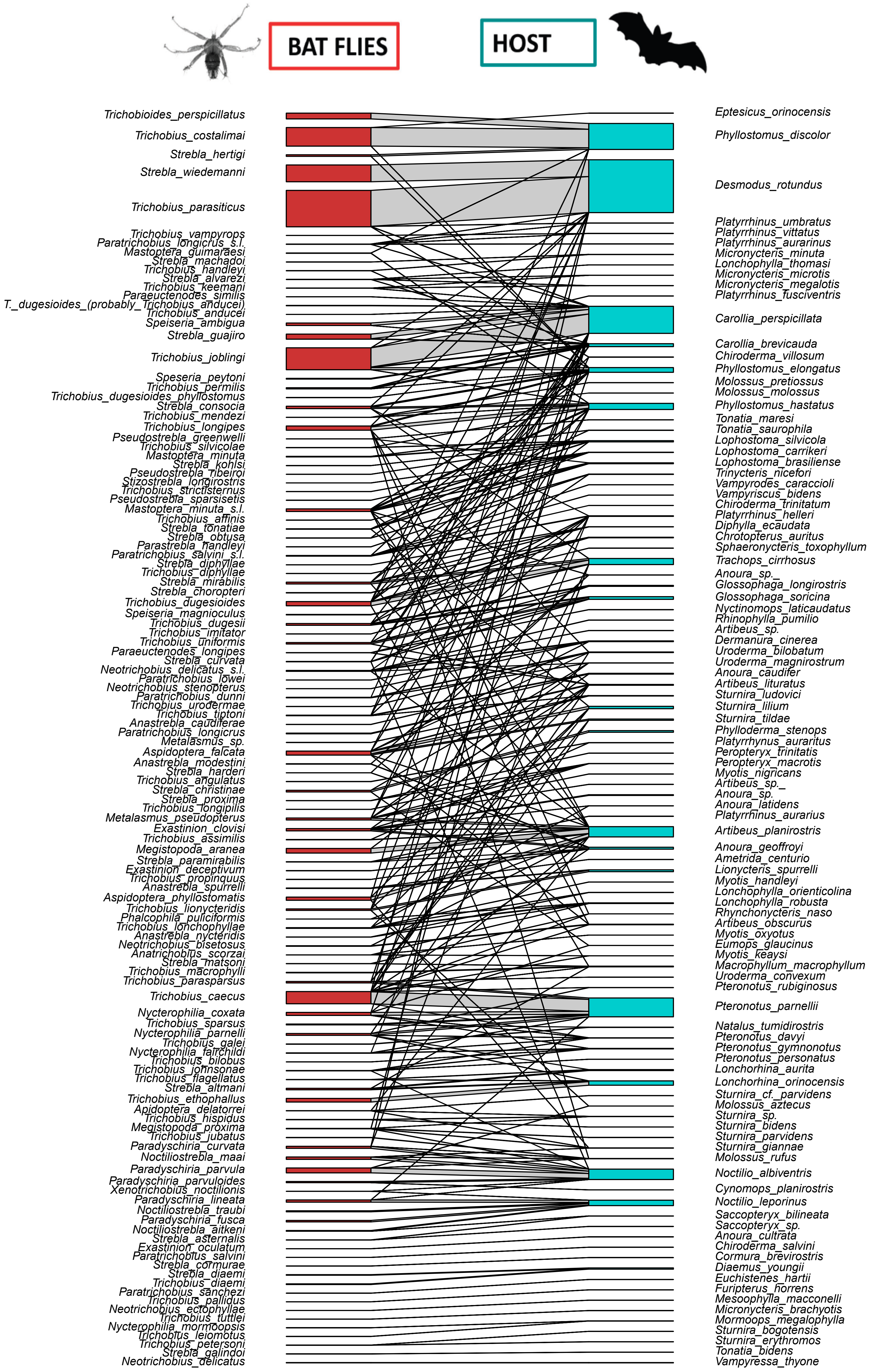

BAT FLIES
HOST
Trichobioides_perspicillatus
Trichobius_costalimai
Strebla_hertigi
Strebla_wiedemanni
Trichobius_parasiticus
Trichobius_vampyrops
Paratrichobius_longicrus_s.l.
Mastoptera_guimaraesi
Strebla_machadoi
Trichobius_handleyi
Strebla_alvarezi
Trichobius_keenani
Paraeuctenodes_similis
T._dugesioides_(probably_Trichobius_anducei)
Trichobius_anducei
Speiseria_ambigua
Strebla_guajiro
Trichobius_joblingi
Speseria_peytoni
Trichobius_permilis
Trichobius_dugesioides_phyllostomus
Strebla_consocia
Trichobius_mendezi
Trichobius_longipes
Pseudostrebla_greenwelli
Trichobius_silvicolae
Mastoptera_minuta
Strebla_kohlsi
Pseudostrebla_ribeiroi
Stizostrebla_longirostris
Trichobius_strictisternus
Pseudostrebla_sparsisetis
Mastoptera_minuta_s.l.
Trichobius_affinis
Strebla_tonatiae
Strebla_obtusa
Parastrebla_handleyi
Paratrichobius_salvini_s.l.
Strebla_diphyllae
Trichobius_diphyllae
Strebla_mirabilis
Strebla_choropteri
Trichobius_dugesioides
Speiseria_magnioculus
Trichobius_dugesii
Trichobius_imitator
Trichobius_uniformis
Paraeuctenodes_longipes
Strebla_curvata
Neotrichobius_delicatus_s.l.
Paratrichobius_lowei
Neotrichobius_stenopterus
Paratrichobius_dunni
Trichobius_urodermae
Trichobius_tiptoni
Anastrebla_caudiferae
Paratrichobius_longicrus
Metalasmus_sp.
Aspidoptera_falcata
Anastrebla_modestini
Strebla_harderi
Trichobius_angulatus
Strebla_christinae
Strebla_proxima
Trichobius_longipilis
Metalasmus_pseudopterus
Exastinion_clovisi
Trichobius_assimilis
Megistopoda_aranea
Strebla_paramirabilis
Exastinion_deceptivum
Trichobius_propinquus
Anastrebla_spurrelli
Aspidoptera_phyllostomatis
Trichobius_lionycteridis
Phalcophila_puliciformis
Trichobius_lonchophyllae
Anastrebla_nycteridis
Neotrichobius_bisetosus
Anatrichobius_scorzai
Strebla_matsoni
Trichobius_macrophylli
Trichobius_parasparsus
Trichobius_caecus
Nycterophilia_coxata
Trichobius_sparsus
Nycterophilia_parnelli
Trichobius_galei
Nycterophilia_fairchildi
Trichobius_bilobus
Trichobius_johnsonae
Trichobius_flagellatus
Strebla_altmani
Trichobius_ethophallus
Apidoptera_delatorrei
Trichobius_hispidus
Megistopoda_proxima
Trichobius_jubatus
Paradyschiria_curvata
Noctiliostrebla_maai
Paradyschiria_parvula
Paradyschiria_parvuloides
Xenotrichobius_noctilionis
Paradyschiria_lineata
Noctiliostrebla_traubi
Paradyschiria_fusca
Noctiliostrebla_aitkeni
Strebla_asternalis
Exastinion_oculatum
Paratrichobius_salvini
Strebla_cormurae
Strebla_diaemi
Trichobius_diaemi
Paratrichobius_sanchezi
Trichobius_pallidus
Neotrichobius_ectophyllae
Trichobius_tuttlei
Nycterophilia_mormoopsis
Trichobius_leionotus
Trichobius_petersoni
Strebla_galindoi
Neotrichobius_delicatus
Eptesicus_orinocensis
Phyllostomus_discolor
Desmodus_rotundus
Platyrrhinus_umbratus
Platyrrhinus_vittatus
Platyrrhinus_aurarinus
Micronycteris_minuta
Lonchophylla_thomasi
Micronycteris_microtis
Micronycteris_megalotis
Platyrrhinus_fusciventris
Carollia_perspicillata
Carollia_brevicauda
Chiroderma_villosum
Phyllostomus_elongatus
Molossus_pretiossus
Molossus_molossus
Phyllostomus_hastatus
Tonatia_maresi
Tonatia_saurophila
Lophostoma_silvicola
Lophostoma_carrikeri
Lophostoma_brasiliense
Trinycteris_nicefori
Vampyrodes_caraccioli
Vampyriscus_bidens
Chiroderma_trinitatum
Platyrrhinus_helleri
Diphylla_ecaudata
Chrotopterus_auritus
Sphaeronycteris_toxophyllum
Trachops_cirrhosus
Anoura_sp._
Glossophaga_longirostris
Glossophaga_soricina
Nyctinomops_laticaudatus
Rhinophylla_pumilio
Artibeus_sp.
Dermanura_cinerea
Uroderma_bilobatum
Uroderma_magnirostrum
Anoura_caudifer
Artibeus_lituratus
Sturnira_ludovici
Sturnira_lilium
Sturnira_tildae
Phylloderma_stenops
Platyrrhynus_aurarius
Peropteryx_trinitatis
Peropteryx_macrotis
Myotis_nigricans
Artibeus_sp._
Anoura_sp.
Anoura_latidens
Platyrrhinus_aurarius
Artibeus_planirostris
Anoura_geoffroyi
Ametrida_centurio
Lionycteris_spurrelli
Myotis_handleyi
Lonchophylla_orienticolina
Lonchophylla_robusta
Rhynchonycteris_naso
Artibeus_obscurus
Myotis_oxyotus
Eumops_glaucinus
Myotis_keaysi
Macrophyllum_macrophyllum
Uroderma_convexum
Pteronotus_rubiginosus
Pteronotus_parnellii
Natalus_tumidirostris
Pteronotus_davyi
Pteronotus_gymnonotus
Pteronotus_personatus
Lonchorhina_aurita
Lonchorhina_orinocensis
Sturnira_cf._parvidens
Molossus_aztecus
Sturnira_sp.
Sturnira_bidens
Sturnira_parvidens
Sturnira_giannae
Molossus_rufus
Noctilio_albiventris
Cynomops_planirostris
Noctilio_leporinus
Saccopteryx_bilineata
Saccopteryx_sp.
Anoura_cultrata
Chiroderma_salvini
Cormura_brevirostris
Diaemus_youngii
Euchistenes_hartii
Furipterus_horrens
Mesoophylla_macconelli
Micronycteris_brachyotis
Mormoops_megalophylla
Sturnira_bogotensis
Sturnira_erythromos
Tonatia_bidens
Vampyressa_thyone

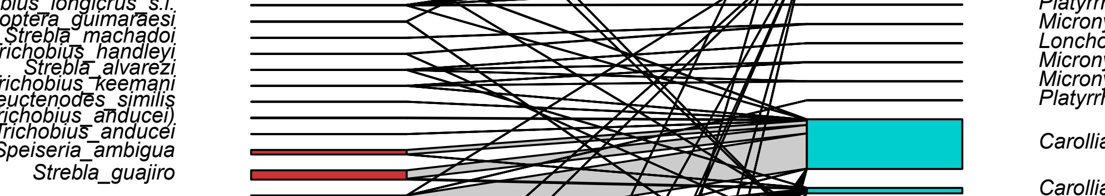
Strebla_machadoi
Strebla_alvarezi
Trichobius_anducei
Speiseria_ambigua
Strebla_guajiro

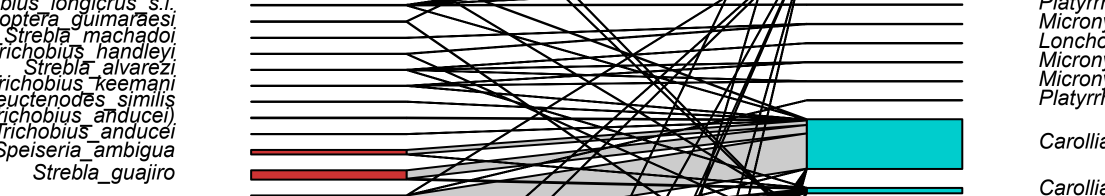

bius_longicrus_s.l.
optera_guimaraesi
Strebla_machadoi
richobius_handleyi
Strebla_alvarezi
richobius_keemani
euctenodes_similis
richobius_anducei)
Trichobius_anducei
Speiseria_ambigua
Strebla_guajiro
Platyrrh
Microny
Loncho
Microny
Microny
Platyrrh
Carollia
Carollia
